# Integrative Network Analysis Revealed Genetic Impact of Pyruvate Kinase L/R on Hepatocyte Proliferation and Graft Survival after Liver Transplantation

**DOI:** 10.1155/2021/7182914

**Published:** 2021-09-02

**Authors:** Zhengtao Liu, Junsheng Zhao, Wenchao Wang, Hai Zhu, Junjie Qian, Shuai Wang, Shuping Que, Feng Zhang, Shengyong Yin, Lin Zhou, Lei Geng, Shusen Zheng

**Affiliations:** ^1^Division of Hepatobiliary and Pancreatic Surgery, Department of Surgery, First Affiliated Hospital, School of Medicine, Zhejiang University, Hangzhou 310003, China; ^2^NHC Key Laboratory of Combined Multi-Organ Transplantation, Key Laboratory of the Diagnosis and Treatment of Organ Transplantation, CAMS, First Affiliated Hospital, School of Medicine, Zhejiang University, 310003 Hangzhou, China; ^3^Key Laboratory of Organ Transplantation, Zhejiang Province, First Affiliated Hospital, School of Medicine, Zhejiang University, 310003 Hangzhou, China; ^4^State Key Laboratory for Diagnosis and Treatment of Infectious Diseases, National Clinical Research Center for Infectious Diseases, Collaborative Innovation Center for Diagnosis and Treatment of Infectious Diseases, The First Affiliated Hospital, Zhejiang University School of Medicine, 310003 Hangzhou, China; ^5^Department of Hepatobiliary Surgery, First Affiliated Hospital of Guangxi Medical University, 530021 Nanning, China; ^6^DingXiang Clinics, 310 000 Hangzhou, China; ^7^Shulan Hospital (Hangzhou), 310 000 Hangzhou, China

## Abstract

**Background:**

Pyruvate kinase L/R (PKLR) has been suggested to affect the proliferation of hepatocytes via regulation of the cell cycle and lipid metabolism. However, its impact on the global metabolome and its clinical implications remain unclear.

**Aims:**

We aimed to clarify the genetic impact of PKLR on the metabolomic profiles of hepatoma cells and its potential effects on grafts for liver transplantation (LT).

**Methods:**

Nontargeted and targeted metabolomic assays were performed in human hepatoma cells transfected with lentiviral vectors causing PKLR overexpression and silencing, respectively. We then constructed a molecular network based on integrative analysis of transcriptomic and metabolomic data. We also assessed the biological functions of PKLR in the global metabolome in LT grafts in patients via a weighted correlation network model.

**Results:**

Multiomic analysis revealed that PKLR perturbations significantly affected the pyruvate, citrate, and glycerophospholipid metabolism pathways, as crucial steps in de novo lipogenesis (DNL). We also confirmed the importance of phosphatidylcholines (PC) and its derivative lyso-PC supply on improved survival of LT grafts in patients. Coexpression analysis revealed beneficial effects of PKLR overexpression on posttransplant prognosis by alleviating arachidonic acid metabolism of the grafts, independent of operational risk factors.

**Conclusion:**

This systems-level analysis indicated that PKLR affected hepatoma cell viability via impacts on the whole process of DNL, from glycolysis to final PC synthesis. PKLR also improved prognosis after LT, possibly via its impact on the increased genesis of beneficial glycerophospholipids.

## 1. Introduction

The liver acts as the central hub organ for the complex energy metabolism networks in human bodies [1]. Disruptions to lipid and glucose metabolism may have comprehensive interrelated effects on many physiological and pathological conditions in the liver. Energy metabolism can regulate hepatocyte inflammation, proliferation, and apoptosis by affecting the fuel provision, and the metabolic network has been shown to be involved in the whole profile of morbidity, from simple steatosis to end-stage liver malignancy [2].

Pyruvate kinase (PK) is a vital rate-limiting enzyme regulating glycolysis. PK catalyzes the biochemical process by transferring phosphate groups from phosphoenolpyruvate (PEP) to ADP, to yield ATP and pyruvate [3]. Pyruvate is located at the crossroads of energy metabolism and provides the raw material in the tricarboxylic acid (TCA) cycle to support cellular energy production. PK is encoded by different isoforms [4], of which PKM2 and PKLR are coexpressed in the liver (https://www.proteinatlas.org/). PKLR was proven to have a wide association with a spectrum of liver damage from steatosis and inflammation to fibrosis via its regulation on mitochondrial dysfunction and subsequent hepatic triglyceride accumulation, based on multiomic data at systematic levels [5, 6]. We also identified PKLR as a potential liver-specific target for treating hepatocellular carcinoma and nonalcoholic fatty liver disease [7]. Transcriptomic data further revealed that PKLR might affect cell viability via regulating liver mitochondrial function [8]. However, its potential effects on networks of metabolites with executive biological functions remain unclear and worthy of further investigation.

Liver transplantation (LT) provides the treatment of last resort for patients with end-stage liver disease, and graft quality is an important determinant predicting posttransplant prognosis [9, 10]. Energy metabolism has been shown to affect graft quality and postoperative outcomes after LT [11], and reduced pyruvate or increased lactate/pyruvate ratio predicted more severe ischemia/reperfusion damage in liver grafts [12, 13]. As a key gene involved in the regulation of glycolysis and intracellular mitochondrial function [8], we postulated a potential link between PKLR and energy output in liver grafts, which might affect posttransplant outcomes. Metabolomic analysis of grafts might help to elucidate the mechanism responsible for these effects.

Advanced high-throughput omics data provide a novel approach for unveiling the biological functions and molecular mechanisms involved in complex phenotypes, based on the genome, proteome, and metabolome [14, 15]. Integration of transcriptomic and metabolomic data might provide convincing evidence to allow the construction of a reliable network covering the biological process from the encoding gene to the final metabolic product, with mutual validation [16, 17]. Omics data might also be used to construct networks to improve the efficiency of diagnostic or prognostic predictions for specific diseases, by integrating clinical information from individual patients [17, 18]. Weighted gene coexpression network analysis (WGCNA) is a topological algorithm that can be used to investigate clinical-omics interactions in a scale-free network and is widely used in expression and metabolomic studies by modularization of coexpressed metabolites in clinical studies [19, 20]. The current study is aimed at applying integrative multiomics and the WGCNA algorithm to investigate the mechanism responsible for the genetic impact of PKLR on liver function, as well as its potential significance in terms of graft quality and posttransplant outcomes.

We assessed the molecular function of PKLR on global metabolites by integrating untargeted and targeted metabolomic and transcriptomic data in human hepatoma cells over- or underexpressing PKLR. We also evaluated the impact of PKLR on the LT graft metabolome, based on a scale-free network model. The results of this study might clarify the regulatory mechanism by which PKLR affects global liver metabolism and its potential effects on LT graft survival.

## 2. Materials and Methods

### 2.1. Study Design

A study-flow diagram is presented in Figure S1. HepG2 cells were transfected with short hairpin RNA (shRNA) and plasmids via lentiviral particles to silence or overexpress PKLR, respectively. Global and energy metabolites were measured in the transfected cells. Multiomic analysis was then performed by combining the results with previously published transcriptomic data for HepG2 cells with PKLR perturbation [8].

We also carried out untargeted metabolomic analysis in LT grafts and applied the WGCNA algorithm integrated with clinical information for patients who received LT grafts. Metabolites in positive modules were extracted for pathway analysis. Finally, hub metabolites were defined as overlapping differential substrates in cells and livers by classification of PKLR expression.

### 2.2. Cell Culture and Construction of Cellular Models with PKLR Perturbation

The effects of modification of PKLR expression were determined in HepG2 cells. In brief, HepG2 cells were cultured in RPMI-1640 medium (R8758; Sigma-Aldrich) including 10% fetal bovine serum (F8687, Sigma-Aldrich). The cells were placed in a humidified incubator at 37°C with 5% CO_2_.

PKLR expression levels in HepG2 cells were altered by lentiviral transfection, and the cells were subjected to subsequent metabolomic assay. Cells were transfected with lentiviral particles including an open reading frame clone or shRNA for PKLR, respectively, according to the manufacturer's instructions (RC212337L2V for overexpression (OV) and TL302463V for silencing (SI) of PKLR expression) (OriGene, USA). Corresponding control particles or scrambled shRNA in empty vectors served as internal references for transfection (PS100071V for OV (OV-NC), TR30021V for SI (SI-NC), OriGene). The shRNA sequence used for SI is presented in Table S1.

### 2.3. LT Cases and Clinical Information

All patients underwent LT during January 1^st^, 2015, and March 1^st^, 2019. Samples for the metabolomic assay were collected from wedge resections carried out for routine biopsy before transplantation. Exclusion criteria were (1) donor/recipient age < 18 years, (2) living donor LT, (3) split LT, (4) retransplantation, (5) multiorgan transplantation, (6) unavailable graft tissue, (7) unavailable graft RNA, and (8) loss to follow-up after LT. The study was carried out in accordance with the Declaration of Helsinki, and the study protocol was approved by the Institutional Review Board of our center (no. 2020-IIT-1063). Seventy-nine LT cases were finally enrolled for the metabolomic study.

Donor, recipient, allograft, and surgical information was collected retrospectively from medical records for each LT case. Follow-up visits to assess posttransplant survival status and duration of patients/grafts were managed by specialized staff.

Major complications, including early allograft dysfunction (EAD) and primary nonfunction (PNF), were also assessed in each recipient postoperatively, based on liver and coagulation function tests. More details of the definitions of EAD/PNF have been described previously [21]. Detailed clinical data and follow-up information for the enrolled cases are shown in Table 1.

### 2.4. Measurement of Key Gene Expression

Cells or tissues were resolved in the TRIzol reagent (15596026; Invitrogen) for RNA isolation. The subsequent RNA samples were purified using an RNeasy Mini Kit (74104; Qiagen), according to the manufacturer's instructions.

Expression of target genes was measured by quantitative real-time polymerase chain reaction (qRT-PCR) using SYBR Green Master Mix (1725121; Bio-Rad), with an integrative detection system (CFX96; Bio-Rad). Primer sequences are listed in Table S2. Gene expression was compared by the delta-delta Ct method, as described previously [22], with *β*-actin as an internal reference.

### 2.5. Metabolomic Analysis of Hepatoma Cells and Liver Tissues

Nontargeted metabolomic assays were performed using hepatoma cells and LT graft tissues. Metabolic profiling was analyzed by liquid chromatography-mass spectrometry (LC-MS) via coupled application of ultra-high-performance liquid chromatography (UHPLC) and QE plus system (Thermo Fisher Scientific, USA) in both electrospray ionization-positive and ionization-negative ion modes.

HepG2 cells were also assayed by energy-specific targeted metabolomic analysis by LC-MS via UHPLC coupled to a QTRAP system (AB Sciex, USA) based on multiple reaction monitoring [23]. Assays were performed based on the modules developed by Shanghai Applied Protein Technology Company, with coverage of 31 key metabolites in pathways including glycolysis, TCA, and oxidative phosphorylation. Information on the included energy metabolites is listed in Table S3.

### 2.6. Statistical Comparisons

Nonnormally distributed data were log-transformed. Normally distributed data were described as the mean ± standard deviation and compared by one-way ANOVA. Nonnormally distributed data were described as median/(interquartile range) and compared using the nonparametric Mann-Whitney *U* test. Relationships between variables were assessed by correlation analysis using Pearson's, Spearman's, and Kendall's coefficients for continuous, rank, and ordinal covariates, respectively. Survival analysis was performed using a Cox proportional hazards regression model. And interactions between key genes were imputed by a protein-protein interaction (PPI) network via the STRING database [24].

### 2.7. Principal Component Analysis (PCA)

PCA and orthogonal partial least-squares-discriminant analysis (OPLS-DA) were performed to evaluate the discrimination of metabolomic profiles separated by key traits using SIMCA-P software (version 14.1, Umetrics, Sweden).

### 2.8. Network Construction for LT Grafts

WGCNA has been shown to be suitable for constructing biological network models based on gene expression and metabolomic datasets [20]. We therefore used this algorithm to establish connections between PKLR expression and the global metabolome of LT grafts in a scale-free model [25].

The LT graft metabolome was first divided into different coexpressed modules, and the metabolite dendrogram branches were cut to produce merged modules based on thresholds defined by a degree of independence of 0.8. Different modules were indicated by different colors in a heatmap.

We then carried out a correlation analysis between merged modules and individual sample traits, including genetic and phenotypic expression. Clustering analysis was performed based on merged module categories for the 100 top selected metabolites.

The metabolite significance (MS) and module membership (MM) were defined as the correlation of each metabolite with individual traits (especially for PKLR expression) or module eigengene, respectively. The significance of the correlation between MS and MM was developed as an indicator to evaluate intramodule connectivity. We also assessed intermodule connectivity by correlation analysis. Significant modules were then selected for further pathway and hub metabolite investigation. More details of the application of WGCNA to metabolomic data have been published previously [20].

### 2.9. Enrichment, Pathway Analysis, and Network Construction Based on Multiomic Datasets

Based on the Kyoto Encyclopedia of Genes and Genomes (KEGG) database, clusters of potential metabolites were enriched for pathway imputation via the online toolkit “MetaboAnalyst” (https://www.metaboanalyst.ca/) [26]. Differentially expressed gene sets (DEGs) were imputed by the analysis of transcriptomic data for HepG2 cells with PKLR alterations in our previous study [8]. Potential pathways were also enriched by Gene Set Enrichment Analysis (GSEA) using the “clusterProfiler” package based on significant candidate genes detected by RNA-seq [27, 28]. Joint pathway analysis was then used to combine differentially expressed traits from multiomic datasets in cells with PKLR perturbations, and pathway maps integrating transcriptomics and metabolomic data were visualized using “Pathview” (https://pathview.uncc.edu/), according to the developer's instructions [29].

We also visualized the regulatory network and screening of hub metabolites using Cytoscape (v 3.8.0) [30]. Based on metabolites with significant internal correlations (*P* < 0.05), networks were constructed based on targeted (for cells) and untargeted (for cells/liver tissues) metabolomic analyses using the “cytoHubba” module, respectively. Hub metabolites were then screened using appropriate algorithms in the cytoHubba module. Regulatory networks were also incorporated based on metabolites correlated with key traits in prior pathway analysis.

Descriptive data and their interactive relationships were visualized using heatmaps, bar charts, Venn diagrams, and volcano, bubble, and scatter plots. Regarding the statistical methods, more details of the availability of the online and locally installed toolkits, packages, and algorithms used for data interpretation are listed in Table S4.

### 2.10. High-Glucose Treatment, Oil Red O Staining, and Biochemical Tests

Cellular models with PKLR perturbations (OV, SI, and corresponding NC, see Section 2.2) were prepared in six-well plates at 80% confluence and incubated in high-glucose medium (50 mmol/L for D-glucose, G7021; Sigma-Aldrich) for 48 h. The cells were then assayed for steatosis by oil red O staining, as described previously [31]. The severity of steatosis was assessed quantitatively as the ratio of the area between lipid droplets and hepatocytes in the same microscopic field. Meanwhile, cellular triglyceride (TG) and total cholesterol (TC) were measured, respectively, according to the instruction provided by commercial kits (E1013, E1015, Applygen). All experiments were repeated in triplicates.

## 3. Results

### 3.1. Creation of Cell Models with Altered PKLR Expression

Cell models were created by transfection with lentiviral particles to generate stable changes in PKLR expression. Compared with NC samples, transfection with open reading frame particles resulted in an increase of PKLP of about 4.7-fold, while transfection with shRNA caused an approximately 70% decrease in PKLR mRNA expression (both *P* < 0.05, Figure S2).

### 3.2. Clinical Features of LT Patients

The LT cases are described in Table 1. Briefly, most surgeries were performed in nonelderly adults (aged <60 years). Elderly recipients and donors accounted for 7.6% and 13.9% of the whole cohort. The average follow-up duration was around 10 months (308 days). Most recipients (79%) and 14% of donors were hepatitis B-positive.

### 3.3. Untargeted Metabolomic Profiling in Hepatoma Cells

**Figure 1:**
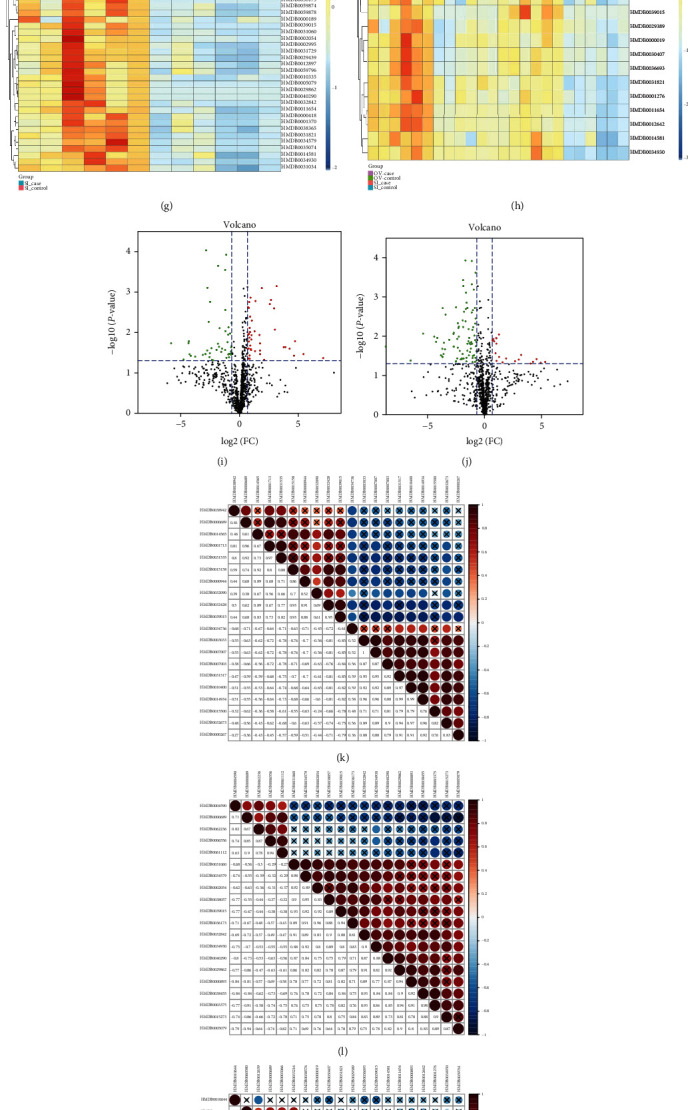
Comprehensive analysis on nontargeted metabolomic results in cells with PKLR variations. (a) PCA revealed clear separation in nontargeted metabolomic data from hepatocytes with overexpressed PKLR, dots in blue [2] represented the samples with overexpressed PKLR, and dots in green [1] represented the corresponded NC samples. (b) Validation of OPLS-DA model by class permutation analysis for (a). (c) PCA revealed clear separation in nontargeted metabolomic data from hepatocytes with overexpressed PKLR, dots in blue [2] represented the samples with downregulated PKLR, and dots in green [1] represented the corresponded NC samples. (d) Validation of OPLS-DA model by class permutation analysis for (c). (e) Correlation on FC of each metabolite in the group with PKLR overexpression and downregulation. (f) Heatmap of metabolites showed significant association with PKLR overexpression. (g) Heatmap of metabolites showed significant association with PKLR downregulation. (h) Heatmap of metabolites showed both significant associations with PKLR overexpression/downregulation. (i) Volcano plot to visualize both FC and significance for each metabolite compared between hepatocytes with PKLR overexpression and corresponded NC, red dots represented significantly higher metabolites (FC > 1.6, *P* < 0.05) in the group with overexpressed PKLR, green dots represented significantly lower metabolites (FC < 0.625, *P* < 0.05) in the group with overexpressed PKLR. (j) Volcano plot to visualize both FC and significance for each metabolite compared between hepatocytes with PKLR downregulation and corresponded NC, red dots represented significantly higher metabolites (FC > 1.6, *P* < 0.05) in the group with downregulated PKLR, and green dots represented significantly lower metabolites (FC < 0.625, *P* < 0.05) in the group with downregulated PKLR. (k) Correlation heatmap for the top 20 metabolites that are associated with PKLR overexpression; the table is color coded by correlation according to the color legend; legend on intensity and direction of correlations is indicated on the right side of the heatmap. (l) Correlation heatmap for the top 20 metabolites that are associated with PKLR downregulation; meaning of legend was the same as (j). (m) Correlation heatmap for the top 20 metabolites that are both associated with PKLR overexpression/downregulation; meaning of legend was the same as (j). (n) Metabolites showed to have association with PKLR overexpression/downregulation by KEGG ID. (o) Overlapped metabolites between PKLR OV and SI groups. (p) Pathway analysis from nontargeted metabolomics based on positive metabolites that are associated with PKLR expression. (q) Details of pathway on glycerophospholipid metabolism and positive metabolites associated with PKLR expression. (r) Details of pathway on linoleic acid metabolism and positive metabolites associated with PKLR expression. Abbreviations: FC: fold change; NC: negative control; OV: overexpression; PCA: principal component analysis; SI: silence.

The whole metabolite profile of HepG2 cells was assayed by nontargeted metabolomic analysis. As shown in Figure 1, PCA by OPLS-DA plot revealed that the metabolomic profile could be clearly separated based on PKLR expression (Figures 1(a) and 1(c)). This was validated by permutation analysis (*R*^2^ = 0.75, *Q*^2^ = −0.37 for the OV group, *R*^2^ = 0.77, *Q*^2^ = −0.52 for the SI group, Figures 1(b) and 1(d)). After adjustment by quality control (QC) samples, 960 and 954 metabolites were matched from the Human Metabolome Database (HMDB) in the OV and SI groups, respectively.

Up-/downregulation of PKLR had no significant effect on the metabolomic perspective (*P* > 0.05, Figure 1(e)). Normalized quantities of identified differential metabolites in the OV and SI groups and their intersection were clustered and presented in heatmaps (Figures 1(j) – 1(l)). Overall, the changes in potential metabolites in cells with altered PKLR expression differed from those in the NC samples. We also demonstrated the significance (fold change/*P* value) for each metabolite in a volcano plot. Notably, prominent elevations (FC > 2, *P* < 0.05) were observed in fewer metabolites in cells with PKLR downregulation (16 vs. 72 between metabolites with increased and decreased levels, Figure 1(n)). Further correlation analysis found that about half of the positive connections were presented among the top 20 metabolites (*P* < 0.05, 51.6%, 48.9%, and 50% for OV, SI, and overlapped components, respectively, Figures 1(o) – 1(q)).

When categorized according to the KEGG database, the Venn diagram found 12 metabolites that overlapped as key molecules in the OV and SI groups (Figure 1(f), Table S5). Pathway analysis revealed that the overlapped metabolites between the OV and SI groups were involved in glycerophospholipid metabolism (*P* < 0.05, impact value = 0.11, Figures 1(g) and 1(h)), with five metabolites having consistent/reverse directions with PKLR variation (Figure 1(r)).

### 3.4. Targeted Metabolomic Analysis of Energy Metabolism in HepG2 Cells

**Figure 2:**
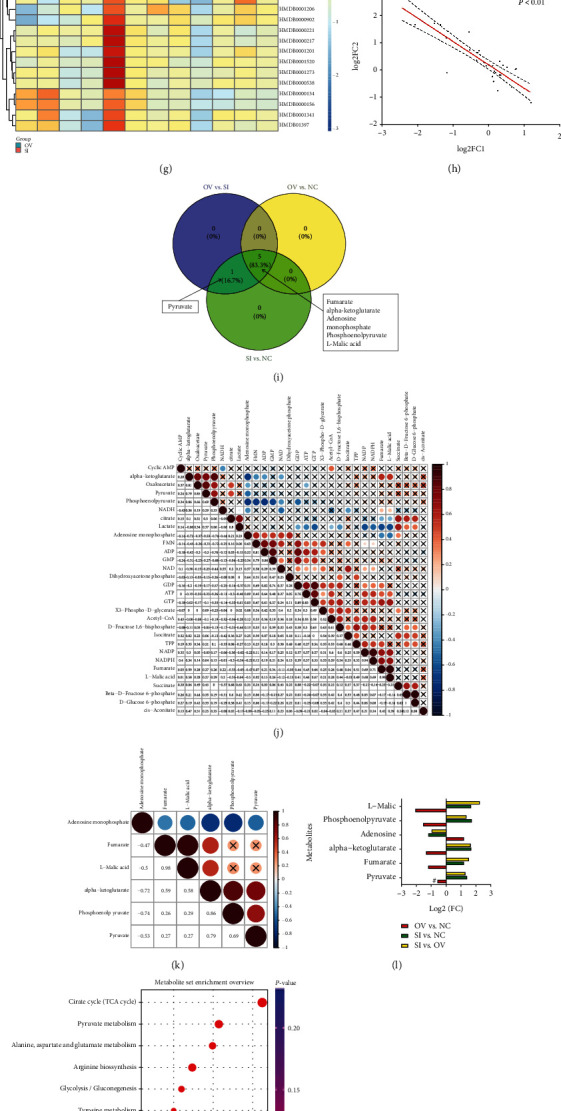
Comprehensive analysis on targeted metabolomic results in cells with PKLR variations. (a) PCA revealed clear separation in targeted metabolomic data from hepatocytes with overexpressed PKLR (OV), dots in green [2] represented the samples with overexpressed PKLR, and dots in blue [1] represented the corresponded NC samples. (b) Validation of OPLS-DA model by class permutation analysis for (a). (c) PCA revealed clear separation in targeted metabolomic data from hepatocytes with downregulated PKLR (SI), dots in green [2] represented the samples with downregulated PKLR, and dots in blue [1] represented the corresponded NC samples. (d) Validation of OPLS-DA model by class permutation analysis for (c). (e) Heatmap for comparison between samples from OV group and corresponded NC in all enrolled metabolites. (f) Heatmap for comparison between samples from SI group and corresponded NC in all enrolled metabolites. (g) Heatmap for comparison between samples from OV and SI groups in all enrolled metabolites. (h) Correlations on log-transformed FCs of each metabolite from OV and SI groups. (i) Overlapped positive metabolites compared in (f)/(g)/(h). (j) Correlation heatmap for all enrolled metabolites from targeted metabolomics; the table is color coded by correlation according to the color legend; legend on intensity and direction of correlations is indicated on the right side of the heatmap. (k) Correlation heatmap for positive metabolites from targeted metabolomics that are associated with PKLR variations; the table is color coded by correlation according to the color legend; legend on intensity and direction of correlations is indicated on the right side of the heatmap. (l) Details on variations of positive metabolites in different comparisons categorized by PKLR expression (OV vs. NC/SI vs. NC/OV vs. SI); # represented insignificant FC in comparison. (m) Rank of pathways based on positive metabolites from targeted metabolomics by enrichment ratios. (n) Pathway analysis based on positive metabolites from targeted metabolomics that are associated with PKLR expression. (o) Details of pathway on TCA cycle and positive metabolites that are associated with PKLR expression. (p) Details of pathway on pyruvate metabolism and positive metabolites that are associated with PKLR expression. (q) Venn plot for those overlapped across the positive metabolites from pathways A (TCA cycle), B (pyruvate metabolism), and C (glycolysis). (r) PEP-to-pyruvate ratio presented in different comparisons (OV vs. NC/SI vs. NC). (s) ATP-to-ADP ratio presented in different comparisons (OV vs. NC/SI vs. NC). (t) NADPH-to-NADP+ ratio presented in different comparisons (OV vs. NC/SI vs. NC). Abbreviations: FC: fold change; NC: negative control; OV: overexpression; PCA: principal component analysis; PEP: phosphoenolpyruvate; SI: silence; TCA: tricarboxylic acid cycle.

Targeted metabolomic analysis of molecules involved in energy metabolism (Table S3) was carried out using a kit developed by Shanghai Applied Protein Technology Company. As shown in Figure 2, PCA revealed that samples could be clearly separated according to PKLR expression in the OPLS-DA model (Figures 2(a) and 2(c)), and the significance of the PCA results was validated by permutation tests (*R*^2^ = 0.72, *Q*^2^ = −1.92 for the OV group, *R*^2^ = 0.76, *Q*^2^ = −3.45 for the SI group, Figures 2(b) and 2(d)).

All the enrolled metabolites were presented in a clustered heatmap (Figures 2(e) – 2(g)). There was a significant inverse correlation across metabolic changes in the OV and SI groups for the whole metabolite profile (*R*^2^ = 0.76, *P* < 0.01, Figure 2(l)). The correlation heatmap showed a percentage of positive links (*P* < 0.05) of only 22.1% for all metabolites (Figure 2(h)).

Six molecules (fumarate, alpha-ketoglutarate, AMP, PEP, L-malic acid, and pyruvate) showed significant associations with PKLR expression in hepatocytes. The FCs of the candidate components are presented in Figure 2(p). Positive interconnections were observed in 84% of links among the candidate metabolites. AMP was inversely correlated with the other five components (Figure 2(i)).

We performed enrichment analysis of the candidate metabolites (Figure 2(j)) and showed that the TCA cycle and pyruvate metabolism were the most involved pathways for the candidate metabolites (*P* < 0.001, impact > 0.15, Figure 2(n), Table S6). Pyruvate and PEP were overlapped in referred TCA cycle, pyruvate metabolism, and glycolysis pathways (Figure 2(q)). Further assessment of the dynamic composite index of the energy metabolites showed significant increments in the pyruvate/PEP and ATP/ADP ratios in cells with PKLR upregulation (*P* < 0.05, Figures 2(m) and 2(n)).

### 3.5. Transcriptomic Data and Integrative Pathway Analysis Based on Multiomic Datasets Categorized by PKLR Expression

**Figure 3:**
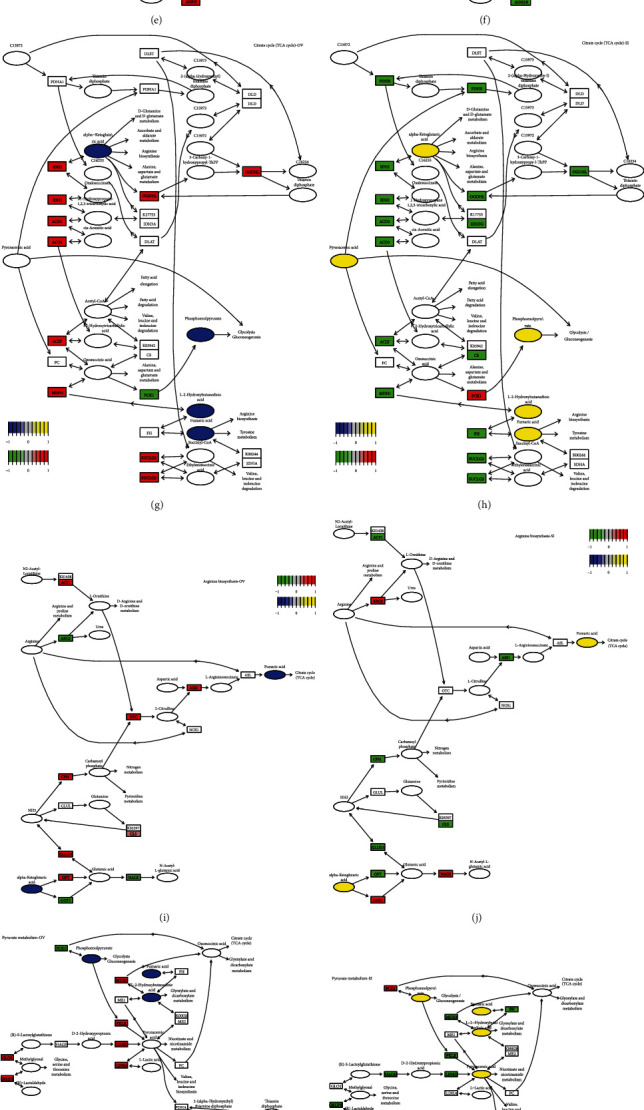
Integrative multiomic study in hepatocytes with PKLR perturbation. (a) Integrative transcriptomic and nontargeted metabolomic analysis identified the variations on pathways of linoleic acid metabolism in hepatocytes with overexpressed PKLR. (b) Integrative transcriptomic and nontargeted metabolomic analysis identified the variations on pathways of linoleic acid metabolism in hepatocytes with downregulated PKLR. (c) Integrative transcriptomic and nontargeted metabolomic analysis identified the variations on pathways of glycerophospholipid metabolism in hepatocytes with overexpressed PKLR. (d) Integrative transcriptomic and nontargeted metabolomic analysis identified the variations on pathways of glycerophospholipid metabolism in hepatocytes with downregulated PKLR. (e) Integrative transcriptomic and targeted metabolomic analysis identified the variations on pathways of glycolysis in hepatocytes with overexpressed PKLR. (f) Integrative transcriptomic and targeted metabolomic analysis identified the variations on pathways of glycolysis in hepatocytes with downregulated PKLR. (g) Integrative transcriptomic and targeted metabolomic analysis identified the variations on pathways of citrate cycle in hepatocytes with overexpressed PKLR. (h) Integrative transcriptomic and targeted metabolomic analysis identified the variations on pathways of citrate cycle in hepatocytes with downregulated PKLR. (i) Integrative transcriptomic and targeted metabolomic analysis identified the variations on pathways of arginine biosynthesis in hepatocytes with overexpressed PKLR. (j) Integrative transcriptomic and targeted metabolomic analysis identified the variations on pathways of arginine biosynthesis in hepatocytes with downregulated PKLR. (k) Integrative transcriptomic and targeted metabolomic analysis identified the variations on pathways of pyruvate metabolism in hepatocytes with overexpressed PKLR. (l) Integrative transcriptomic and targeted metabolomic analysis identified the variations on pathways of pyruvate metabolism in hepatocytes with downregulated PKLR. (m) Integrative transcriptomic and targeted metabolomic analysis identified the variations on pathways of butanoate metabolism in hepatocytes with overexpressed PKLR. (n) Integrative transcriptomic and targeted metabolomic analysis identified the variations on pathways of butanoate metabolism in hepatocytes with downregulated PKLR. (o) Network connection for variations of key metabolites from targeted metabolomics in hepatocytes with overexpressed PKLR. In (a–n), the frame in rectangle represented the genes involved in pathways; the frame in ellipse or circle represented the metabolites involved in pathways. In (o), the frame in green represented downregulation, and the frame in red represented upregulation in cells with PKLR overexpression.

Based on DEGs from RNA-seq data, GSEA identified 10 and 15 KEGG pathways that were significantly associated with PKLR expression, respectively (*P* < 0.05). Following PKLR alterations, pathways relating to metabolism, glycine/serine/threonine metabolism, peroxisome proliferator-activated receptor signaling, and butanoate and neuroactive ligand-receptor interaction were shown to be overlapped candidates with consistent trends in both the OV and SI groups (Table S7, Figures S3 and S4, and Figure 3).

Molecular pathways were imputed and visualized using online tools (MetaboAnalyst and Pathview) [29, 32], based on the integration of differential metabolites and genes related to PKLR variations in omics data from HepG2 cells.

Pathway imputation was first performed by integrative evaluation of clustered significant metabolites and genes from omics data in the OV and SI groups, respectively, via the online toolkit MetaboAnalyst (https://www.metaboanalyst.ca/; Tables S8–S11), and the imputed pathways were then merged in accordance with the datasets from the OV and SI groups (Tables S12 and S13). For nontargeted metabolomic data, glycerophospholipid and linoleic acid metabolism were significant pathways including metabolites in both the OV and SI groups. Notably, C00157 (glycerophospholipid) was involved as an overlapped potential metabolite in selected pathways based on data from both the OV and SI groups (Figures 3(a) – 3(d), Table S12).

Similarly, PKLR expression was significantly related to five pathways, including glycolysis, pyruvate metabolism, the TCA cycle, arginine biosynthesis, and butanoate metabolism, with the inclusion of candidate metabolites identified by multiomic analysis in both the OV and SI groups for targeted metabolomic data (Figures 3(e) – 3(n), Table S13).

All metabolites involved in glycolysis and pyruvate metabolism were included in the TCA process. Intriguingly, AMP, as the only molecule affected in the opposite direction to the other metabolites, was not involved in any of the integrated pathways. Further clustering analysis showed that the supporting metabolites in arginine biosynthesis differed from the compounds involved in the TCA cycle, glycolysis, and pyruvate metabolism, while alpha-ketoglutarate played a key role in linking the two different pathway clusters (Figure 3(o)). Finally, the selected pathways were visualized using Pathview (https://pathview.uncc.edu/). Details of the key pathways are shown in Figure 3.

### 3.6. Metabolomic Validation in LT Grafts

**Figure 4:**
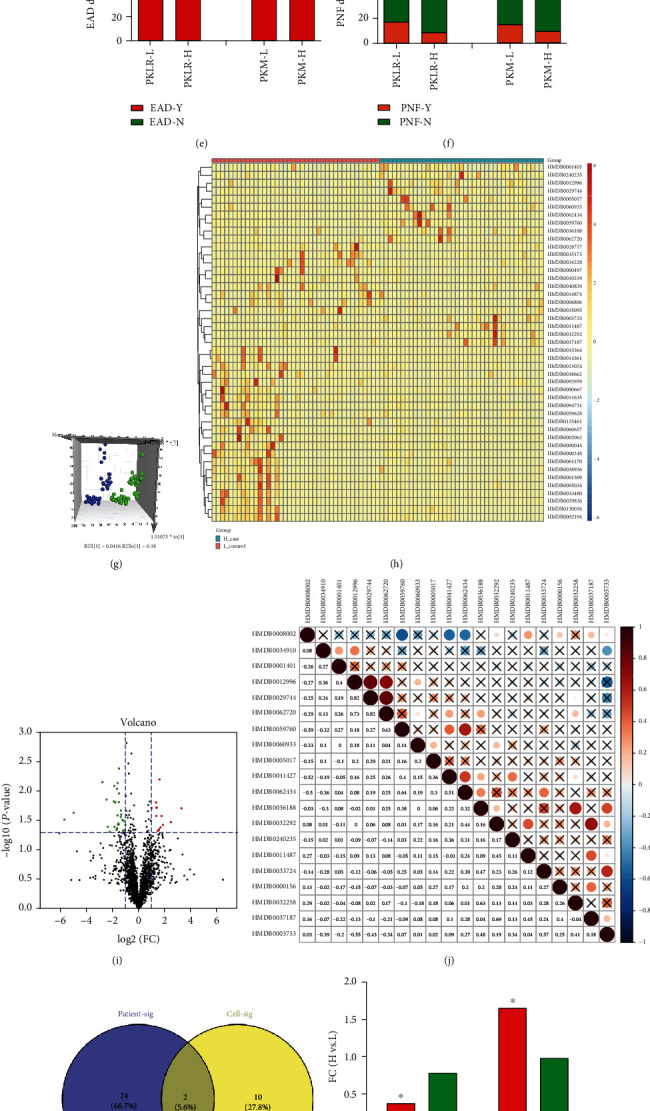
Comprehensive analysis on nontargeted metabolomic results in grafts for transplantation classified by PK variations. (a) Heatmap for correlation between interconnection between PKLR/PKM genes and clinical factors in LT; frame in ∗ represented the correlation with statistical significance (*P* < 0.05). (b) Correlation analysis between log-transformed PKLR and PKM expression in grafts for LT. (c) Analysis on grafts' survival categorized by PKLR/PKM expression. (d) Analysis on patients' survival categorized by PKLR/PKM expression. (e) Distribution of EAD occurrence in patients categorized by PKLR/PKM expression. (f) Distribution of PNF occurrence in patients categorized by PKLR/PKM expression. (g) PCA in patients categorized by PKLR expression, dots in blue represented the samples with higher PKLR, and the dots in green represented the samples with lower PKLR. (h) Heatmap for clusters of metabolites significantly associated with PKLR expression. (i) Volcano plot on visualization of both FC and significance for each metabolite compared between higher and lower PKLR expression, red dots represented significantly higher metabolites (FC > 2, *P* < 0.05) in grafts with higher PKLR, and green dots represented significantly lower metabolites (FC < 0.5, *P* < 0.05) in grafts with higher PKLR. (j) Correlation heatmap for the top 20 metabolites associated with PKLR expression. (k) Overlap between significant metabolites in cells and grafts by KEGG identification. (l) FC of C00157 and C04230 categorized by PKM/PKLR expression. (m) Scatter plot for correlation between log-transformed PKLR and C00157 expression. (n) Scatter plot for correlation between log-transformed PKLR and C04230 expression. (o) Scatter plot for correlation between log-transformed PKM and C00157 expression. (p) Scatter plot for correlation between log-transformed PKM and C04230 expression. Abbreviations: EAD: early allograft dysfunction; FC: fold change; LT: liver transplantation; PCA: principal component analysis; PNF: primary nonfunction.

The genetic impacts of PKLR on clinical and metabolomic profiles of LT grafts are shown in Figure 4. Perioperative features, including blood loss/transfusion, surgical duration, and indicators of coagulation/liver function, showed close mutual connections with each other (*P* < 0.05, Figure 4(a)). Details of the correlations between the genetic and clinical indicators are presented in Figure S5. There was a weak association between PKLR and PKM, as another PK-encoding gene in the liver (*P* > 0.05, Figure 4(b)). Survival analysis also showed that PKLR (but not PKM) negatively affected posttransplant prognosis (HR for PKLR: 0.39 (95% CI: 0.22-0.71), *P* < 0.05; HR for PKM: 1.05 (95% CI: 0.59-1.86), *P* > 0.05; Figures 4(c) and 4(d)), and PKLR was negatively associated with the occurrence of EAD (51% vs. 75% for higher vs. lower PKLR expression, Figure 4(e)).

The hazard ratios (HRs) of higher PKLR expression for posttransplant survival of patients and grafts (PS/GS) were 0.37 (95% confidence interval (CI): 0.24–0.64) and 0.49 (95% CI: 0.32–0.81), respectively, while the risk was decreased in patients with grafts with higher PKLR expression (PS/GS HR = 0.37/0.49, Table S14). Further metabolomic analysis was performed in grafts categorized by median PKLR expression. PCA using the OPLS-DA model showed clear separation across groups with higher and lower PKLR expression (Figure 4(g)).

Among the 2513 metabolites identified by HMDB IDs, 45 differential metabolites (14 upregulated vs. 31 downregulated) had significant associations with PKLR expression in the volcano diagram (∣log_2_FC | >1, *P* < 0.05, Figure 4(i)). Potential metabolites seemed disperse and independent with less intercorrelation (21.5% of positive links in top significant metabolites, Figures 4(h) and 4(j)). According to KEGG identity, PC (C00157) and 2-lysolecithin (C04230) were significantly overlapping components in both cells and LT grafts according to PKLR expression (Figure 4(k)). Higher PKLR (but not PKM) expression was associated with decreased C04230 and increased C00157 (Figure 4(l), *P* < 0.05). These trends were confirmed in scatter plots (*P* < 0.05, Figures 4(m) – 4(p)).

### 3.7. Weighted Metabolome Coexpression Network Analysis of LT Grafts

**Figure 5:**
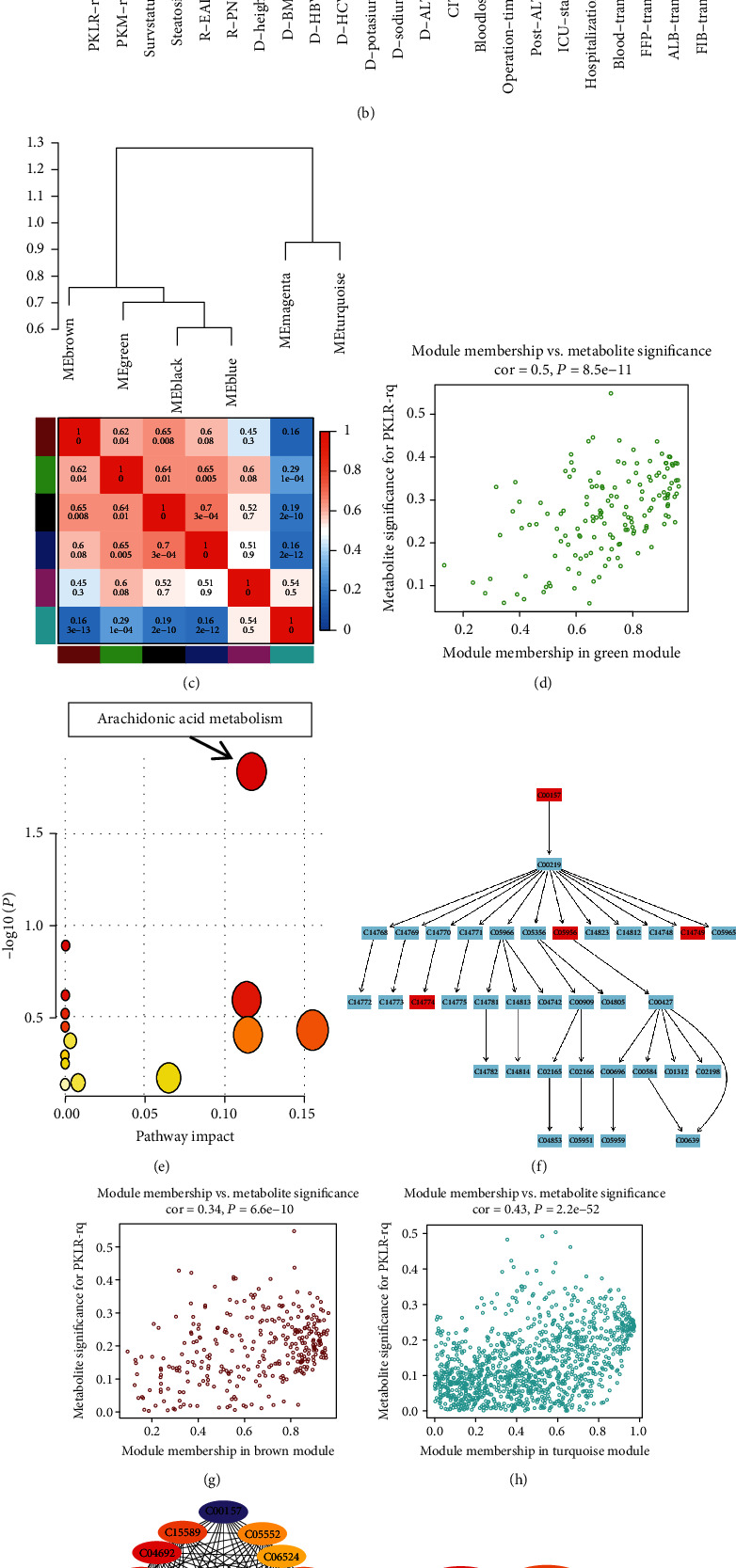
Weighted correlation network analysis on nontargeted metabolome of grafts and its connections with clinical factors. (a) Cluster dendrogram obtained by dissimilarity based on consensus topological overlap with the corresponding modules indicated by the color row. Each colored row represented a color-coded module containing a group of highly connected metabolites. (b) Relationships of consensus module and clinical features of LT cases. Each row in the table corresponded to a consensus module, and each column corresponded to a feature. The module name was shown on the left side for each cell. Numbers in the table reported the correlations of the corresponded module and feature, with the *P* values printed below. The table is color coded by correlation according to the color legend. Intensity and direction of correlations are indicated on the right side of the heatmap. (c) Dendrogram of consensus module and heatmap of the adjacencies obtained by WGCNA on the consensus correlation. Numbers in the table reported the intermodule correlations, with the *P* values printed below. The table was color coded by correlation according to the color legend indicated on the right side of the heatmap. (d) Scatterplot of metabolite significance PKLR vs. MM in the green module. (e) Results for pathway enrichment based on metabolites in the green module. (f) Details of arachidonic acid metabolism and related metabolites involved in module. (g) Scatterplot of metabolite significance PKLR vs. MM in the brown module. (h) Scatterplot of metabolite significance PKLR vs. MM in the turquoise module. (i) Coexpression network by top 20 nodes based on coexpressed links in positive metabolites from nontargeted metabolomics of hepatocytes. (j) Coexpression network by top 10 nodes based on coexpressed links in positive metabolites from nontargeted metabolomics of human grafts for transplantation. (k) Coexpression network centered by C00157 and C04230 based on coexpressed links from nontargeted metabolomics of hepatocytes. (l) Coexpression network centered by C00157 and C04230 based on coexpressed links from nontargeted metabolomics of grafts for transplantation. Abbreviations: LT: liver transplantation; MM: module membership.

We constructed a network model of the metabolome for LT grafts by WGCNA, as a scale-free model. Seven modules were detected based on predefined cut-off values of 0.8 (Figure 5(a)). Connectivity across eigengenes was checked by cluster analysis. The brown, green, black, and blue modules were differentiated but showed close interactions with a relatively high correlation index (*r* > 0.6, Figure 5(c)).

We also assessed the relationships between the metabolite modules and clinical indicators (Figure 5(b)). The brown, black, and blue modules were significantly associated with blood product transfusion and subsequent occurrence of EAD, with increased metabolites in the turquoise module indicating more transfusion of blood products and more severe EAD. Notably, there was an inverse correlation between PKLR expression and EAD occurrence in the green module, which was independent of the transfusion of blood products. The scatter plot showed significant correlations between module membership and metabolite significance based on all enrolled molecules in the green, brown, and turquoise modules (*r* = 0.50, 0.34, and 0.43, respectively, *P* < 0.05). Further analysis also showed that the metabolites included in the green module were enriched in the arachidonic acid (ARA) metabolism pathway (Figures 5(e) and 5(f)).

### 3.8. Screening of Hub Metabolites Correlated with PKLR Expression

Further networks of potential candidate metabolites with differential expression in cells and graft tissues according to PKLR variations were visualized using Cytoscape software [30].

For nontargeted metabolomic data, 2216 significantly coexpressed links between positive metabolites in the OV and SI groups in hepatocytes were imputed by correlation tests (*P* for correlation < 0.05, Table S15). A coexpression network was constructed using the MNC algorithm for the top 20 nodes, including C00157 and C04230 (Figure 5(j)). We also constructed a coexpression network using the MCC algorithm for the top metabolites based on 139 positive links in LT grafts (Table S16). Consistent with the data for cells, the key roles of C00157 and C04230 were validated in tissues.

There was an inverse correlation between C00157 and C04230 in liver cells and liver tissues. Furthermore, we also created coexpression models for metabolites centered on C00157 and C04230 as overlapped molecules in cells and liver tissues (Figures 5(k) and 5(m)). Around 34% of metabolites were associated with the variations in C00157 and C04230, simultaneously (Table S17), and these cocorrelations were more common in metabolites (80%) from transplanted grafts (Table S18).

### 3.9. Cellular Lipogenesis Associated with PKLR Expression

**Figure 6:**
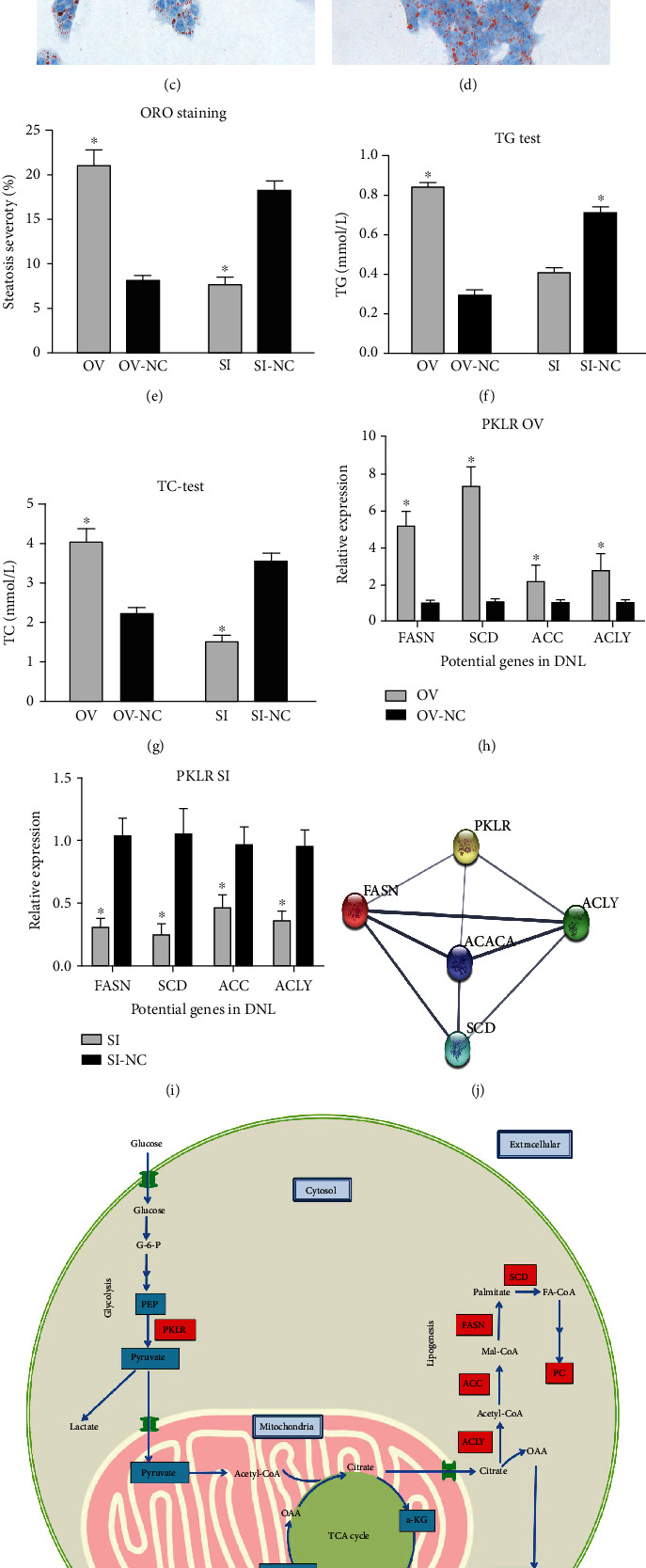
Genetic impact of pyruvate kinase L/R on the process of de novo lipogenesis. HepG2 cells with PKLR alteration were treated by high-glucose medium (50 mmol/L), respectively, for 48 hours. And lipogenic severity was evaluated by ORO staining. (a) ORO staining for cells with overexpressed PKLR. (b) ORO staining for NC cells with overexpressed PKLR. (c) ORO staining for cells with suppressed PKLR. (d) ORO staining for NC cells with suppressed PKLR. (e) Comparison of lipogenic severity between cells with overexpressed/suppressed PKLR and their corresponded NC. (f) Comparison of hepatic TG between cells with overexpressed/suppressed PKLR and their corresponded NC. (g) Comparison of hepatic TC between cells with overexpressed/suppressed PKLR and their corresponded NC. (h) Comparison of key genes that located on DNL process between cells with overexpressed PKLR and their corresponded NC. (i) Comparison of key genes that located on DNL process between cells with suppressed PKLR and their corresponded NC. (j) PPI network between PKLR and selected genes that located on DNL process. (k) Speculated mechanism for the impact of PKLR on DNL process. Stained cells were observed and scanned under a microscope (magnification: 400x); ∗ represented statistical significance for comparisons between targeted cells and corresponded NC (*P* < 0.05); TG and TC were both evaluated in systems with 100 000 cells in 100 *μ*L of solution buffer. Frames in red represented the molecules with upregulations; frames in blue represented the molecules with downregulations. Abbreviations: a-KG: alpha-ketoglutarate; DNL: de novo lipogenesis; G-6-P: glucose-6-phosphate; ORO: oil red O; NC: negative control; OAA: oxaloacetate; PEP: phosphoenolpyruvate; PC: phosphatidylinositol; PKLR: pyruvate kinase L/R; PPI: protein-protein interaction; TCA: tricarboxylic acid cycle; TC: total cholesterol; TG: triglyceride.

HepG2 cells with different PKLR expression levels were incubated in high-glucose medium. And high-glucose levels resulted in severer steatosis and higher cellular TG and TC levels in cells overexpressing PKLR (*P* < 0.05, Figure 6). Similarly, decreased fat accumulation with lower TG and TC levels was also presented in cells with suppressed PKLR (*P* < 0.05).

Combined with results from metabolomic and transcriptomic data, we imputed and described the potential role of PKLR in the DNL process.

Meanwhile, key genes with significance in transcriptomic data on the DNL process were also validated to be associated with PKLR perturbation by the qRT-PCR test. Further PPI network analysis also revealed a close connection between PKLR and most selected genes on the DNL process (Figure 6).

## 4. Discussion

### 4.1. Omics Study in HepG2 Cells

We previously found a positive correlation between PKLR expression and HepG2 cell viability [8]; however, the role of metabolites in this connection was unclear and worthy of further investigation. In the current study, we systemically evaluated the biological function and clinical implications of PKLR by combined transcriptomic and metabolomic analysis of liver cells and tissues.

Glycerophospholipid and linoleic acid metabolism were identified as pathways affected by changes in PKLR expression in hepatic cells according to global transcriptome and metabolome analysis. The glycerophospholipid molecule (C00157) was also positively correlated with PKLR expression. Targeted study of energy metabolism also found that differential metabolites were enriched in TCA and pyruvate metabolism pathways. Diminished reaction was observed on candidate metabolic pathways in cells with overexpressed PKLR. Integrative analysis of RNA-seq and metabolomic data also suggested that the PKLR mechanism could be extended to involve the glycolysis, arginine biosynthesis, and butanoate metabolism pathways, while alpha-ketoglutarate exerted a key effect in regulating the coexpression network associated with PKLR modulation by candidate energy metabolites.

### 4.2. LT Grafts and Liver Cell Line Results

Regarding LT grafts, donor PKLR expression had a protective effect in terms of posttransplant prognosis and was associated with lower incidences of EAD, graft failure, and patient death. Further network analysis by WGCNA found that the effect of graft PKLR expression was independent of the effects of surgical factors, such as transfusion of blood products on metabolomic perspectives. Metabolites in the PKLR-related module (green) were enriched in ARA metabolism, and PC (C00157) and 2-lysolecithin (C04230) were presented as hub metabolites related to PKLR expression in both liver cells and liver tissues.

### 4.3. Impact of PKLR on Glycerophospholipid and Linoleic Acid Metabolism

We previously found that hepatic cell growth was accelerated in cells overexpressing PKLR [8]. PC is a major component of biological membranes [33], and PC synthesis is required for cell proliferation and differentiation [34]. As an autotaxin of PC, increased lyso-PC also affects the progression of the cell cycle [35]. Our combined transcriptomic and metabolomic analysis accordingly suggested that the positive impact of PKLR on cell growth might be due to its upregulation of glycerophospholipid metabolism, specifically by increasing PC (C00157) and its deacylated derivative 2-lyso-PC (C04230). This extensive study of PKLR function suggested that the causal link between PKLR and accelerated cell growth might involve the generation of lipids required for the creation of cell membranes.

### 4.4. Impact of PKLR on Energy Metabolism by Targeted Metabolomic Analysis

Given the crucial role of PKLR in regulating energy metabolism, we carried out targeted metabolomic analysis in cells with altered PKLR expression. Unsurprisingly, PK was activated in the presence of an increased pyruvate/PEP ratio in cells overexpressing PKLR [36]. Meanwhile, a higher ATP/ADP ratio indicated improved aerobic mitochondrial function and oxidative phosphorylation in cells with upregulated PKLR [37]. Enhanced flux of anabolic and synthetic metabolism seemed to be the main cellular feature affected by PKLR. The TCA cycle and pyruvate metabolism pathways were identified as the most likely pathways affected by PKLR expression in hepatoma cells. As substrates in glycolysis catalyzed by PK, PEP and pyruvate are both involved in potential pathways related to PKLR. Interestingly, network analysis revealed that alpha-ketoglutarate acted as a hub metabolite in the cluster of potential candidates coexpressed with PKLR. As a precursor of glutamate and glutamine [38], alpha-ketoglutarate is a key molecule determining the rate of the canonical TCA cycle [39]. PKLR might thus determine cell fate by regulating the production of the metabolic fuel supply centered on alpha-ketoglutarate.

### 4.5. Imputative Mechanism for Impact of PKLR on Cellular Metabolism

The results of metabolomic and transcriptomic analyses can be related to de novo lipogenesis (DNL), and PKLR might participate in activating the DNL process in hepatic cells. DNL represents the process involving the conversion of carbohydrate in the circulation to the formation of lipids in the liver [40]. The current results of lipogenic assays in hepatic cells confirmed that PKLR might be involved in DNL activation under high-glucose-induced oxidative stress (Figure 6).

Meanwhile, the generation of PC, as a major cell membrane component and an end product of DNL, provides a substrate for further cell growth and proliferation. The efficiency of DNL depends on the TCA cycle [41, 42]. We previously found that PKLR was coexpressed with Fatty Acid Synthase (FASN) as the key gene of DNL in the liver [7], and the current integrative multiomic analysis confirmed that PKLR per se was also involved in DNL. And this speculation was also confirmed by further lipogenic assays (Figure 6). PKLR exerted its positive effects by upregulating the synthesis of PC at the expense of exhausting the consumption of intermediates in the TCA cycle in mitochondria. The potential impact of PKLR on the DNL mechanism is shown in Figure 6(f).

### 4.6. WGCNA of LT Grafts

We constructed a network model for the impact of PKLR on graft metabolism and survival in LT cases. PKLR showed protective effects on posttransplant outcomes. Clinically, intraoperative bleeding and transfusion of blood products had significant impacts on graft survival. Coexpression network analysis also showed that the functional module (green color) for PKLR was distinct from modules for operational risk traits. Based on a metabolomic perspective, our data indicated that PKLR might have an independent impact on posttransplant prognosis, irrespective of surgical risk covariates.

Pathway analysis showed that metabolites in the PKLR-related module were enriched in ARA metabolism, with molecules including prostaglandin G2 being activated in grafts overexpressing PKLR. As a polyunsaturated fatty acid component of membrane phospholipids, ARA plays vital roles as substrates for cell survival, growth, and proliferation [43, 44]. ARA might also alleviate the inflammatory response via complex regulation of relevant cytokines [45], and activation of ARA metabolism has demonstrated anti-inflammatory effects of PKLR in the liver, as a more complex system.

Interestingly, PKLR expression overlapped with PC (C00157) in a consistent direction, indicating that PKLR promoted the PC content in both cells and graft tissues. However, the networks centered on the overlapped metabolites (C00157 and C04230) differed between cells and LT grafts. The comprehensive effects of PC on graft function have been summarized previously [46]. The current results suggested that PKLR might improve graft quality and postoperative survival by positively regulating PC, as a major component of the cell membrane. Further studies are needed to clarify the interconnections among PKLR expression, liver inflammation, and cell survival.

### 4.7. Genetic Functions of PKM and PKLR

PK occurs as four isozymes with different organ specificities and encoded by different genes (PKM/PKLR), with the liver expressing both genes [3]. A recent study reviewed the multifaceted function of the PKM gene in supporting cell growth and carcinogenesis and also noted its nonspecific occurrence throughout the human body [47]. In contrast, we previously showed that PKLR was a possible liver-specific target for disease therapy, with PKLR inhibition potentially having more benefits and fewer complications for the treatment of liver cancer [7]. The current study suggested that PKLR might exert its unique effects on liver metabolomic independent of PKM2. However, further studies are needed to investigate the distinct properties of PK isozymes.

### 4.8. Strength of the Study in terms of Clinical Applications

The current study had several strengths. First, the mechanistic pathways of PKLR were derived from integrative analysis based on transcriptomic and metabolomic data, thus providing mutual confirmation of the results. Second, the results of targeted and nontargeted metabolomic data outlined the function of PKLR in the DNL process, from carbohydrate utilization (TCA) to PC composition. Third, metabolomic data from hepatocytes and liver tissues confirmed the reliability of the potential metabolites by cross-validation at different levels. Finally, the network model was constructed by comprehensive adjustment of surgical indicators to clarify the intrinsic genetic function following artificial interventions.

### 4.9. Potential Limitations and Future Directions

Some potential limitations of this study should also be noted. First, as a comprehensive analysis based on the whole molecule profile, the multiomic data identified the most important candidate pathways; however, functional experiments are needed to validate the details of the intermolecular connections. Second, WGCNA cannot be used in integrated omics data from hepatic cell lines for relatively fewer samples and phenotypic traits acquired. Third, some algorithms like Mendelian Randomization (MR) might be useful for the elucidation of causal links across transcriptomic and proteomic data by PKLR perturbations [48–50]. However, the lack of proteomic data limited the further use of MR on clarification of these causalities. Fourth, the genetic function of PKLR cannot be assessed in grafts after implantation for ethical reasons, and further *in vivo* analysis using LT models (e.g., in rats or mice) could help to elucidate the potential mechanisms. The results of the current study revealed that PKLR might affect hepatoma cell proliferation via its impact on lipogenesis, but further *in vitro* and *in vivo* experiments are needed to clarify the regulatory mechanism of PKLR on the abovementioned pathways. Otherwise, transcriptional translation is complex, and the role of N4-acetylcytidine (ac4C) as an important mRNA regulator on the exertion of genetic effect of PKLR is also worthy of further investigations [51].

### 4.10. Conclusion

In conclusion, this comprehensive integrative transcriptomic and metabolomic study showed that PKLR might promote cell growth and proliferation by regulating DNL. PC and lyso-PC played central roles as key mediators in pathways affected by PKLR in both hepatoma cells and LT grafts. PKLR may thus promote graft survival via activating ARA metabolism and relieving inflammation. Contrastingly, PKLR might accelerate the progression of tumor cell growth but might also improve the survival of grafts after LT. The effects of altered PKLR expression on the metabolomic profiles of hepatoma cells and grafts suggest the need to assess its function in specific scenarios.

## Figures and Tables

**Table 1:** Clinical features of liver transplant cases.

Covariates	Characteristics
Recipients (R)	
R-age (years)	47.7 ± 12.1
R-gender (M/F)	67/12
R-BMI (kg/m^2^)	23.5 ± 3.1
R-HBV infector (Y/N)	62/17
R-blood type (A/B/O/AB)	33/8/33/5
R-MELD score	33 (27-40)
R-Child-Pugh score	11 (10-12)
Indication for LT	
(Viral hepatitis-related cirrhosis/cholestatic cirrhosis/liver failure/liver cancer/others)	24/5/12/13
Donors (D)	
D-age (years)	41.7 ± 13.7
D-gender (M/F)	67/12
D-BMI (kg/m^2^)	23.2 ± 2.7
D-HBV infector (Y/N)	11/68
D-blood type (A/B/O/AB)	27/10/31/11
D-ALT (U/L)	40.0 (25.0-66.0)
D-TB (*μ*mol/L)	17.2 (11.0-23.1)
D-CR (*μ*mol/L)	87.0 (56.3-151.6)
D-BUN (mmol/L)	8.4 (5.2-11.0)
D-sodium (mmol/L)	146 (138-152)
D-potassium (mmol/L)	3.8 (3.7-4.3)
Grafts (G)	
PKLR-RQ	1.0 (0.3-9.4)
PKM-RQ	1.0 (0.5-3.0)
Steatosis (MaS/MiS/none)	35/9/35
Donation type (DCD/DBD)	57/22
Surgery	
Surgical duration (min)	303 (272-375)
CIT (min)	652 (567-744)
WIT (min)	7 (1-12)
Blood loss (mL)	1500 (800-2500)
Blood product transfusion	
FFP (mL)	780 (540-1120)
RBC (U)	5 (2-8)
PCC (U)	2000 (900-3000)
ALB (g)	125 (75-150)
FIB (g)	5 (0.5-10)
Posttransplant events	
Peak TB (*μ*mol/L)	211 (125-387)
Peak ALT (U/L)	2571 (1972-3255)
Peak AST (U/L)	6284 (4485-9712)
EAD (Y/N)	50/29
PNF (Y/N)	10/69
Follow-up duration (d)	308 (35-980)

Data in normal distribution was presented by mean ± SD, and data in nonnormal distribution was presented by median (IQR (interquartile range)). Abbreviations: ALB: albumin; ALT: alanine aminotransferase; AST: aspartate aminotransferase; BMI: body mass index; CIT: cold ischemia time; D: donor; DBD: donation after brain death; DCD: donation after cardiac death; EAD: early allograft dysfunction; F: female; FFP: fresh frozen plasma; FIB: fibrinogen; HBV: hepatitis B virus; LT: liver transplantation; M: male; MELD: model for end-stage liver disease; PCC: prothrombin complex; PNF: primary liver graft nonfunction; R: recipient; RBC: red blood cell; RQ: relative quantity; TB: total bilirubin; WIT: warm ischemia time.

## Data Availability

The data used to support the findings of this study are available from the corresponding authors upon request.

## Supplementary Materials

Supplementary Materials Table S1: shRNA sequence for SI of PKLR. Table S2: sequence for primer of target genes. Table S3: information of enrolled metabolites for energy metabolism. Table S4: toolkit used for data visualization. Table S5: details of overlapped metabolites in the OV and SI groups from nontargeted metabolomics in hepatocytes. Table S6: speculative pathways based on enriched energy metabolites that are associated with PKLR variations. Table S7: KEGG pathway enrichment on differential genes from HepG2 cells with PKLR perturbation by GSEA. Table S8: speculative pathways in hepatocytes with overexpressed PKLR by integrative analysis of transcriptomic and nontargeted metabolomic data. Table S9: speculative pathways in hepatocytes with downregulated PKLR by integrative analysis of transcriptomic and nontargeted metabolomic data. Table S10: speculative pathways in hepatocytes with overexpressed PKLR by integrative analysis of transcriptomic and targeted metabolomic data. Table S11: speculative pathways in hepatocytes with downregulated PKLR by integrative analysis of transcriptomic and targeted metabolomic data. Table S12: potential pathways overlapped between transcriptomics and nontargeted metabolomic data in the OV and SI groups. Table S13: potential pathways overlapped between transcriptomics and targeted metabolomic data in the OV and SI groups. Table S14: impact of graft PKM and PKLR expression on posttransplant prognosis. Table S15: positive coexpressed links in hepatocytes with PKLR perturbation. Table S16: positive coexpressed links in grafts for liver transplantation. Table S17: coexpressed links centered by C00157 and C04230 in hepatocytes categorized by PKLR perturbation. Table S18: coexpressed links centered by C00157 and C04230 in grafts categorized by PKLR expression. Figure S1: flow diagram of study design. Figure S2: construction of hepatocytes with PKLR perturbation. Figure S3: KEGG pathway enrichment on differential genes from HepG2 cells with overexpressed PKLR by GSEA. Figure S4: KEGG pathway enrichment on differential genes from HepG2 cells with suppressed PKLR by GSEA. Figure S5: correlation analysis for interconnection in matrix with inclusion of PKLR/PKM gene and all enrolled clinical indicators.
