# Supplementary material for: Integrative Network Analysis Revealed Genetic Impact of Pyruvate Kinase L/R on Hepatocyte Proliferation and Graft Survival after Liver Transplantation
Source: Oxid Med Cell Longev. 2021 Sep 2;2021:7182914. doi: 10.1155/2021/7182914 (PMC8429008; doi:10.1155/2021/7182914)
Supplement: Supplementary Materials — Table S1: shRNA sequence for SI of PKLR. Table S2: sequence for primer of target genes. Table S3: information of enrolled metabolites for energy metabolism. Table S4: toolkit used for data visualization. Table S5: details of overlapped metabolites in the OV and SI groups from nontargeted metabolomics in hepatocytes. Table S6: speculative pathways based on enriched energy metabolites that are associated with PKLR variations. Table S7: KEGG pathway enrichment on differential genes from HepG2 cells with PKLR perturbation by GSEA. Table S8: speculative pathways in hepatocytes with overexpressed PKLR by integrative analysis of transcriptomic and nontargeted metabolomic data. Table S9: speculative pathways in hepatocytes with downregulated PKLR by integrative analysis of transcriptomic and nontargeted metabolomic data. Table S10: speculative pathways in hepatocytes with overexpressed PKLR by integrative analysis of transcriptomic and targeted metabolomic data. Table S11: speculative pathways in hepatocytes with downregulated PKLR by integrative analysis of transcriptomic and targeted metabolomic data. Table S12: potential pathways overlapped between transcriptomics and nontargeted metabolomic data in the OV and SI groups. Table S13: potential pathways overlapped between transcriptomics and targeted metabolomic data in the OV and SI groups. Table S14: impact of graft PKM and PKLR expression on posttransplant prognosis. Table S15: positive coexpressed links in hepatocytes with PKLR perturbation. Table S16: positive coexpressed links in grafts for liver transplantation. Table S17: coexpressed links centered by C00157 and C04230 in hepatocytes categorized by PKLR perturbation. Table S18: coexpressed links centered by C00157 and C04230 in grafts categorized by PKLR expression. Figure S1: flow diagram of study design. Figure S2: construction of hepatocytes with PKLR perturbation. Figure S3: KEGG pathway enrichment on differential genes from HepG2 cells with overexpressed PKLR b [file 7182914.f1.docx]

**Summary of supplementary tables & figures**

Table S1 shRNA sequence for SI of PKLR

| Species | Target | NCBI-ID | Sequence of shRNA |
| --- | --- | --- | --- |
| Homo | PKLR | 5313 | GTCTGTGCCACACAGATGCTGGAGAGCAT |

Table S2 Sequence for primer of target genes

| Species | Target | NCBI-ID | Direction |
| --- | --- | --- | --- |
| Homo | PKLR | 5313 | Forward |
| Homo | PKLR | 5313 | Reverse |
| Homo | PKM | 5315 | Forward |
| Homo | PKM | 5315 | Reverse |
| Homo | B-ACT | 60 | Forward |
| Homo | B-ACT | 60 | Reverse |
|  |  |  |  |

Table S3 Information of enrolled metabolites for energy metabolism

| Component Name | HMDB ID | HMDB |
| --- | --- | --- |
| Succinyl coenzyme A (Succinyl-CoA) | HMDB0001022 | https://hmdb.ca/metabolites/HMDB0001022 |
| NADPH | HMDB0000221 | https://hmdb.ca/metabolites/HMDB0000221 |
| Reduced nicotinamide adenine dinucleotide (NADH) | HMDB01487 | https://hmdb.ca/metabolites/HMDB0001487 |
| Acetyl coenzyme A (Acetyl-CoA) | HMDB01206 | https://hmdb.ca/metabolites/HMDB0001206 |
| 3-Phospho-D-glycerate | HMDB60180 | https://hmdb.ca/metabolites/HMDB0060180 |
| Adenosine 5'-triphosphate (ATP) | HMDB00538 | https://hmdb.ca/metabolites/HMDB0000538 |
| Guanosine 5'-triphosphate (GTP) | HMDB01273 | https://hmdb.ca/metabolites/HMDB0001273 |
| cis-Aconitate | HMDB0000072 | https://hmdb.ca/metabolites/HMDB0000072 |
| Citrate | HMDB00094 | https://hmdb.ca/metabolites/HMDB0000094 |
| Guanosine 5'-diphosphate (GDP) | HMDB01201 | https://hmdb.ca/metabolites/HMDB0001201 |
| Thiamine pyrophosphate (TPP) | HMDB0001372 | https://hmdb.ca/metabolites/HMDB0001372 |
| ADP | HMDB0001341 | https://hmdb.ca/metabolites/HMDB0001341 |
| alpha-ketoglutarate | HMDB0000208 | https://hmdb.ca/metabolites/HMDB0000208 |
| Adenosine monophosphate | HMDB00045 | https://hmdb.ca/metabolites/HMDB0000045 |
| Dihydro0yacetone phosphate | HMDB0001473 | https://hmdb.ca/metabolites/HMDB0001473 |
| Flavin mononucleotide (FMN) | HMDB0001520 | https://hmdb.ca/metabolites/HMDB0001520 |
| Fructose 1,6-bisphosphate | HMDB0001058 | https://hmdb.ca/metabolites/HMDB0001058 |
| Fumarate | HMDB00134 | https://hmdb.ca/metabolites/HMDB0000134 |
| GMP | HMDB01397 | https://hmdb.ca/metabolites/HMDB0001397 |
| Isocitrate | HMDB00193 | https://hmdb.ca/metabolites/HMDB0000193 |
| Malic acid | HMDB0000156 | https://hmdb.ca/metabolites/HMDB0000156 |
| Pyruvate | HMDB00243 | https://hmdb.ca/metabolites/HMDB0000243 |
| Cyclic AMP | HMDB00058 | https://hmdb.ca/metabolites/HMDB0000058 |
| Lactate | HMDB0001311 | https://hmdb.ca/metabolites/HMDB0001311 |
| NAD | HMDB0000902 | https://hmdb.ca/metabolites/HMDB0000902 |
| NADP | HMDB00217 | https://hmdb.ca/metabolites/HMDB0000217 |
| Oxaloacetate | HMDB0000223 | https://hmdb.ca/metabolites/HMDB0000223 |
| Phosphoenolpyruvate | HMDB0000263 | https://hmdb.ca/metabolites/HMDB0000263 |
| Succinate | HMDB0000254 | https://hmdb.ca/metabolites/HMDB0000254 |
| Fructose 6-phosphate | HMDB0003971 | https://hmdb.ca/metabolites/HMDB0003971 |
| Glucose 6-phosphate | HMDB0001401 | https://hmdb.ca/metabolites/HMDB0001401 |
|  |  |  |

Table S4 Toolkit used for data visualization

| Statistics | Toolkit (software/webaddress), version | Package |
| --- | --- | --- |
| Cluster heatmap | R,3.6.1 | pheatmap |
| Correlation heatmap | R,3.6.1 | corrplot |
| Venn diagram | <http://bioinformatics.psb.ugent.be/webtools/Venn/> |  |
| Bubble plot | <https://www.metaboanalyst.ca/> |  |
| Vocano plot | R,3.6.1 | ggplot2 |
| Scatter plot | R,3.6.1 | ggplot2 |
| GSEA | R,3.6.1 | clusterProfiler |

Table S5 Details of overlapped metabolites in OV and SI group from non-targeted metabolomics in hepatocytes

| KEGG | Name | HMDB | PubChem | ChEBI | METLIN | SMILES |
| --- | --- | --- | --- | --- | --- | --- |
| C00141 | Alpha-ketoisovaleric acid | HMDB0000019 | 49 | 16530 | 5091 | CC(C)C(=O)C(=O)O |
| C01530 | Stearic acid | HMDB0000827 | 5281 | 28842 | 189 | CCCCCCCCCCCCCCCCCC(O)=O |
| C08278 | Suberic acid | HMDB0000893 | 10457 | 9300 | 4243 | OC(=O)CCCCCCC(O)=O |
| C00612 | N1-Acetylspermidine | HMDB0001276 | 496 | 17927 | 3323 | CC(=O)NCCCNCCCCN |
| C01107 | Mevalonic acid-5P | HMDB0001343 | 439400 | 17436 | 6177 | C[C@@](O)(CCOP(O)(O)=O)CC(O)=O |
| C15589 | Chalcone | HMDB0003066 | 637760 | 48965 | 3342 | O=C(\C=C\C1=CC=CC=C1)C1=CC=CC=C1 |
| C00521 | D-Limonene | HMDB0003375 | 439250 | 15383 | 6911 | CC(=C)[C@H]1CCC(C)=CC1 |
| C00157 | PC(20:0/18:2(9Z,12Z)) | HMDB0008270 | 24779263 | - | - | CCCCCCCCCCCCCCCCCCCC(=O)OC[C@]([H])(COP([O-])(=O)OCC[N+](C)(C)C)OC(=O)CCCCCCC\C=C/C\C=C/CCCCC |
| C04230 | LysoPC(22:2(13Z,16Z)) | HMDB0010400 | 52924059 | - | - | CCCCC\C=C/C\C=C/CCCCCCCCCCCC(=O)OC[C@](O)([H])COP([O-])(=O)OCC[N+](C)(C)C |
| C04692 | 2-(3-Carboxy-3-(methylammonio)propyl)-L-histidine | HMDB0011654 | 440443 | 16475 | - | C[NH2+]C(CCC1=NC=C(N1)C[C@@H](C(=O)O)N)C(=O)O |
| C14467 | 1,2-Diphenylcyclobutane | HMDB0031821 | 140640 | - | - | C1CC(C1C1=CC=CC=C1)C1=CC=CC=C1 |
| C14487 | Ethisterone | HMDB0060580 | 5284557 | 34749 | - | C[C@]12CC[C@H]3[C@@H](CCC4=CC(=O)CC[C@]34C)[C@@H]1CC[C@@]2(O)C#C |
|  |  |  |  |  |  |  |

Table S6 Speculative pathways based on enriched energy metabolites that associated with PKLR variations

|  | Total | Expected | Hits | Raw p | Holm adjust | FDR | Impact |
| --- | --- | --- | --- | --- | --- | --- | --- |
| Citrate cycle (TCA cycle) | 20 | 0.077419 | 5 | 1.25E-09 | 1.05E-07 | 1.05E-07 | 0.17883 |
| Pyruvate metabolism | 22 | 0.085161 | 4 | 4.50E-07 | 3.73E-05 | 1.89E-05 | 0.23794 |
| Alanine, aspartate and glutamate metabolism | 28 | 0.10839 | 3 | 0.00010197 | 0.0083618 | 0.0028553 | 0.05048 |
| Arginine biosynthesis | 14 | 0.054194 | 2 | 0.0011137 | 0.090212 | 0.023388 | 0 |
| Glycolysis / Gluconeogenesis | 26 | 0.10065 | 2 | 0.0038958 | 0.31166 | 0.065449 | 0.20594 |
| Glyoxylate and dicarboxylate metabolism | 32 | 0.12387 | 2 | 0.0058839 | 0.46483 | 0.082375 | 0 |
| Tyrosine metabolism | 42 | 0.16258 | 2 | 0.010038 | 0.78294 | 0.12045 | 0.02463 |
| D-Glutamine and D-glutamate metabolism | 6 | 0.023226 | 1 | 0.023039 | 1 | 0.24191 | 0 |
| Butanoate metabolism | 15 | 0.058065 | 1 | 0.056767 | 1 | 0.52983 | 0 |
| Glycine, serine and threonine metabolism | 33 | 0.12774 | 1 | 0.12132 | 1 | 0.92643 | 0 |
| Cysteine and methionine metabolism | 33 | 0.12774 | 1 | 0.12132 | 1 | 0.92643 | 0 |
| Arginine and proline metabolism | 38 | 0.1471 | 1 | 0.13858 | 1 | 0.97006 | 0 |
| Purine metabolism | 65 | 0.25161 | 1 | 0.22699 | 1 | 1 | 0.06417 |
|  |  |  |  |  |  |  |  |

Table S7 KEGG pathways enrichment on differential genes from HepG2 cells with PKLR perturbation by GSEA

| Treat-  ment | ID | Description | Set  Size | enrichmentScore | NES | p.adjust |
| --- | --- | --- | --- | --- | --- | --- |
| OV | hsa01100 | Metabolic pathways | 333 | 0.277799584 | 1.981506907 | 0.000169495 |
| OV | hsa00260 | Glycine, serine and threonine metabolism | 18 | 0.619916063 | 2.309131414 | 0.004775731 |
| OV | hsa00982 | Drug metabolism - cytochrome P450 | 18 | 0.603324207 | 2.247328246 | 0.005582195 |
| OV | hsa03030 | DNA replication | 29 | 0.514413154 | 2.233766019 | 0.007638629 |
| OV | hsa00980 | Metabolism of xenobiotics by cytochrome P450 | 18 | 0.57558977 | 2.144019972 | 0.011109986 |
| OV | hsa00280 | Valine, leucine and isoleucine degradation | 20 | 0.550295642 | 2.119301494 | 0.022285398 |
| OV | hsa03320 | PPAR signaling pathway | 30 | 0.468554604 | 2.057247927 | 0.026819133 |
| OV | hsa00100 | Steroid biosynthesis | 16 | 0.565552699 | 2.01261678 | 0.027921011 |
| OV | hsa00650 | Butanoate metabolism | 17 | 0.550611046 | 1.995836861 | 0.041502045 |
| OV | hsa04080 | Neuroactive ligand-receptor interaction | 36 | -0.381811967 | -1.935647526 | 0.044156254 |
|  |  |  |  |  |  |  |
| SI | hsa01100 | Metabolic pathways | 435 | -0.260037741 | -1.850277589 | 0.000134473 |
| SI | hsa04975 | Fat digestion and absorption | 21 | -0.64463377 | -2.49261945 | 0.000604704 |
| SI | hsa04610 | Complement and coagulation cascades | 43 | -0.485660704 | -2.299278961 | 0.000949867 |
| SI | hsa00260 | Glycine, serine and threonine metabolism | 19 | -0.642132224 | -2.39396264 | 0.001460286 |
| SI | hsa04146 | Peroxisome | 34 | -0.509532484 | -2.256414446 | 0.003226215 |
| SI | hsa00591 | Linoleic acid metabolism | 11 | -0.727522002 | -2.305962786 | 0.003708689 |
| SI | hsa04080 | Neuroactive ligand-receptor interaction | 49 | 0.384143379 | 1.993692784 | 0.018433859 |
| SI | hsa03320 | PPAR signaling pathway | 39 | -0.436861166 | -2.011692653 | 0.018433859 |
| SI | hsa00565 | Ether lipid metabolism | 10 | -0.674073867 | -2.077460921 | 0.018433859 |
| SI | hsa05323 | Rheumatoid arthritis | 27 | 0.475687203 | 2.061037863 | 0.023777109 |
| SI | hsa00590 | Arachidonic acid metabolism | 23 | -0.506502575 | -2.00235691 | 0.023777109 |
| SI | hsa04514 | Cell adhesion molecules (CAMs) | 38 | -0.417571289 | -1.893874106 | 0.023777109 |
| SI | hsa04742 | Taste transduction | 11 | 0.636509472 | 2.059684822 | 0.024692846 |
| SI | hsa00650 | Butanoate metabolism | 19 | -0.547861077 | -2.042506045 | 0.024692846 |
| SI | hsa04974 | Protein digestion and absorption | 24 | -0.470351757 | -1.882885081 | 0.04823219 |

Abbreviations: GSEA, Gene Set Enrichment Analysis; OV, overexpression; SI, silence.

Table S8 Speculative pathways in hepatocytes with overexpressed PKLR by integrative analysis of transcriptomic and non-targeted metabolomic data

|  | Total | Expected | Hits | Raw p | NAME | Holm adjust | FDR |
| --- | --- | --- | --- | --- | --- | --- | --- |
| Glycerolipid metabolism | 35 | 6.0389 | 22 | 1.62E-09 | 8.7904 | 1.36E-07 | 1.36E-07 |
| Butanoate metabolism | 29 | 5.0037 | 17 | 5.40E-07 | 6.2674 | 4.48E-05 | 1.58E-05 |
| Glycolysis or Gluconeogenesis | 61 | 10.525 | 27 | 5.65E-07 | 6.248 | 4.63E-05 | 1.58E-05 |
| Glutathione metabolism | 56 | 9.6623 | 25 | 1.24E-06 | 5.9064 | 0.00010048 | 2.61E-05 |
| Ascorbate and aldarate metabolism | 13 | 2.243 | 10 | 3.71E-06 | 5.4307 | 0.00029674 | 6.23E-05 |
| Lysine degradation | 49 | 8.4545 | 19 | 0.00024956 | 3.6028 | 0.019715 | 0.0034939 |
| Glycerophospholipid metabolism | 86 | 14.839 | 27 | 0.0007826 | 3.1065 | 0.061043 | 0.0086384 |
| Linoleic acid metabolism | 17 | 2.9332 | 9 | 0.0008227 | 3.0848 | 0.063348 | 0.0086384 |
| Phenylalanine metabolism | 21 | 3.6234 | 10 | 0.0012177 | 2.9145 | 0.092546 | 0.011365 |
| Pyruvate metabolism | 45 | 7.7643 | 16 | 0.0022672 | 2.6445 | 0.17004 | 0.017902 |
| Drug metabolism - other enzymes | 70 | 12.078 | 22 | 0.0023444 | 2.63 | 0.17348 | 0.017902 |
| Synthesis and degradation of ketone bodies | 10 | 1.7254 | 6 | 0.0028689 | 2.5423 | 0.20943 | 0.020082 |
| Arginine biosynthesis | 27 | 4.6586 | 11 | 0.0032794 | 2.4842 | 0.23612 | 0.02119 |
| Glycine, serine and threonine metabolism | 68 | 11.733 | 21 | 0.0037125 | 2.4303 | 0.26359 | 0.022275 |
| Phosphatidylinositol signaling system | 74 | 12.768 | 22 | 0.0050425 | 2.2974 | 0.35298 | 0.028238 |
| alpha-Linolenic acid metabolism | 22 | 3.7959 | 9 | 0.0074725 | 2.1265 | 0.51561 | 0.039231 |
| Pentose phosphate pathway | 47 | 8.1094 | 15 | 0.0097081 | 2.0129 | 0.66015 | 0.046535 |
| Inositol phosphate metabolism | 69 | 11.905 | 20 | 0.0099718 | 2.0012 | 0.66811 | 0.046535 |
| beta-Alanine metabolism | 44 | 7.5918 | 14 | 0.012664 | 1.8974 | 0.83583 | 0.055546 |
| Fructose and mannose metabolism | 40 | 6.9016 | 13 | 0.013324 | 1.8754 | 0.86603 | 0.055546 |
| Terpenoid backbone biosynthesis | 36 | 6.2115 | 12 | 0.013886 | 1.8574 | 0.88874 | 0.055546 |
| Ether lipid metabolism | 39 | 6.7291 | 12 | 0.026539 | 1.5761 | 1 | 0.10133 |
| One carbon pool by folate | 31 | 5.3488 | 10 | 0.030077 | 1.5218 | 1 | 0.10985 |
| Glyoxylate and dicarboxylate metabolism | 56 | 9.6623 | 15 | 0.047162 | 1.3264 | 1 | 0.16492 |
| Retinol metabolism | 47 | 8.1094 | 13 | 0.049336 | 1.3068 | 1 | 0.16492 |
| Arachidonic acid metabolism | 81 | 13.976 | 20 | 0.053773 | 1.2694 | 1 | 0.16492 |
| Starch and sucrose metabolism | 43 | 7.4193 | 12 | 0.054286 | 1.2653 | 1 | 0.16492 |
| Pantothenate and CoA biosynthesis | 34 | 5.8664 | 10 | 0.054973 | 1.2599 | 1 | 0.16492 |
| Arginine and proline metabolism | 78 | 13.458 | 19 | 0.06663 | 1.1763 | 1 | 0.18856 |
| Valine, leucine and isoleucine degradation | 88 | 15.184 | 21 | 0.067343 | 1.1717 | 1 | 0.18856 |
| Nitrogen metabolism | 10 | 1.7254 | 4 | 0.077209 | 1.1123 | 1 | 0.20921 |
| Histidine metabolism | 32 | 5.5213 | 9 | 0.085644 | 1.0673 | 1 | 0.22482 |
| Alanine, aspartate and glutamate metabolism | 61 | 10.525 | 15 | 0.090086 | 1.0453 | 1 | 0.22931 |
| Propanoate metabolism | 48 | 8.282 | 12 | 0.11017 | 0.95795 | 1 | 0.26943 |
| Sphingolipid metabolism | 58 | 10.007 | 14 | 0.11226 | 0.94977 | 1 | 0.26943 |
| Neomycin, kanamycin and gentamicin biosynthesis | 4 | 0.69016 | 2 | 0.14005 | 0.85372 | 1 | 0.32678 |
| Pentose and glucuronate interconversions | 32 | 5.5213 | 8 | 0.17335 | 0.76109 | 1 | 0.39354 |
| D-Glutamine and D-glutamate metabolism | 10 | 1.7254 | 3 | 0.24107 | 0.61785 | 1 | 0.5329 |
| Galactose metabolism | 51 | 8.7996 | 11 | 0.25463 | 0.59409 | 1 | 0.54843 |
| Phenylalanine, tyrosine and tryptophan biosynthesis | 11 | 1.898 | 3 | 0.29195 | 0.53469 | 1 | 0.61305 |
| Porphyrin and chlorophyll metabolism | 53 | 9.1447 | 11 | 0.29923 | 0.524 | 1 | 0.61305 |
| Steroid biosynthesis | 82 | 14.148 | 16 | 0.33471 | 0.47533 | 1 | 0.65483 |
| Cysteine and methionine metabolism | 71 | 12.25 | 14 | 0.33521 | 0.47468 | 1 | 0.65483 |
| Valine, leucine and isoleucine biosynthesis | 12 | 2.0705 | 3 | 0.34343 | 0.46416 | 1 | 0.65565 |
| Sulfur metabolism | 18 | 3.1057 | 4 | 0.37808 | 0.42241 | 1 | 0.70576 |
| Nicotinate and nicotinamide metabolism | 42 | 7.2467 | 8 | 0.4413 | 0.35526 | 1 | 0.78871 |
| Citrate cycle (TCA cycle) | 42 | 7.2467 | 8 | 0.4413 | 0.35526 | 1 | 0.78871 |
| Riboflavin metabolism | 9 | 1.5529 | 2 | 0.47713 | 0.32136 | 1 | 0.83498 |
| Tryptophan metabolism | 84 | 14.493 | 15 | 0.4872 | 0.31229 | 1 | 0.83521 |
| Fatty acid degradation | 102 | 17.599 | 18 | 0.49981 | 0.30119 | 1 | 0.83969 |
| Drug metabolism - cytochrome P450 | 98 | 16.909 | 17 | 0.53353 | 0.27284 | 1 | 0.85437 |
| Phosphonate and phosphinate metabolism | 10 | 1.7254 | 2 | 0.53614 | 0.27072 | 1 | 0.85437 |
| Taurine and hypotaurine metabolism | 16 | 2.7607 | 3 | 0.53906 | 0.26836 | 1 | 0.85437 |
| Pyrimidine metabolism | 99 | 17.082 | 17 | 0.55207 | 0.25801 | 1 | 0.85877 |
| Glycosaminoglycan biosynthesis - chondroitin sulfate / dermatan sulfate | 18 | 3.1057 | 3 | 0.62362 | 0.20508 | 1 | 0.95244 |
| Tyrosine metabolism | 88 | 15.184 | 14 | 0.67747 | 0.16911 | 1 | 1 |
| D-Arginine and D-ornithine metabolism | 6 | 1.0352 | 1 | 0.67943 | 0.16785 | 1 | 1 |
| Thiamine metabolism | 14 | 2.4156 | 2 | 0.72435 | 0.14005 | 1 | 1 |
| Mucin type O-glycan biosynthesis | 22 | 3.7959 | 3 | 0.75892 | 0.1198 | 1 | 1 |
| Lipoic acid metabolism | 15 | 2.5881 | 2 | 0.75996 | 0.11921 | 1 | 1 |
| Metabolism of xenobiotics by cytochrome P450 | 145 | 25.018 | 22 | 0.78504 | 0.10511 | 1 | 1 |
| Ubiquinone and other terpenoid-quinone biosynthesis | 17 | 2.9332 | 2 | 0.81935 | 0.086529 | 1 | 1 |
| Selenocompound metabolism | 35 | 6.0389 | 4 | 0.8782 | 0.056407 | 1 | 1 |
| Amino sugar and nucleotide sugar metabolism | 79 | 13.631 | 10 | 0.89841 | 0.046525 | 1 | 1 |
| Glycosaminoglycan degradation | 44 | 7.5918 | 5 | 0.89922 | 0.046133 | 1 | 1 |
| Biotin metabolism | 21 | 3.6234 | 2 | 0.90022 | 0.045652 | 1 | 1 |
| Folate biosynthesis | 61 | 10.525 | 7 | 0.92273 | 0.034925 | 1 | 1 |
| Glycosylphosphatidylinositol (GPI)-anchor biosynthesis | 31 | 5.3488 | 3 | 0.92326 | 0.034677 | 1 | 1 |
| Biosynthesis of unsaturated fatty acids | 47 | 8.1094 | 5 | 0.92812 | 0.032395 | 1 | 1 |
| Purine metabolism | 166 | 28.642 | 22 | 0.93948 | 0.027111 | 1 | 1 |
| Fatty acid elongation | 75 | 12.941 | 8 | 0.96129 | 0.017146 | 1 | 1 |
| Mannose type O-glycan biosynthesis | 30 | 5.1762 | 2 | 0.97589 | 0.010599 | 1 | 1 |
| Glycosphingolipid biosynthesis - globo and isoglobo series | 31 | 5.3488 | 2 | 0.97952 | 0.0089868 | 1 | 1 |
| Primary bile acid biosynthesis | 92 | 15.874 | 9 | 0.98605 | 0.0061002 | 1 | 1 |
| N-Glycan biosynthesis | 77 | 13.286 | 5 | 0.99863 | 0.00059687 | 1 | 1 |
| Glycosphingolipid biosynthesis - ganglio series | 47 | 8.1094 | 1 | 0.99988 | 5.39E-05 | 1 | 1 |
| Aminoacyl-tRNA biosynthesis | 74 | 12.768 | 3 | 0.99991 | 4.02E-05 | 1 | 1 |
| Glycosphingolipid biosynthesis - lacto and neolacto series | 121 | 20.877 | 2 | 1 | 7.08E-10 | 1 | 1 |
| Steroid hormone biosynthesis | 199 | 34.336 | 12 | 1 | 1.30E-07 | 1 | 1 |
| Fatty acid biosynthesis | 129 | 22.258 | 6 | 1 | 9.04E-07 | 1 | 1 |
|  |  |  |  |  |  |  |  |

Table S9 Speculative pathways in hepatocytes with down-regulated PKLR by integrative analysis of transcriptomic and non-targeted metabolomic data

|  | Total | Expected | Hits | Raw p | NAME | Holm adjust | FDR |
| --- | --- | --- | --- | --- | --- | --- | --- |
| Glycerolipid metabolism | 35 | 7.7602 | 24 | 4.43E-09 | 8.3541 | 3.72E-07 | 3.72E-07 |
| Glycolysis or Gluconeogenesis | 61 | 13.525 | 29 | 8.16E-06 | 5.0881 | 0.00067766 | 0.00034291 |
| Butanoate metabolism | 29 | 6.4299 | 17 | 2.09E-05 | 4.6793 | 0.0017161 | 0.00058597 |
| Glutathione metabolism | 56 | 12.416 | 25 | 0.00012829 | 3.8918 | 0.010392 | 0.0026942 |
| Ascorbate and aldarate metabolism | 13 | 2.8824 | 9 | 0.00036668 | 3.4357 | 0.029334 | 0.0061602 |
| Retinol metabolism | 47 | 10.421 | 20 | 0.0012814 | 2.8923 | 0.10123 | 0.01794 |
| Pyruvate metabolism | 45 | 9.9775 | 19 | 0.0018872 | 2.7242 | 0.1472 | 0.020859 |
| Glycerophospholipid metabolism | 86 | 19.068 | 31 | 0.0019866 | 2.7019 | 0.15297 | 0.020859 |
| Linoleic acid metabolism | 17 | 3.7693 | 9 | 0.0052505 | 2.2798 | 0.39904 | 0.042964 |
| Valine, leucine and isoleucine degradation | 88 | 19.511 | 30 | 0.0060723 | 2.2166 | 0.45542 | 0.042964 |
| Drug metabolism - other enzymes | 70 | 15.52 | 25 | 0.0060899 | 2.2154 | 0.45542 | 0.042964 |
| Glyoxylate and dicarboxylate metabolism | 56 | 12.416 | 21 | 0.0061377 | 2.212 | 0.45542 | 0.042964 |
| Fructose and mannose metabolism | 40 | 8.8689 | 16 | 0.0079679 | 2.0987 | 0.57369 | 0.051485 |
| Nitrogen metabolism | 10 | 2.2172 | 6 | 0.010661 | 1.9722 | 0.75696 | 0.059704 |
| Synthesis and degradation of ketone bodies | 10 | 2.2172 | 6 | 0.010661 | 1.9722 | 0.75696 | 0.059704 |
| Phosphatidylinositol signaling system | 74 | 16.407 | 25 | 0.013343 | 1.8747 | 0.9207 | 0.070053 |
| One carbon pool by folate | 31 | 6.8734 | 12 | 0.027029 | 1.5682 | 1 | 0.13355 |
| Glycine, serine and threonine metabolism | 68 | 15.077 | 22 | 0.032357 | 1.49 | 1 | 0.14239 |
| Histidine metabolism | 32 | 7.0951 | 12 | 0.034824 | 1.4581 | 1 | 0.14239 |
| Neomycin, kanamycin and gentamicin biosynthesis | 4 | 0.88689 | 3 | 0.036224 | 1.441 | 1 | 0.14239 |
| alpha-Linolenic acid metabolism | 22 | 4.8779 | 9 | 0.037033 | 1.4314 | 1 | 0.14239 |
| Inositol phosphate metabolism | 69 | 15.299 | 22 | 0.037966 | 1.4206 | 1 | 0.14239 |
| Terpenoid backbone biosynthesis | 36 | 7.982 | 13 | 0.038989 | 1.4091 | 1 | 0.14239 |
| beta-Alanine metabolism | 44 | 9.7557 | 15 | 0.04586 | 1.3386 | 1 | 0.16051 |
| Propanoate metabolism | 48 | 10.643 | 16 | 0.04866 | 1.3128 | 1 | 0.1635 |
| Arginine biosynthesis | 27 | 5.9865 | 10 | 0.056469 | 1.2482 | 1 | 0.18049 |
| Lysine degradation | 49 | 10.864 | 16 | 0.058016 | 1.2365 | 1 | 0.18049 |
| Arachidonic acid metabolism | 81 | 17.959 | 24 | 0.069126 | 1.1604 | 1 | 0.20274 |
| Ether lipid metabolism | 39 | 8.6471 | 13 | 0.071604 | 1.1451 | 1 | 0.20274 |
| Phenylalanine metabolism | 21 | 4.6561 | 8 | 0.072407 | 1.1402 | 1 | 0.20274 |
| Starch and sucrose metabolism | 43 | 9.534 | 14 | 0.075053 | 1.1246 | 1 | 0.20337 |
| Alanine, aspartate and glutamate metabolism | 61 | 13.525 | 18 | 0.10951 | 0.96055 | 1 | 0.28746 |
| Riboflavin metabolism | 9 | 1.9955 | 4 | 0.11665 | 0.93312 | 1 | 0.29203 |
| Citrate cycle (TCA cycle) | 42 | 9.3123 | 13 | 0.1182 | 0.92737 | 1 | 0.29203 |
| Pentose phosphate pathway | 47 | 10.421 | 14 | 0.13829 | 0.85922 | 1 | 0.33189 |
| Arginine and proline metabolism | 78 | 17.294 | 21 | 0.18577 | 0.73101 | 1 | 0.43344 |
| Glycosaminoglycan biosynthesis - chondroitin sulfate / dermatan sulfate | 18 | 3.991 | 6 | 0.19092 | 0.71915 | 1 | 0.43344 |
| Sphingolipid metabolism | 58 | 12.86 | 16 | 0.19681 | 0.70596 | 1 | 0.43417 |
| Pantothenate and CoA biosynthesis | 34 | 7.5385 | 10 | 0.20369 | 0.69102 | 1 | 0.43417 |
| Cysteine and methionine metabolism | 71 | 15.742 | 19 | 0.2093 | 0.67923 | 1 | 0.43417 |
| Phenylalanine, tyrosine and tryptophan biosynthesis | 11 | 2.4389 | 4 | 0.21192 | 0.67383 | 1 | 0.43417 |
| Pentose and glucuronate interconversions | 32 | 7.0951 | 9 | 0.26565 | 0.5757 | 1 | 0.51639 |
| Valine, leucine and isoleucine biosynthesis | 12 | 2.6607 | 4 | 0.26566 | 0.57567 | 1 | 0.51639 |
| Taurine and hypotaurine metabolism | 16 | 3.5475 | 5 | 0.27049 | 0.56785 | 1 | 0.51639 |
| Phosphonate and phosphinate metabolism | 10 | 2.2172 | 3 | 0.38839 | 0.41074 | 1 | 0.70923 |
| D-Glutamine and D-glutamate metabolism | 10 | 2.2172 | 3 | 0.38839 | 0.41074 | 1 | 0.70923 |
| Galactose metabolism | 51 | 11.308 | 12 | 0.46182 | 0.33553 | 1 | 0.82538 |
| Glycosaminoglycan biosynthesis - heparan sulfate / heparin | 7 | 1.552 | 2 | 0.4823 | 0.31668 | 1 | 0.84403 |
| Porphyrin and chlorophyll metabolism | 53 | 11.751 | 12 | 0.52151 | 0.28274 | 1 | 0.89402 |
| Mucin type O-glycan biosynthesis | 22 | 4.8779 | 5 | 0.55842 | 0.25304 | 1 | 0.93815 |
| Sulfur metabolism | 18 | 3.991 | 4 | 0.59 | 0.22915 | 1 | 0.9666 |
| Nicotinate and nicotinamide metabolism | 42 | 9.3123 | 9 | 0.60737 | 0.21655 | 1 | 0.9666 |
| Tryptophan metabolism | 84 | 18.625 | 18 | 0.60988 | 0.21476 | 1 | 0.9666 |
| Thiamine metabolism | 14 | 3.1041 | 3 | 0.63045 | 0.20035 | 1 | 0.98069 |
| Fatty acid degradation | 102 | 22.616 | 21 | 0.69125 | 0.16037 | 1 | 1 |
| Amino sugar and nucleotide sugar metabolism | 79 | 17.516 | 16 | 0.70465 | 0.15203 | 1 | 1 |
| Purine metabolism | 166 | 36.806 | 34 | 0.73578 | 0.13325 | 1 | 1 |
| N-Glycan biosynthesis | 77 | 17.073 | 15 | 0.75955 | 0.11944 | 1 | 1 |
| Steroid biosynthesis | 82 | 18.181 | 16 | 0.76223 | 0.11791 | 1 | 1 |
| D-Arginine and D-ornithine metabolism | 6 | 1.3303 | 1 | 0.77816 | 0.10893 | 1 | 1 |
| Pyrimidine metabolism | 99 | 21.95 | 19 | 0.80136 | 0.096174 | 1 | 1 |
| Selenocompound metabolism | 35 | 7.7602 | 6 | 0.82163 | 0.085325 | 1 | 1 |
| Glycosylphosphatidylinositol (GPI)-anchor biosynthesis | 31 | 6.8734 | 5 | 0.8503 | 0.070427 | 1 | 1 |
| Drug metabolism - cytochrome P450 | 98 | 21.729 | 18 | 0.85374 | 0.068673 | 1 | 1 |
| Lipoic acid metabolism | 15 | 3.3258 | 2 | 0.878 | 0.056507 | 1 | 1 |
| Glycosaminoglycan degradation | 44 | 9.7557 | 7 | 0.887 | 0.052076 | 1 | 1 |
| Ubiquinone and other terpenoid-quinone biosynthesis | 17 | 3.7693 | 2 | 0.91833 | 0.037001 | 1 | 1 |
| Mannose type O-glycan biosynthesis | 30 | 6.6516 | 4 | 0.92604 | 0.033368 | 1 | 1 |
| Glycosphingolipid biosynthesis - globo and isoglobo series | 31 | 6.8734 | 4 | 0.93727 | 0.028137 | 1 | 1 |
| Tyrosine metabolism | 88 | 19.511 | 14 | 0.94649 | 0.023884 | 1 | 1 |
| Biotin metabolism | 21 | 4.6561 | 2 | 0.96443 | 0.015729 | 1 | 1 |
| Folate biosynthesis | 61 | 13.525 | 8 | 0.9758 | 0.010638 | 1 | 1 |
| Metabolism of xenobiotics by cytochrome P450 | 145 | 32.15 | 22 | 0.98851 | 0.0050174 | 1 | 1 |
| Caffeine metabolism | 19 | 4.2127 | 1 | 0.99163 | 0.0036514 | 1 | 1 |
| Fatty acid elongation | 75 | 16.629 | 9 | 0.99263 | 0.0032142 | 1 | 1 |
| Vitamin B6 metabolism | 21 | 4.6561 | 1 | 0.99495 | 0.0021979 | 1 | 1 |
| Primary bile acid biosynthesis | 92 | 20.398 | 11 | 0.99647 | 0.0015369 | 1 | 1 |
| Glycosphingolipid biosynthesis - ganglio series | 47 | 10.421 | 4 | 0.99659 | 0.0014843 | 1 | 1 |
| Aminoacyl-tRNA biosynthesis | 74 | 16.407 | 8 | 0.99675 | 0.001413 | 1 | 1 |
| Biosynthesis of unsaturated fatty acids | 47 | 10.421 | 3 | 0.99928 | 0.00031161 | 1 | 1 |
| Steroid hormone biosynthesis | 199 | 44.123 | 22 | 0.99999 | 3.41E-06 | 1 | 1 |
| Glycosphingolipid biosynthesis - lacto and neolacto series | 121 | 26.828 | 10 | 0.99999 | 3.75E-06 | 1 | 1 |
| Fatty acid biosynthesis | 129 | 28.602 | 8 | 1 | 4.15E-08 | 1 | 1 |
|  |  |  |  |  |  |  |  |

Table S10 Speculative pathways in hepatocytes with overexpressed PKLR by integrative analysis of transcriptomic and targeted metabolomic data

|  | Total | Expected | Hits | Raw p | NAME | Holm adjust | FDR |
| --- | --- | --- | --- | --- | --- | --- | --- |
| Glycerolipid metabolism | 35 | 5.8094 | 21 | 6.25E-09 | 8.2038 | 5.25E-07 | 5.25E-07 |
| Glycolysis or Gluconeogenesis | 61 | 10.125 | 28 | 5.58E-08 | 7.2537 | 4.63E-06 | 2.34E-06 |
| Butanoate metabolism | 29 | 4.8135 | 17 | 3.01E-07 | 6.5209 | 2.47E-05 | 8.44E-06 |
| Glutathione metabolism | 56 | 9.2951 | 24 | 2.42E-06 | 5.6158 | 0.00019621 | 5.09E-05 |
| Ascorbate and aldarate metabolism | 13 | 2.1578 | 9 | 3.37E-05 | 4.4723 | 0.0026962 | 0.00050975 |
| Pyruvate metabolism | 45 | 7.4693 | 19 | 3.64E-05 | 4.4388 | 0.0028765 | 0.00050975 |
| Arginine biosynthesis | 27 | 4.4816 | 13 | 0.00012847 | 3.8912 | 0.010021 | 0.0015378 |
| Lysine degradation | 49 | 8.1332 | 19 | 0.00014646 | 3.8343 | 0.011277 | 0.0015378 |
| Phenylalanine metabolism | 21 | 3.4857 | 10 | 0.00088817 | 3.0515 | 0.067501 | 0.0082896 |
| Drug metabolism - other enzymes | 70 | 11.619 | 22 | 0.0013911 | 2.8566 | 0.10433 | 0.011685 |
| Glycine, serine and threonine metabolism | 68 | 11.287 | 21 | 0.002274 | 2.6432 | 0.16828 | 0.017365 |
| Glycerophospholipid metabolism | 86 | 14.275 | 24 | 0.0050466 | 2.297 | 0.3684 | 0.035326 |
| Pentose phosphate pathway | 47 | 7.8012 | 15 | 0.0067447 | 2.171 | 0.48562 | 0.041481 |
| Phosphatidylinositol signaling system | 74 | 12.283 | 21 | 0.0069135 | 2.1603 | 0.49086 | 0.041481 |
| Citrate cycle (TCA cycle) | 42 | 6.9713 | 12 | 0.0073528 | 2.14524 | 0.52361 | 0.041857 |
| beta-Alanine metabolism | 44 | 7.3033 | 14 | 0.0090146 | 2.0451 | 0.63102 | 0.050482 |
| Fructose and mannose metabolism | 40 | 6.6393 | 13 | 0.0096523 | 2.0154 | 0.66601 | 0.050675 |
| Inositol phosphate metabolism | 69 | 11.453 | 19 | 0.013967 | 1.8549 | 0.94977 | 0.065579 |
| Linoleic acid metabolism | 17 | 2.8217 | 7 | 0.014053 | 1.8522 | 0.94977 | 0.065579 |
| Synthesis and degradation of ketone bodies | 10 | 1.6598 | 5 | 0.014999 | 1.8239 | 0.98991 | 0.06631 |
| Alanine, aspartate and glutamate metabolism | 61 | 10.125 | 17 | 0.017365 | 1.7603 | 1 | 0.072932 |
| alpha-Linolenic acid metabolism | 22 | 3.6516 | 8 | 0.019974 | 1.6995 | 1 | 0.079896 |
| One carbon pool by folate | 31 | 5.1455 | 10 | 0.023431 | 1.6302 | 1 | 0.089463 |
| Terpenoid backbone biosynthesis | 36 | 5.9754 | 11 | 0.026748 | 1.5727 | 1 | 0.097689 |
| Glyoxylate and dicarboxylate metabolism | 56 | 9.2951 | 15 | 0.034781 | 1.4587 | 1 | 0.11857 |
| Retinol metabolism | 47 | 7.8012 | 13 | 0.037413 | 1.427 | 1 | 0.12087 |
| Starch and sucrose metabolism | 43 | 7.1373 | 12 | 0.041883 | 1.378 | 1 | 0.1303 |
| Arginine and proline metabolism | 78 | 12.947 | 19 | 0.047746 | 1.3211 | 1 | 0.14324 |
| Arachidonic acid metabolism | 81 | 13.445 | 19 | 0.066718 | 1.1758 | 1 | 0.18388 |
| Nitrogen metabolism | 10 | 1.6598 | 4 | 0.068442 | 1.1647 | 1 | 0.18388 |
| D-Glutamine and D-glutamate metabolism | 10 | 1.6598 | 4 | 0.068442 | 1.1647 | 1 | 0.18388 |
| Histidine metabolism | 32 | 5.3115 | 9 | 0.070048 | 1.1546 | 1 | 0.18388 |
| Propanoate metabolism | 48 | 7.9672 | 12 | 0.087671 | 1.0571 | 1 | 0.22316 |
| Pantothenate and CoA biosynthesis | 34 | 5.6434 | 9 | 0.096958 | 1.0134 | 1 | 0.23697 |
| Ether lipid metabolism | 39 | 6.4734 | 10 | 0.098737 | 1.0055 | 1 | 0.23697 |
| Valine, leucine and isoleucine degradation | 88 | 14.607 | 19 | 0.12927 | 0.8885 | 1 | 0.29707 |
| Neomycin, kanamycin and gentamicin biosynthesis | 4 | 0.66393 | 2 | 0.13085 | 0.88321 | 1 | 0.29707 |
| Pentose and glucuronate interconversions | 32 | 5.3115 | 8 | 0.14767 | 0.83071 | 1 | 0.32643 |
| Sphingolipid metabolism | 58 | 9.627 | 12 | 0.24513 | 0.6106 | 1 | 0.52798 |
| Porphyrin and chlorophyll metabolism | 53 | 8.7971 | 11 | 0.25489 | 0.59365 | 1 | 0.53527 |
| Phenylalanine, tyrosine and tryptophan biosynthesis | 11 | 1.8258 | 3 | 0.27082 | 0.56732 | 1 | 0.54988 |
| Steroid biosynthesis | 82 | 13.611 | 16 | 0.27714 | 0.5573 | 1 | 0.54988 |
| Cysteine and methionine metabolism | 71 | 11.785 | 14 | 0.28148 | 0.55055 | 1 | 0.54988 |
| Galactose metabolism | 51 | 8.4652 | 10 | 0.3345 | 0.47561 | 1 | 0.63859 |
| Sulfur metabolism | 18 | 2.9877 | 4 | 0.34897 | 0.45721 | 1 | 0.65141 |
| Nicotinate and nicotinamide metabolism | 42 | 6.9713 | 8 | 0.39591 | 0.4024 | 1 | 0.72298 |
| Tryptophan metabolism | 84 | 13.943 | 15 | 0.42215 | 0.37454 | 1 | 0.74868 |
| Fatty acid degradation | 102 | 16.93 | 18 | 0.42782 | 0.36874 | 1 | 0.74868 |
| Riboflavin metabolism | 9 | 1.4939 | 2 | 0.45528 | 0.34172 | 1 | 0.77768 |
| Drug metabolism - cytochrome P450 | 98 | 16.266 | 17 | 0.4629 | 0.33451 | 1 | 0.77768 |
| Pyrimidine metabolism | 99 | 16.432 | 17 | 0.48123 | 0.31765 | 1 | 0.79262 |
| Taurine and hypotaurine metabolism | 16 | 2.6557 | 3 | 0.51067 | 0.29186 | 1 | 0.81377 |
| Phosphonate and phosphinate metabolism | 10 | 1.6598 | 2 | 0.51345 | 0.2895 | 1 | 0.81377 |
| Glycosaminoglycan biosynthesis - chondroitin sulfate / dermatan sulfate | 18 | 2.9877 | 3 | 0.59507 | 0.22543 | 1 | 0.92567 |
| Valine, leucine and isoleucine biosynthesis | 12 | 1.9918 | 2 | 0.61684 | 0.20983 | 1 | 0.94208 |
| D-Arginine and D-ornithine metabolism | 6 | 0.9959 | 1 | 0.66386 | 0.17792 | 1 | 0.9958 |
| Thiamine metabolism | 14 | 2.3238 | 2 | 0.70251 | 0.15334 | 1 | 1 |
| Tyrosine metabolism | 88 | 14.607 | 13 | 0.72415 | 0.14017 | 1 | 1 |
| Mucin type O-glycan biosynthesis | 22 | 3.6516 | 3 | 0.7333 | 0.13472 | 1 | 1 |
| Lipoic acid metabolism | 15 | 2.4898 | 2 | 0.73903 | 0.13134 | 1 | 1 |
| Metabolism of xenobiotics by cytochrome P450 | 145 | 24.068 | 21 | 0.79209 | 0.10123 | 1 | 1 |
| Ubiquinone and other terpenoid-quinone biosynthesis | 17 | 2.8217 | 2 | 0.80067 | 0.096547 | 1 | 1 |
| Selenocompound metabolism | 35 | 5.8094 | 4 | 0.85705 | 0.066992 | 1 | 1 |
| Amino sugar and nucleotide sugar metabolism | 79 | 13.113 | 10 | 0.86881 | 0.061075 | 1 | 1 |
| Glycosaminoglycan degradation | 44 | 7.3033 | 5 | 0.87823 | 0.056393 | 1 | 1 |
| Folate biosynthesis | 61 | 10.125 | 7 | 0.9019 | 0.044842 | 1 | 1 |
| Purine metabolism | 166 | 27.553 | 22 | 0.90748 | 0.042163 | 1 | 1 |
| Fatty acid elongation | 75 | 12.449 | 8 | 0.94752 | 0.02341 | 1 | 1 |
| Mannose type O-glycan biosynthesis | 30 | 4.9795 | 2 | 0.9706 | 0.012958 | 1 | 1 |
| Glycosylphosphatidylinositol (GPI)-anchor biosynthesis | 31 | 5.1455 | 2 | 0.97483 | 0.01107 | 1 | 1 |
| Glycosphingolipid biosynthesis - globo and isoglobo series | 31 | 5.1455 | 2 | 0.97483 | 0.01107 | 1 | 1 |
| Biotin metabolism | 21 | 3.4857 | 1 | 0.97826 | 0.009544 | 1 | 1 |
| Primary bile acid biosynthesis | 92 | 15.27 | 9 | 0.97932 | 0.009076 | 1 | 1 |
| N-Glycan biosynthesis | 77 | 12.781 | 5 | 0.99786 | 0.00093145 | 1 | 1 |
| Biosynthesis of unsaturated fatty acids | 47 | 7.8012 | 2 | 0.99809 | 0.00082918 | 1 | 1 |
| Glycosphingolipid biosynthesis - ganglio series | 47 | 7.8012 | 1 | 0.99982 | 7.84E-05 | 1 | 1 |
| Aminoacyl-tRNA biosynthesis | 74 | 12.283 | 3 | 0.99985 | 6.67E-05 | 1 | 1 |
| Glycosphingolipid biosynthesis - lacto and neolacto series | 121 | 20.084 | 2 | 1 | 1.81E-09 | 1 | 1 |
| Steroid hormone biosynthesis | 199 | 33.031 | 12 | 1 | 4.07E-07 | 1 | 1 |
| Fatty acid biosynthesis | 129 | 21.412 | 6 | 1 | 2.06E-06 | 1 | 1 |
|  |  |  |  |  |  |  |  |

Table S11 Speculative pathways in hepatocytes with down-regulated PKLR by integrative analysis of transcriptomic and targeted metabolomic data

|  | Total | Expected | Hits | Raw p | NAME | Holm adjust | FDR |
| --- | --- | --- | --- | --- | --- | --- | --- |
| Glycerolipid metabolism | 35 | 7.5451 | 24 | 2.41E-09 | 8.6181 | 2.02E-07 | 2.02E-07 |
| Glycolysis or Gluconeogenesis | 61 | 13.15 | 31 | 3.18E-07 | 6.4975 | 2.64E-05 | 1.34E-05 |
| Butanoate metabolism | 29 | 6.2516 | 18 | 2.44E-06 | 5.6129 | 0.00019996 | 6.83E-05 |
| Pyruvate metabolism | 45 | 9.7008 | 22 | 3.84E-05 | 4.4153 | 0.0031134 | 0.00080718 |
| Glutathione metabolism | 56 | 12.072 | 25 | 7.86E-05 | 4.1045 | 0.0062886 | 0.0013206 |
| Ascorbate and aldarate metabolism | 13 | 2.8025 | 9 | 0.00029202 | 3.5346 | 0.02307 | 0.0040883 |
| Retinol metabolism | 47 | 10.132 | 20 | 0.00087923 | 3.0559 | 0.06858 | 0.010551 |
| Glyoxylate and dicarboxylate metabolism | 56 | 12.072 | 22 | 0.0017675 | 2.7527 | 0.13609 | 0.018558 |
| Citrate cycle (TCA cycle) | 42 | 9.0541 | 17 | 0.0040254 | 2.3952 | 0.30593 | 0.034639 |
| Drug metabolism - other enzymes | 70 | 15.09 | 25 | 0.0041237 | 2.3847 | 0.30928 | 0.034639 |
| Glycerophospholipid metabolism | 86 | 18.539 | 29 | 0.0054211 | 2.2659 | 0.40116 | 0.040585 |
| Fructose and mannose metabolism | 40 | 8.623 | 16 | 0.0059834 | 2.2231 | 0.43679 | 0.040585 |
| Arginine biosynthesis | 27 | 5.8205 | 12 | 0.006281 | 2.202 | 0.45223 | 0.040585 |
| Nitrogen metabolism | 10 | 2.1557 | 6 | 0.0092315 | 2.0347 | 0.65543 | 0.048775 |
| Synthesis and degradation of ketone bodies | 10 | 2.1557 | 6 | 0.0092315 | 2.0347 | 0.65543 | 0.048775 |
| Phosphatidylinositol signaling system | 74 | 15.952 | 25 | 0.0092904 | 2.032 | 0.65543 | 0.048775 |
| Alanine, aspartate and glutamate metabolism | 61 | 13.15 | 21 | 0.013137 | 1.8815 | 0.8933 | 0.064911 |
| Valine, leucine and isoleucine degradation | 88 | 18.97 | 28 | 0.014778 | 1.8304 | 0.99015 | 0.068965 |
| Linoleic acid metabolism | 17 | 3.6648 | 8 | 0.016836 | 1.7738 | 1 | 0.074435 |
| One carbon pool by folate | 31 | 6.6828 | 12 | 0.021885 | 1.6599 | 1 | 0.091915 |
| Glycine, serine and threonine metabolism | 68 | 14.659 | 22 | 0.023946 | 1.6208 | 1 | 0.095784 |
| Inositol phosphate metabolism | 69 | 14.875 | 22 | 0.028285 | 1.5484 | 1 | 0.108 |
| Neomycin, kanamycin and gentamicin biosynthesis | 4 | 0.8623 | 3 | 0.033472 | 1.4753 | 1 | 0.12225 |
| beta-Alanine metabolism | 44 | 9.4852 | 15 | 0.036565 | 1.4369 | 1 | 0.12798 |
| Lysine degradation | 49 | 10.563 | 16 | 0.046227 | 1.3351 | 1 | 0.15532 |
| Ether lipid metabolism | 39 | 8.4074 | 13 | 0.059026 | 1.229 | 1 | 0.1907 |
| Starch and sucrose metabolism | 43 | 9.2697 | 14 | 0.061425 | 1.2117 | 1 | 0.1911 |
| Histidine metabolism | 32 | 6.8984 | 11 | 0.064765 | 1.1887 | 1 | 0.19429 |
| Terpenoid backbone biosynthesis | 36 | 7.7607 | 12 | 0.068249 | 1.1659 | 1 | 0.19769 |
| Propanoate metabolism | 48 | 10.348 | 15 | 0.07458 | 1.1274 | 1 | 0.20883 |
| alpha-Linolenic acid metabolism | 22 | 4.7426 | 8 | 0.080776 | 1.0927 | 1 | 0.21888 |
| Pentose phosphate pathway | 47 | 10.132 | 14 | 0.11598 | 0.93563 | 1 | 0.30444 |
| Arachidonic acid metabolism | 81 | 17.461 | 22 | 0.13443 | 0.8715 | 1 | 0.34219 |
| Phenylalanine metabolism | 21 | 4.527 | 7 | 0.14668 | 0.83364 | 1 | 0.35304 |
| D-Glutamine and D-glutamate metabolism | 10 | 2.1557 | 4 | 0.14945 | 0.82551 | 1 | 0.35304 |
| Arginine and proline metabolism | 78 | 16.815 | 21 | 0.1513 | 0.82016 | 1 | 0.35304 |
| Glycosaminoglycan biosynthesis - chondroitin sulfate / dermatan sulfate | 18 | 3.8803 | 6 | 0.17318 | 0.76151 | 1 | 0.39316 |
| Pentose and glucuronate interconversions | 32 | 6.8984 | 9 | 0.23771 | 0.62395 | 1 | 0.52311 |
| Taurine and hypotaurine metabolism | 16 | 3.4492 | 5 | 0.25011 | 0.60187 | 1 | 0.52311 |
| Sphingolipid metabolism | 58 | 12.503 | 15 | 0.25396 | 0.59523 | 1 | 0.52311 |
| Cysteine and methionine metabolism | 71 | 15.306 | 18 | 0.25533 | 0.5929 | 1 | 0.52311 |
| Pantothenate and CoA biosynthesis | 34 | 7.3295 | 9 | 0.3014 | 0.52086 | 1 | 0.59291 |
| Riboflavin metabolism | 9 | 1.9402 | 3 | 0.30351 | 0.51782 | 1 | 0.59291 |
| Phosphonate and phosphinate metabolism | 10 | 2.1557 | 3 | 0.36954 | 0.43234 | 1 | 0.70549 |
| Galactose metabolism | 51 | 10.994 | 12 | 0.41905 | 0.37773 | 1 | 0.78223 |
| Phenylalanine, tyrosine and tryptophan biosynthesis | 11 | 2.3713 | 3 | 0.43431 | 0.3622 | 1 | 0.79309 |
| Glycosaminoglycan biosynthesis - heparan sulfate / heparin | 7 | 1.509 | 2 | 0.46584 | 0.33176 | 1 | 0.83257 |
| Porphyrin and chlorophyll metabolism | 53 | 11.425 | 12 | 0.47762 | 0.32092 | 1 | 0.83584 |
| Mucin type O-glycan biosynthesis | 22 | 4.7426 | 5 | 0.53042 | 0.27538 | 1 | 0.90929 |
| Tryptophan metabolism | 84 | 18.108 | 18 | 0.5559 | 0.25501 | 1 | 0.92013 |
| Sulfur metabolism | 18 | 3.8803 | 4 | 0.56507 | 0.2479 | 1 | 0.92013 |
| Nicotinate and nicotinamide metabolism | 42 | 9.0541 | 9 | 0.5696 | 0.24443 | 1 | 0.92013 |
| Thiamine metabolism | 14 | 3.018 | 3 | 0.60914 | 0.21528 | 1 | 0.96543 |
| Fatty acid degradation | 102 | 21.989 | 21 | 0.63574 | 0.19672 | 1 | 0.98893 |
| Amino sugar and nucleotide sugar metabolism | 79 | 17.03 | 16 | 0.65714 | 0.18234 | 1 | 1 |
| N-Glycan biosynthesis | 77 | 16.599 | 15 | 0.71706 | 0.14445 | 1 | 1 |
| Purine metabolism | 166 | 35.785 | 33 | 0.73651 | 0.13282 | 1 | 1 |
| D-Arginine and D-ornithine metabolism | 6 | 1.2934 | 1 | 0.76742 | 0.11497 | 1 | 1 |
| Valine, leucine and isoleucine biosynthesis | 12 | 2.5869 | 2 | 0.76743 | 0.11496 | 1 | 1 |
| Selenocompound metabolism | 35 | 7.5451 | 6 | 0.79827 | 0.097851 | 1 | 1 |
| Steroid biosynthesis | 82 | 17.677 | 15 | 0.80565 | 0.093856 | 1 | 1 |
| Drug metabolism - cytochrome P450 | 98 | 21.126 | 18 | 0.81743 | 0.087547 | 1 | 1 |
| Pyrimidine metabolism | 99 | 21.342 | 18 | 0.83078 | 0.080513 | 1 | 1 |
| Glycosylphosphatidylinositol (GPI)-anchor biosynthesis | 31 | 6.6828 | 5 | 0.83079 | 0.080507 | 1 | 1 |
| Lipoic acid metabolism | 15 | 3.2336 | 2 | 0.8666 | 0.062181 | 1 | 1 |
| Glycosaminoglycan degradation | 44 | 9.4852 | 7 | 0.86755 | 0.061705 | 1 | 1 |
| Tyrosine metabolism | 88 | 18.97 | 15 | 0.8833 | 0.053893 | 1 | 1 |
| Mannose type O-glycan biosynthesis | 30 | 6.4672 | 4 | 0.9146 | 0.038771 | 1 | 1 |
| Glycosphingolipid biosynthesis - globo and isoglobo series | 31 | 6.6828 | 4 | 0.92701 | 0.032915 | 1 | 1 |
| Biotin metabolism | 21 | 4.527 | 2 | 0.95928 | 0.018055 | 1 | 1 |
| Folate biosynthesis | 61 | 13.15 | 8 | 0.96879 | 0.013773 | 1 | 1 |
| Metabolism of xenobiotics by cytochrome P450 | 145 | 31.258 | 22 | 0.98204 | 0.0078729 | 1 | 1 |
| Ubiquinone and other terpenoid-quinone biosynthesis | 17 | 3.6648 | 1 | 0.98413 | 0.0069495 | 1 | 1 |
| Fatty acid elongation | 75 | 16.168 | 9 | 0.98974 | 0.0044781 | 1 | 1 |
| Caffeine metabolism | 19 | 4.0959 | 1 | 0.99027 | 0.004246 | 1 | 1 |
| Vitamin B6 metabolism | 21 | 4.527 | 1 | 0.99404 | 0.0025962 | 1 | 1 |
| Primary bile acid biosynthesis | 92 | 19.833 | 11 | 0.99474 | 0.0022884 | 1 | 1 |
| Glycosphingolipid biosynthesis - ganglio series | 47 | 10.132 | 4 | 0.99551 | 0.0019543 | 1 | 1 |
| Aminoacyl-tRNA biosynthesis | 74 | 15.952 | 7 | 0.99843 | 0.00068308 | 1 | 1 |
| Biosynthesis of unsaturated fatty acids | 47 | 10.132 | 2 | 0.99986 | 6.05E-05 | 1 | 1 |
| Glycosphingolipid biosynthesis - lacto and neolacto series | 121 | 26.084 | 10 | 0.99998 | 7.29E-06 | 1 | 1 |
| Steroid hormone biosynthesis | 199 | 42.899 | 21 | 0.99999 | 3.24E-06 | 1 | 1 |
| Fatty acid biosynthesis | 129 | 27.809 | 7 | 1 | 1.75E-08 | 1 | 1 |
|  |  |  |  |  |  |  |  |

Table S12 Potential pathways overlapped between transcriptomics and non-targeted metabolomics data in OV and SI group

| Matched pathway | number of metabolites in OV group | details of metabolites in OV group | number of metabolites in SI group | drtails of metabolites in SI group |
| --- | --- | --- | --- | --- |
| Glycerolipid metabolism | 1 | cpd:C00184 | 0 |  |
| Glycolysis or Gluconeogenesis | 0 |  | 0 |  |
| Butanoate metabolism | 1 | cpd:C00164 | 0 |  |
| Glutathione metabolism | 1 | cpd:C01879 | 0 |  |
| Ascorbate and aldarate metabolism | 1 | cpd:C00137 | 0 |  |
| Pyruvate metabolism | 0 |  | 1 | cpd:C00149 |
| Glycerophospholipid metabolism | 3 | cpd:C00350; cpd:C00157; cpd:C04230 | 2 | cpd:C00157; cpd:C04230 |
| Linoleic acid metabolism | 2 | cpd:C01595; cpd:C00157 | 1 | cpd:C00157 |
| Drug metabolism - other enzymes | 0 |  | 0 |  |
| Phosphatidylinositol signaling system | 1 | cpd:C00137 | 0 |  |
|  |  |  |  |  |

Table S13 Potential pathways overlapped between transcriptomics and targeted metabolomics data in OV and SI group

| Matched pathway | number of metabolites in OV group | details of metabolites in OV group | number of metabolites in SI group | drtails of metabolites in SI group |
| --- | --- | --- | --- | --- |
| Glycerolipid metabolism | 0 |  | 0 |  |
| Glycolysis or Gluconeogenesis | 1 | cpd:C00074 | 1 | cpd:C00022; cpd:C00074 |
| Butanoate metabolism | 1 | cpd:C00026 | 1 | cpd:C00026 |
| Pyruvate metabolism | 3 | cpd:C00074; cpd:C00149; cpd:C00122 | 4 | cpd:C00074; cpd:C00022; cpd:C00149; cpd:C00122 |
| Glutathione metabolism | 0 |  | 0 |  |
| Ascorbate and aldarate metabolism | 0 |  | 0 |  |
| Drug metabolism - other enzymes | 0 |  | 0 |  |
| Glycerophospholipid metabolism | 0 |  | 0 |  |
| Arginine biosynthesis | 2 | cpd:C00026; cpd:C00122 | 2 | cpd:C00026; cpd:C00122 |
| Phosphatidylinositol signaling system | 0 |  | 0 |  |
| Citrate cycle (TCA cycle) | 4 | cpd:C00026; cpd:C00149; cpd:C00122; cpd:C00074; | 5 | cpd:C00026; cpd:C00149; cpd:C00022; cpd:C00122; cpd:C00074 |
|  |  |  |  |  |

Table S14 Impact of graft PKM and PKLR expression on post-transplant prognosis

| Variables | Univariated HR of PS (95%CI, H vs. L) | P1 | Univariated HR of GS (95%CI, H vs. L) | P2 |
| --- | --- | --- | --- | --- |
| PKLR | 0.37(0.24-0.64) | <0.01 | 0.49(0.32-0.81) | <0.01 |
| PKM | 1.10(0.59-2.04) | 0.77 | 1.05(0.59-1.86) | 0.87 |
|  |  |  |  |  |

P1 represented for statistical significance for PS, P2 represented for statistical significance for GS.

H represented the group with higher PKM/PKLR expression (upper 50% of all cases), L represented the group with lower PKM/PKLR expression (lower 50% ot all cases).

Abbreviation: GS, grafts’ survival; PS, patients’ survival.

Table S15 Positive co-expressed links in hepatocytes with PKLR perturbation

| row | column | cor | p | interaction |
| --- | --- | --- | --- | --- |
| C00141 | C00212 | -0.75184046 | 0.012143916 | inhibted |
| C00141 | C00164 | -0.641305748 | 0.045669951 | inhibted |
| C00164 | C00058 | 0.67687995 | 0.031573604 | actived |
| C00212 | C00137 | 0.820986077 | 0.003597887 | actived |
| C00058 | C00137 | 0.735421235 | 0.015354027 | actived |
| C00058 | C01879 | 0.801632548 | 0.005291188 | actived |
| C00137 | C01879 | 0.686666374 | 0.028291648 | actived |
| C00058 | C19654 | 0.775789179 | 0.008350466 | actived |
| C00137 | C19654 | 0.698347373 | 0.024689164 | actived |
| C00058 | C17714 | 0.650287015 | 0.041777699 | actived |
| C00137 | C17714 | 0.729325495 | 0.016683008 | actived |
| C00164 | C01595 | 0.644192502 | 0.044393529 | actived |
| C00058 | C01595 | 0.947459205 | 3.13E-05 | actived |
| C00137 | C01595 | 0.67183762 | 0.033361552 | actived |
| C01879 | C01595 | 0.872960472 | 0.000974858 | actived |
| C19654 | C01595 | 0.731696937 | 0.016156857 | actived |
| C00058 | C01530 | 0.864101004 | 0.001262412 | actived |
| C00137 | C01530 | 0.797908255 | 0.005672476 | actived |
| C01879 | C01530 | 0.687451267 | 0.028038987 | actived |
| C19654 | C01530 | 0.919597457 | 0.000165778 | actived |
| C17714 | C01530 | 0.652741917 | 0.040753969 | actived |
| C01595 | C01530 | 0.7732565 | 0.008705352 | actived |
| C00141 | C08278 | 0.860494927 | 0.001395461 | actived |
| C00212 | C08278 | -0.641623191 | 0.045528403 | inhibted |
| C00164 | C08278 | -0.774747419 | 0.008495177 | inhibted |
| C00141 | C08281 | 0.815391056 | 0.004040071 | actived |
| C00164 | C08281 | -0.71097493 | 0.02116347 | inhibted |
| C00058 | C08281 | -0.676218453 | 0.031804358 | inhibted |
| C00137 | C08281 | -0.688769144 | 0.027618232 | inhibted |
| C17714 | C08281 | -0.68112571 | 0.030119585 | inhibted |
| C08278 | C08281 | 0.837994129 | 0.002466455 | actived |
| C00212 | C04299 | 0.645484596 | 0.043830034 | actived |
| C00058 | C04299 | 0.742215405 | 0.013961778 | actived |
| C00137 | C04299 | 0.663913016 | 0.036308328 | actived |
| C01879 | C04299 | 0.665255928 | 0.035797043 | actived |
| C19654 | C04299 | 0.944114178 | 3.99E-05 | actived |
| C01595 | C04299 | 0.794721831 | 0.0060139 | actived |
| C01530 | C04299 | 0.849906117 | 0.001844947 | actived |
| C00141 | C06524 | -0.735857626 | 0.015261814 | inhibted |
| C00164 | C06524 | 0.810041415 | 0.004498028 | actived |
| C00058 | C06524 | 0.900861399 | 0.000374389 | actived |
| C00137 | C06524 | 0.744226455 | 0.013567219 | actived |
| C01879 | C06524 | 0.698819612 | 0.024550529 | actived |
| C19654 | C06524 | 0.800041653 | 0.00545175 | actived |
| C17714 | C06524 | 0.777079649 | 0.008173634 | actived |
| C01595 | C06524 | 0.881241814 | 0.000752243 | actived |
| C01530 | C06524 | 0.808688115 | 0.004619516 | actived |
| C08278 | C06524 | -0.669654555 | 0.034156504 | inhibted |
| C08281 | C06524 | -0.769121191 | 0.009307502 | inhibted |
| C04299 | C06524 | 0.773203693 | 0.008712863 | actived |
| C00141 | C00612 | 0.69802628 | 0.024783733 | actived |
| C00164 | C00612 | -0.663852454 | 0.036331502 | inhibted |
| C17714 | C00612 | -0.773777383 | 0.00863151 | inhibted |
| C08281 | C00612 | 0.855429711 | 0.001599153 | actived |
| C06524 | C00612 | -0.704614647 | 0.022892577 | inhibted |
| C00164 | C01107 | -0.786451299 | 0.006967999 | inhibted |
| C00058 | C01107 | -0.783082873 | 0.007385698 | inhibted |
| C19654 | C01107 | -0.705297818 | 0.022702348 | inhibted |
| C01595 | C01107 | -0.783362408 | 0.007350376 | inhibted |
| C01530 | C01107 | -0.674094389 | 0.032553057 | inhibted |
| C08278 | C01107 | 0.657324103 | 0.038888528 | actived |
| C04299 | C01107 | -0.694654461 | 0.025791869 | inhibted |
| C06524 | C01107 | -0.846561884 | 0.002006413 | inhibted |
| C00212 | C12621 | -0.633589517 | 0.049201675 | inhibted |
| C00137 | C12621 | -0.643286356 | 0.044791589 | inhibted |
| C19654 | C12621 | -0.850250967 | 0.00182885 | inhibted |
| C17714 | C12621 | -0.651529635 | 0.041257375 | inhibted |
| C01530 | C12621 | -0.822108229 | 0.003513574 | inhibted |
| C04299 | C12621 | -0.819411524 | 0.003718625 | inhibted |
| C06524 | C12621 | -0.698783524 | 0.024561105 | inhibted |
| C00058 | C00184 | 0.643110595 | 0.044869075 | actived |
| C00137 | C00184 | 0.664920745 | 0.035924198 | actived |
| C19654 | C00184 | 0.70320716 | 0.023287946 | actived |
| C17714 | C00184 | 0.800110992 | 0.005444681 | actived |
| C06524 | C00184 | 0.81742158 | 0.003875339 | actived |
| C12621 | C00184 | -0.773352188 | 0.008691754 | inhibted |
| C00058 | C19670 | 0.646032702 | 0.043592455 | actived |
| C00137 | C19670 | 0.739327105 | 0.014542372 | actived |
| C19654 | C19670 | 0.741911819 | 0.014022028 | actived |
| C01530 | C19670 | 0.850498742 | 0.001817348 | actived |
| C04299 | C19670 | 0.74692865 | 0.013049427 | actived |
| C06524 | C19670 | 0.650558739 | 0.041663545 | actived |
| C12621 | C19670 | -0.633517404 | 0.049235512 | inhibted |
| C00141 | C06425 | 0.685225625 | 0.028759471 | actived |
| C00212 | C06425 | -0.72701548 | 0.01720687 | inhibted |
| C00058 | C06425 | -0.640453606 | 0.046051377 | inhibted |
| C00137 | C06425 | -0.640602407 | 0.04598462 | inhibted |
| C01879 | C06425 | -0.656058181 | 0.039398018 | inhibted |
| C19654 | C06425 | -0.639681501 | 0.046398812 | inhibted |
| C17714 | C06425 | -0.638980193 | 0.046715903 | inhibted |
| C01595 | C06425 | -0.739109142 | 0.014586858 | inhibted |
| C04299 | C06425 | -0.71800919 | 0.019358532 | inhibted |
| C06524 | C06425 | -0.821867928 | 0.003531508 | inhibted |
| C01107 | C06425 | 0.685930606 | 0.028529903 | actived |
| C12621 | C06425 | 0.759019957 | 0.010900955 | actived |
| C00184 | C06425 | -0.869357829 | 0.001085299 | inhibted |
| C00137 | C06866 | 0.828037312 | 0.003091451 | actived |
| C08281 | C06866 | -0.661937441 | 0.037069425 | inhibted |
| C19670 | C06866 | 0.690482492 | 0.027077697 | actived |
| C00137 | C15589 | -0.648673821 | 0.042459746 | inhibted |
| C19654 | C15589 | -0.783068307 | 0.007387542 | inhibted |
| C17714 | C15589 | -0.65585369 | 0.039480738 | inhibted |
| C01530 | C15589 | -0.848227763 | 0.001924756 | inhibted |
| C04299 | C15589 | -0.738787642 | 0.014652649 | inhibted |
| C12621 | C15589 | 0.929366554 | 9.99E-05 | actived |
| C19670 | C15589 | -0.776566162 | 0.008243676 | inhibted |
| C00141 | C05552 | 0.78145433 | 0.007593875 | actived |
| C00212 | C05552 | -0.738444436 | 0.014723111 | inhibted |
| C00164 | C05552 | -0.723283519 | 0.018077164 | inhibted |
| C00058 | C05552 | -0.79972004 | 0.005484626 | inhibted |
| C00137 | C05552 | -0.862478522 | 0.001321078 | inhibted |
| C01879 | C05552 | -0.71366456 | 0.020460187 | inhibted |
| C17714 | C05552 | -0.840911423 | 0.002302082 | inhibted |
| C01595 | C05552 | -0.74866533 | 0.012724044 | inhibted |
| C01530 | C05552 | -0.738397709 | 0.014732722 | inhibted |
| C08278 | C05552 | 0.749626173 | 0.012546485 | actived |
| C08281 | C05552 | 0.921979318 | 0.000147423 | actived |
| C06524 | C05552 | -0.890244968 | 0.000555006 | inhibted |
| C00612 | C05552 | 0.878350144 | 0.000825229 | actived |
| C00184 | C05552 | -0.734590025 | 0.015530742 | inhibted |
| C19670 | C05552 | -0.651035125 | 0.041463917 | inhibted |
| C06425 | C05552 | 0.725066389 | 0.01765768 | actived |
| C06866 | C05552 | -0.685993465 | 0.028509495 | inhibted |
| C00141 | C08842 | -0.638075808 | 0.047126946 | inhibted |
| C00164 | C08842 | 0.676288108 | 0.031780007 | actived |
| C00058 | C08842 | 0.638020683 | 0.047152078 | actived |
| C01879 | C08842 | 0.794789257 | 0.006006528 | actived |
| C01595 | C08842 | 0.651432245 | 0.041297997 | actived |
| C08278 | C08842 | -0.671814078 | 0.033370057 | inhibted |
| C08281 | C08842 | -0.705554135 | 0.022631259 | inhibted |
| C00612 | C08842 | -0.695837039 | 0.02543515 | inhibted |
| C19670 | C08842 | 0.759458085 | 0.01082813 | actived |
| C05552 | C08842 | -0.745005107 | 0.013416568 | inhibted |
| C00141 | C00521 | 0.840033903 | 0.002350667 | actived |
| C00212 | C00521 | -0.786613209 | 0.006948354 | inhibted |
| C00164 | C00521 | -0.676982225 | 0.031538029 | inhibted |
| C00137 | C00521 | -0.669034567 | 0.034384589 | inhibted |
| C08278 | C00521 | 0.9168056 | 0.000189376 | actived |
| C08281 | C00521 | 0.891562281 | 0.000529708 | actived |
| C06524 | C00521 | -0.691956126 | 0.026618616 | inhibted |
| C00612 | C00521 | 0.657505867 | 0.038815741 | actived |
| C05552 | C00521 | 0.824285537 | 0.003354041 | actived |
| C00212 | C14833 | 0.704736196 | 0.022858652 | actived |
| C00058 | C14833 | 0.801078542 | 0.005346713 | actived |
| C00137 | C14833 | 0.876646992 | 0.000870558 | actived |
| C01879 | C14833 | 0.923727089 | 0.000134941 | actived |
| C01595 | C14833 | 0.813072318 | 0.004234251 | actived |
| C01530 | C14833 | 0.73799826 | 0.014815065 | actived |
| C08281 | C14833 | -0.677938896 | 0.03120658 | inhibted |
| C06524 | C14833 | 0.71633563 | 0.019777903 | actived |
| C00612 | C14833 | -0.651251086 | 0.041373631 | inhibted |
| C19670 | C14833 | 0.722360316 | 0.018297073 | actived |
| C06866 | C14833 | 0.685721714 | 0.028597795 | actived |
| C05552 | C14833 | -0.833338091 | 0.00274609 | inhibted |
| C08842 | C14833 | 0.829293396 | 0.003006941 | actived |
| C00521 | C14833 | -0.638373015 | 0.0469916 | inhibted |
| C17714 | C06126 | -0.747510265 | 0.012939815 | inhibted |
| C12621 | C06126 | 0.785652422 | 0.007065506 | actived |
| C00184 | C06126 | -0.642880486 | 0.044970656 | inhibted |
| C06425 | C06126 | 0.663886173 | 0.036318598 | actived |
| C15589 | C06126 | 0.781345357 | 0.007607952 | actived |
| C00058 | C01290 | -0.82411314 | 0.003366479 | inhibted |
| C00137 | C01290 | -0.868610797 | 0.001109291 | inhibted |
| C17714 | C01290 | -0.77331202 | 0.00869746 | inhibted |
| C01595 | C01290 | -0.701893052 | 0.0236613 | inhibted |
| C01530 | C01290 | -0.758787618 | 0.010939714 | inhibted |
| C08281 | C01290 | 0.736180405 | 0.015193857 | actived |
| C06524 | C01290 | -0.773151904 | 0.008720233 | inhibted |
| C00612 | C01290 | 0.687175401 | 0.028127613 | actived |
| C19670 | C01290 | -0.715571041 | 0.019971573 | inhibted |
| C06866 | C01290 | -0.82418986 | 0.00336094 | inhibted |
| C05552 | C01290 | 0.845722981 | 0.002048467 | actived |
| C08842 | C01290 | -0.672651138 | 0.033068554 | inhibted |
| C14833 | C01290 | -0.800797595 | 0.005375029 | inhibted |
| C00141 | C06834 | 0.870582154 | 0.001046796 | actived |
| C00212 | C06834 | -0.6564426 | 0.03924283 | inhibted |
| C00164 | C06834 | -0.639806911 | 0.04634226 | inhibted |
| C01530 | C06834 | -0.634128289 | 0.04894936 | inhibted |
| C08278 | C06834 | 0.940537548 | 5.09E-05 | actived |
| C08281 | C06834 | 0.799082507 | 0.005550213 | actived |
| C06524 | C06834 | -0.71215022 | 0.020854136 | inhibted |
| C01107 | C06834 | 0.721571572 | 0.018486417 | actived |
| C05552 | C06834 | 0.756144117 | 0.011387545 | actived |
| C00521 | C06834 | 0.906053319 | 0.000303866 | actived |
| C00141 | C06575 | 0.637923836 | 0.047196253 | actived |
| C01879 | C06575 | -0.650042529 | 0.04188059 | inhibted |
| C17714 | C06575 | -0.724079769 | 0.017888973 | inhibted |
| C01595 | C06575 | -0.632766221 | 0.049588907 | inhibted |
| C08281 | C06575 | 0.655059611 | 0.039803065 | actived |
| C06524 | C06575 | -0.738784835 | 0.014653225 | inhibted |
| C00612 | C06575 | 0.82957041 | 0.002988529 | actived |
| C00184 | C06575 | -0.741497852 | 0.014104477 | inhibted |
| C06425 | C06575 | 0.87586558 | 0.000891951 | actived |
| C05552 | C06575 | 0.794021642 | 0.00609084 | actived |
| C14833 | C06575 | -0.64755285 | 0.042938059 | inhibted |
| C00137 | C05579 | 0.671641663 | 0.033432391 | actived |
| C19654 | C05579 | 0.709969771 | 0.021430526 | actived |
| C17714 | C05579 | 0.828975884 | 0.003028145 | actived |
| C01530 | C05579 | 0.751202271 | 0.012258999 | actived |
| C06524 | C05579 | 0.650908864 | 0.041516764 | actived |
| C12621 | C05579 | -0.767318281 | 0.009578998 | inhibted |
| C00184 | C05579 | 0.730140695 | 0.016500816 | actived |
| C15589 | C05579 | -0.784300722 | 0.007232687 | inhibted |
| C06126 | C05579 | -0.666499573 | 0.035327916 | inhibted |
| C01290 | C05579 | -0.634831566 | 0.048621305 | inhibted |
| C00164 | C00157 | 0.766714994 | 0.009671079 | actived |
| C00058 | C00157 | 0.706020176 | 0.022502395 | actived |
| C00137 | C00157 | 0.7598736 | 0.01075938 | actived |
| C19654 | C00157 | 0.644927112 | 0.044072567 | actived |
| C17714 | C00157 | 0.677987012 | 0.031189973 | actived |
| C08281 | C00157 | -0.696238841 | 0.025314723 | inhibted |
| C06524 | C00157 | 0.756769412 | 0.011280474 | actived |
| C00184 | C00157 | 0.697214998 | 0.025023781 | actived |
| C05552 | C00157 | -0.750221612 | 0.012437326 | inhibted |
| C00521 | C00157 | -0.646262449 | 0.043493127 | inhibted |
| C01290 | C00157 | -0.780864606 | 0.007670274 | inhibted |
| C05579 | C00157 | 0.721119866 | 0.018595462 | actived |
| C00141 | C00350 | 0.882461133 | 0.000722926 | actived |
| C00212 | C00350 | -0.755772635 | 0.011451492 | inhibted |
| C00164 | C00350 | -0.777804799 | 0.008075438 | inhibted |
| C00058 | C00350 | -0.742879524 | 0.013830606 | inhibted |
| C00137 | C00350 | -0.67222851 | 0.03322055 | inhibted |
| C01879 | C00350 | -0.735683825 | 0.015298494 | inhibted |
| C19654 | C00350 | -0.721888866 | 0.018410086 | inhibted |
| C17714 | C00350 | -0.655836083 | 0.039487866 | inhibted |
| C01595 | C00350 | -0.779889623 | 0.007797776 | inhibted |
| C01530 | C00350 | -0.737796242 | 0.014856831 | inhibted |
| C08278 | C00350 | 0.826886592 | 0.003170359 | actived |
| C08281 | C00350 | 0.758410331 | 0.011002859 | actived |
| C04299 | C00350 | -0.74686532 | 0.013061401 | inhibted |
| C06524 | C00350 | -0.910476359 | 0.000251937 | inhibted |
| C00612 | C00350 | 0.682108176 | 0.02978975 | actived |
| C01107 | C00350 | 0.842411793 | 0.002220698 | actived |
| C12621 | C00350 | 0.732966981 | 0.015879875 | actived |
| C00184 | C00350 | -0.749417935 | 0.012584818 | inhibted |
| C19670 | C00350 | -0.635378933 | 0.048366993 | inhibted |
| C06425 | C00350 | 0.860367549 | 0.001400339 | actived |
| C05552 | C00350 | 0.857299285 | 0.001521621 | actived |
| C08842 | C00350 | -0.664982686 | 0.035900677 | inhibted |
| C00521 | C00350 | 0.816908937 | 0.003916465 | actived |
| C14833 | C00350 | -0.687306583 | 0.028085445 | inhibted |
| C06126 | C00350 | 0.67889899 | 0.030876336 | actived |
| C06834 | C00350 | 0.879309075 | 0.000800478 | actived |
| C06575 | C00350 | 0.774850677 | 0.008480755 | actived |
| C00164 | C04230 | 0.644652038 | 0.044192568 | actived |
| C00058 | C04230 | 0.870299897 | 0.001055583 | actived |
| C00137 | C04230 | 0.661232938 | 0.037343423 | actived |
| C01879 | C04230 | 0.956198236 | 1.53E-05 | actived |
| C01595 | C04230 | 0.929462178 | 9.94E-05 | actived |
| C01530 | C04230 | 0.712217108 | 0.020836626 | actived |
| C04299 | C04230 | 0.694486352 | 0.025842855 | actived |
| C06524 | C04230 | 0.833426876 | 0.002740554 | actived |
| C00612 | C04230 | -0.638881179 | 0.046760787 | inhibted |
| C01107 | C04230 | -0.770348873 | 0.009125765 | inhibted |
| C06425 | C04230 | -0.768514634 | 0.009398228 | inhibted |
| C05552 | C04230 | -0.782366053 | 0.007476824 | inhibted |
| C08842 | C04230 | 0.75684869 | 0.01126695 | actived |
| C14833 | C04230 | 0.871522073 | 0.00101792 | actived |
| C06575 | C04230 | -0.769019283 | 0.009322701 | inhibted |
| C00350 | C04230 | -0.828352535 | 0.003070084 | inhibted |
| C17714 | C04598 | 0.964971691 | 6.31E-06 | actived |
| C00612 | C04598 | -0.73347516 | 0.015769979 | inhibted |
| C00184 | C04598 | 0.719536888 | 0.018981122 | actived |
| C05552 | C04598 | -0.726376354 | 0.017353803 | inhibted |
| C06126 | C04598 | -0.642388766 | 0.045188239 | inhibted |
| C01290 | C04598 | -0.686512859 | 0.028341246 | inhibted |
| C06575 | C04598 | -0.665328543 | 0.035769536 | inhibted |
| C05579 | C04598 | 0.777180729 | 0.008159896 | actived |
| C00141 | C04317 | -0.65737093 | 0.038869767 | inhibted |
| C08278 | C04317 | -0.685794655 | 0.028574076 | inhibted |
| C08281 | C04317 | -0.678558445 | 0.0309932 | inhibted |
| C00612 | C04317 | -0.80288434 | 0.005167243 | inhibted |
| C05552 | C04317 | -0.705745743 | 0.022578216 | inhibted |
| C08842 | C04317 | 0.703841251 | 0.023109251 | actived |
| C00141 | C02277 | -0.742317605 | 0.013941535 | inhibted |
| C00212 | C02277 | 0.76042131 | 0.010669227 | actived |
| C00164 | C02277 | 0.676660969 | 0.031649866 | actived |
| C00058 | C02277 | 0.840689368 | 0.002314307 | actived |
| C00137 | C02277 | 0.775845365 | 0.008342711 | actived |
| C01879 | C02277 | 0.898153853 | 0.000415591 | actived |
| C19654 | C02277 | 0.713403946 | 0.020527615 | actived |
| C01595 | C02277 | 0.917462613 | 0.000183613 | actived |
| C01530 | C02277 | 0.75669188 | 0.011293711 | actived |
| C08281 | C02277 | -0.697198287 | 0.025028743 | inhibted |
| C04299 | C02277 | 0.802888395 | 0.005166845 | actived |
| C06524 | C02277 | 0.899547298 | 0.000393992 | actived |
| C00612 | C02277 | -0.655434263 | 0.039650769 | inhibted |
| C01107 | C02277 | -0.750844602 | 0.01232383 | inhibted |
| C12621 | C02277 | -0.691814595 | 0.026662474 | inhibted |
| C00184 | C02277 | 0.711356143 | 0.021062791 | actived |
| C19670 | C02277 | 0.644163175 | 0.044406375 | actived |
| C06425 | C02277 | -0.891702765 | 0.000527061 | inhibted |
| C05552 | C02277 | -0.853116044 | 0.001699018 | inhibted |
| C08842 | C02277 | 0.709056639 | 0.021675138 | actived |
| C00521 | C02277 | -0.732531526 | 0.015974467 | inhibted |
| C14833 | C02277 | 0.864774659 | 0.001238619 | actived |
| C01290 | C02277 | -0.657235167 | 0.038924176 | inhibted |
| C06834 | C02277 | -0.704594069 | 0.022898324 | inhibted |
| C06575 | C02277 | -0.806018839 | 0.004865988 | inhibted |
| C00350 | C02277 | -0.91871081 | 0.000173022 | inhibted |
| C04230 | C02277 | 0.944836538 | 3.79E-05 | actived |
| C00141 | C04692 | 0.87284515 | 0.00097826 | actived |
| C00212 | C04692 | -0.723958052 | 0.017917652 | inhibted |
| C00164 | C04692 | -0.796766499 | 0.005793184 | inhibted |
| C00058 | C04692 | -0.750701366 | 0.01234986 | inhibted |
| C00137 | C04692 | -0.765763771 | 0.009817531 | inhibted |
| C01879 | C04692 | -0.6919438 | 0.026622434 | inhibted |
| C17714 | C04692 | -0.765733991 | 0.009822141 | inhibted |
| C01595 | C04692 | -0.71448033 | 0.020250113 | inhibted |
| C01530 | C04692 | -0.724987075 | 0.017676196 | inhibted |
| C08278 | C04692 | 0.856868471 | 0.00153924 | actived |
| C08281 | C04692 | 0.919721368 | 0.000164784 | actived |
| C06524 | C04692 | -0.883543495 | 0.000697609 | inhibted |
| C00612 | C04692 | 0.86050022 | 0.001395258 | actived |
| C01107 | C04692 | 0.683241126 | 0.029412461 | actived |
| C00184 | C04692 | -0.648600458 | 0.04249094 | inhibted |
| C19670 | C04692 | -0.707344732 | 0.022138905 | inhibted |
| C06425 | C04692 | 0.680395757 | 0.030366251 | actived |
| C05552 | C04692 | 0.965436556 | 5.99E-06 | actived |
| C08842 | C04692 | -0.812410486 | 0.004290879 | inhibted |
| C00521 | C04692 | 0.841356208 | 0.002277735 | actived |
| C14833 | C04692 | -0.767379806 | 0.009569642 | inhibted |
| C01290 | C04692 | 0.762074669 | 0.010400309 | actived |
| C06834 | C04692 | 0.831379822 | 0.002870256 | actived |
| C06575 | C04692 | 0.732490316 | 0.015983439 | actived |
| C00157 | C04692 | -0.67856909 | 0.030989542 | inhibted |
| C00350 | C04692 | 0.908362699 | 0.000275862 | actived |
| C04230 | C04692 | -0.767946997 | 0.009483696 | inhibted |
| C04317 | C04692 | -0.77323964 | 0.00870775 | inhibted |
| C02277 | C04692 | -0.834475617 | 0.002675766 | inhibted |
| C00212 | C03577 | -0.741899084 | 0.014024559 | inhibted |
| C00137 | C03577 | -0.651168435 | 0.041408169 | inhibted |
| C19654 | C03577 | -0.784607611 | 0.007194487 | inhibted |
| C01530 | C03577 | -0.661602722 | 0.037199436 | inhibted |
| C04299 | C03577 | -0.812444357 | 0.004287968 | inhibted |
| C06524 | C03577 | -0.767843874 | 0.009499281 | inhibted |
| C01107 | C03577 | 0.653787935 | 0.040322938 | actived |
| C12621 | C03577 | 0.897593712 | 0.000424513 | actived |
| C00184 | C03577 | -0.86251904 | 0.001319589 | inhibted |
| C06425 | C03577 | 0.921307793 | 0.000152438 | actived |
| C15589 | C03577 | 0.729054786 | 0.016743818 | actived |
| C06126 | C03577 | 0.762454962 | 0.010339136 | actived |
| C06575 | C03577 | 0.684629626 | 0.028954531 | actived |
| C05579 | C03577 | -0.652757253 | 0.040747626 | inhibted |
| C00350 | C03577 | 0.809453009 | 0.004550566 | actived |
| C02277 | C03577 | -0.782016364 | 0.007521567 | inhibted |
| C00141 | C07310 | 0.848293147 | 0.001921601 | actived |
| C00164 | C07310 | -0.813491961 | 0.004198624 | inhibted |
| C08278 | C07310 | 0.996282142 | 8.32E-10 | actived |
| C08281 | C07310 | 0.830542404 | 0.002924567 | actived |
| C06524 | C07310 | -0.685704732 | 0.028603319 | inhibted |
| C01107 | C07310 | 0.681957465 | 0.029840186 | actived |
| C05552 | C07310 | 0.74116081 | 0.014171855 | actived |
| C08842 | C07310 | -0.686522317 | 0.028338189 | inhibted |
| C00521 | C07310 | 0.896486825 | 0.000442555 | actived |
| C06834 | C07310 | 0.920257783 | 0.00016053 | actived |
| C00350 | C07310 | 0.823537878 | 0.003408223 | actived |
| C04317 | C07310 | -0.681194672 | 0.030096352 | inhibted |
| C04692 | C07310 | 0.856028423 | 0.00157402 | actived |
| C00058 | C07598 | 0.685109325 | 0.028797463 | actived |
| C00137 | C07598 | 0.852354991 | 0.001732832 | actived |
| C17714 | C07598 | 0.9594273 | 1.13E-05 | actived |
| C01530 | C07598 | 0.708135305 | 0.021923893 | actived |
| C08281 | C07598 | -0.761852692 | 0.010436133 | inhibted |
| C06524 | C07598 | 0.77339462 | 0.008685728 | actived |
| C00612 | C07598 | -0.767351579 | 0.009573934 | inhibted |
| C00184 | C07598 | 0.763319218 | 0.010201056 | actived |
| C06866 | C07598 | 0.720284974 | 0.018798183 | actived |
| C05552 | C07598 | -0.892534508 | 0.000511586 | inhibted |
| C14833 | C07598 | 0.658809345 | 0.038296453 | actived |
| C01290 | C07598 | -0.866485379 | 0.001179664 | inhibted |
| C06575 | C07598 | -0.656507391 | 0.039216715 | inhibted |
| C05579 | C07598 | 0.834790367 | 0.002656539 | actived |
| C00157 | C07598 | 0.802391765 | 0.005215765 | actived |
| C00350 | C07598 | -0.649763606 | 0.04199818 | inhibted |
| C04598 | C07598 | 0.906156371 | 0.000302574 | actived |
| C04317 | C07598 | 0.64933582 | 0.04217896 | actived |
| C04692 | C07598 | -0.807486417 | 0.004729345 | inhibted |
| C00212 | C07934 | 0.76873926 | 0.009364557 | actived |
| C00058 | C07934 | 0.749745158 | 0.012524618 | actived |
| C00137 | C07934 | 0.779838263 | 0.007804534 | actived |
| C01879 | C07934 | 0.87441673 | 0.000932628 | actived |
| C19654 | C07934 | 0.76522888 | 0.009900566 | actived |
| C01595 | C07934 | 0.816173674 | 0.003975996 | actived |
| C01530 | C07934 | 0.816975107 | 0.003911139 | actived |
| C04299 | C07934 | 0.852533159 | 0.001724873 | actived |
| C06524 | C07934 | 0.680195723 | 0.030434086 | actived |
| C12621 | C07934 | -0.649629605 | 0.042054752 | inhibted |
| C19670 | C07934 | 0.741013329 | 0.014201408 | actived |
| C06425 | C07934 | -0.637842666 | 0.047233299 | inhibted |
| C05552 | C07934 | -0.665403226 | 0.035741261 | inhibted |
| C08842 | C07934 | 0.674966305 | 0.032244288 | actived |
| C00521 | C07934 | -0.669920971 | 0.03405881 | inhibted |
| C14833 | C07934 | 0.855439363 | 0.001598746 | actived |
| C00350 | C07934 | -0.730949615 | 0.0163214 | inhibted |
| C04230 | C07934 | 0.790031784 | 0.006542683 | actived |
| C02277 | C07934 | 0.85725368 | 0.001523479 | actived |
| C04692 | C07934 | -0.651171429 | 0.041406918 | inhibted |
| C00164 | C07468 | 0.647615481 | 0.04291124 | actived |
| C00058 | C07468 | 0.872407676 | 0.000991247 | actived |
| C00137 | C07468 | 0.654545256 | 0.040012791 | actived |
| C01879 | C07468 | 0.960744618 | 9.91E-06 | actived |
| C01595 | C07468 | 0.9433323 | 4.21E-05 | actived |
| C01530 | C07468 | 0.692706744 | 0.026386838 | actived |
| C04299 | C07468 | 0.709496993 | 0.021556936 | actived |
| C06524 | C07468 | 0.820510163 | 0.003634079 | actived |
| C01107 | C07468 | -0.768372443 | 0.009419586 | inhibted |
| C06425 | C07468 | -0.772801136 | 0.00877027 | inhibted |
| C05552 | C07468 | -0.750475737 | 0.012390941 | inhibted |
| C08842 | C07468 | 0.721374403 | 0.018533961 | actived |
| C14833 | C07468 | 0.861833872 | 0.001344928 | actived |
| C06575 | C07468 | -0.749623327 | 0.012547008 | inhibted |
| C00350 | C07468 | -0.808065953 | 0.004676148 | inhibted |
| C04230 | C07468 | 0.992523668 | 1.35E-08 | actived |
| C02277 | C07468 | 0.943930459 | 4.04E-05 | actived |
| C04692 | C07468 | -0.725605019 | 0.017532289 | inhibted |
| C07934 | C07468 | 0.807936217 | 0.004688019 | actived |
| C00212 | C08052 | -0.637286559 | 0.047487627 | inhibted |
| C00164 | C08052 | -0.672534206 | 0.03311056 | inhibted |
| C00058 | C08052 | -0.777738082 | 0.008084438 | inhibted |
| C00137 | C08052 | -0.803831998 | 0.0050748 | inhibted |
| C01879 | C08052 | -0.653081219 | 0.040613815 | inhibted |
| C19654 | C08052 | -0.775473556 | 0.008394124 | inhibted |
| C17714 | C08052 | -0.833496303 | 0.00273623 | inhibted |
| C01595 | C08052 | -0.661662567 | 0.037176168 | inhibted |
| C01530 | C08052 | -0.876239785 | 0.000881659 | inhibted |
| C04299 | C08052 | -0.701015408 | 0.023912929 | inhibted |
| C06524 | C08052 | -0.84589106 | 0.002039991 | inhibted |
| C01107 | C08052 | 0.707700375 | 0.022042002 | actived |
| C12621 | C08052 | 0.817225863 | 0.003891003 | actived |
| C00184 | C08052 | -0.697491889 | 0.024941674 | inhibted |
| C19670 | C08052 | -0.791004448 | 0.006430407 | inhibted |
| C06425 | C08052 | 0.677630019 | 0.031313334 | actived |
| C15589 | C08052 | 0.846898014 | 0.001989739 | actived |
| C05552 | C08052 | 0.834010469 | 0.002704364 | actived |
| C14833 | C08052 | -0.716130218 | 0.019829806 | inhibted |
| C06126 | C08052 | 0.768150334 | 0.009453017 | actived |
| C01290 | C08052 | 0.740099297 | 0.014385529 | actived |
| C06834 | C08052 | 0.664156519 | 0.036215255 | actived |
| C06575 | C08052 | 0.635810919 | 0.048166916 | actived |
| C05579 | C08052 | -0.857661178 | 0.001506934 | inhibted |
| C00157 | C08052 | -0.693142356 | 0.026252964 | inhibted |
| C00350 | C08052 | 0.822214238 | 0.003505683 | actived |
| C04230 | C08052 | -0.731732143 | 0.016149134 | inhibted |
| C04598 | C08052 | -0.692533093 | 0.026440335 | inhibted |
| C02277 | C08052 | -0.769659989 | 0.009227431 | inhibted |
| C04692 | C08052 | 0.830284347 | 0.002941452 | actived |
| C03577 | C08052 | 0.752518568 | 0.012022467 | actived |
| C07598 | C08052 | -0.846233056 | 0.002022822 | inhibted |
| C07934 | C08052 | -0.647468478 | 0.042974206 | inhibted |
| C07468 | C08052 | -0.6928919 | 0.026329878 | inhibted |
| C00164 | C07363 | 0.704211284 | 0.023005408 | actived |
| C00058 | C07363 | 0.746508829 | 0.01312895 | actived |
| C19654 | C07363 | 0.92230686 | 0.000145021 | actived |
| C01595 | C07363 | 0.741375534 | 0.014128904 | actived |
| C01530 | C07363 | 0.791164122 | 0.006412108 | actived |
| C04299 | C07363 | 0.893437638 | 0.000495162 | actived |
| C06524 | C07363 | 0.862044748 | 0.001337092 | actived |
| C01107 | C07363 | -0.879589251 | 0.000793349 | inhibted |
| C12621 | C07363 | -0.817763714 | 0.003848064 | inhibted |
| C00184 | C07363 | 0.765575158 | 0.009846755 | actived |
| C06425 | C07363 | -0.762613005 | 0.010313788 | inhibted |
| C15589 | C07363 | -0.675038181 | 0.032218924 | inhibted |
| C06834 | C07363 | -0.652785428 | 0.040735977 | inhibted |
| C05579 | C07363 | 0.659785825 | 0.037910522 | actived |
| C00157 | C07363 | 0.640740736 | 0.045922618 | actived |
| C00350 | C07363 | -0.831171312 | 0.002883711 | inhibted |
| C04230 | C07363 | 0.650767482 | 0.041575993 | actived |
| C02277 | C07363 | 0.757648368 | 0.011131165 | actived |
| C04692 | C07363 | -0.639964859 | 0.046271103 | inhibted |
| C03577 | C07363 | -0.859515321 | 0.001433295 | inhibted |
| C07934 | C07363 | 0.654401275 | 0.040071632 | actived |
| C07468 | C07363 | 0.662270651 | 0.036940307 | actived |
| C08052 | C07363 | -0.767756625 | 0.009512482 | inhibted |
| C00141 | C07372 | -0.81539257 | 0.004039946 | inhibted |
| C00164 | C07372 | 0.780105103 | 0.007769469 | actived |
| C00058 | C07372 | 0.766138417 | 0.009759665 | actived |
| C19654 | C07372 | 0.712843227 | 0.020673209 | actived |
| C01595 | C07372 | 0.743040107 | 0.013799019 | actived |
| C01530 | C07372 | 0.726536703 | 0.017316858 | actived |
| C08278 | C07372 | -0.811802133 | 0.004343407 | inhibted |
| C08281 | C07372 | -0.823963099 | 0.003377331 | inhibted |
| C04299 | C07372 | 0.650512236 | 0.041683067 | actived |
| C06524 | C07372 | 0.8526838 | 0.001718164 | actived |
| C01107 | C07372 | -0.768125912 | 0.009456698 | inhibted |
| C19670 | C07372 | 0.638114529 | 0.047109298 | actived |
| C05552 | C07372 | -0.768636436 | 0.009379959 | inhibted |
| C08842 | C07372 | 0.656085885 | 0.03938682 | actived |
| C00521 | C07372 | -0.741801869 | 0.014043894 | inhibted |
| C01290 | C07372 | -0.658419421 | 0.0384513 | inhibted |
| C06834 | C07372 | -0.791726938 | 0.0063479 | inhibted |
| C00350 | C07372 | -0.809722529 | 0.004526446 | inhibted |
| C02277 | C07372 | 0.70466523 | 0.022878455 | actived |
| C04692 | C07372 | -0.84805184 | 0.001933264 | inhibted |
| C07310 | C07372 | -0.836821272 | 0.002534864 | inhibted |
| C08052 | C07372 | -0.638786395 | 0.046803781 | inhibted |
| C07363 | C07372 | 0.737716575 | 0.014873324 | actived |
| C00164 | C12811 | 0.660503173 | 0.037628684 | actived |
| C00058 | C12811 | 0.954509272 | 1.77E-05 | actived |
| C01879 | C12811 | 0.805209743 | 0.004942516 | actived |
| C19654 | C12811 | 0.739228052 | 0.014562577 | actived |
| C01595 | C12811 | 0.970392957 | 3.24E-06 | actived |
| C01530 | C12811 | 0.791448739 | 0.00637958 | actived |
| C04299 | C12811 | 0.771729745 | 0.008924359 | actived |
| C06524 | C12811 | 0.867989126 | 0.00112955 | actived |
| C01107 | C12811 | -0.834103641 | 0.002698618 | inhibted |
| C05552 | C12811 | -0.68938906 | 0.027421814 | inhibted |
| C08842 | C12811 | 0.645406981 | 0.043863747 | actived |
| C14833 | C12811 | 0.727842955 | 0.017017922 | actived |
| C01290 | C12811 | -0.691940621 | 0.026623418 | inhibted |
| C00350 | C12811 | -0.737533718 | 0.014911229 | inhibted |
| C04230 | C12811 | 0.887860582 | 0.000603029 | actived |
| C02277 | C12811 | 0.835612322 | 0.002606799 | actived |
| C04692 | C12811 | -0.688477674 | 0.027710914 | inhibted |
| C07934 | C12811 | 0.734492536 | 0.015551561 | actived |
| C07468 | C12811 | 0.889698234 | 0.000565761 | actived |
| C08052 | C12811 | -0.666633612 | 0.035277603 | inhibted |
| C07363 | C12811 | 0.752195339 | 0.012080251 | actived |
| C07372 | C12811 | 0.783725796 | 0.007304638 | actived |
| C00141 | C07913 | -0.737018743 | 0.015018342 | inhibted |
| C00212 | C07913 | 0.705289162 | 0.022704752 | actived |
| C00058 | C07913 | 0.651296756 | 0.041354555 | actived |
| C00137 | C07913 | 0.654850013 | 0.039888438 | actived |
| C01879 | C07913 | 0.634571087 | 0.048742638 | actived |
| C19654 | C07913 | 0.831278385 | 0.002876796 | actived |
| C01595 | C07913 | 0.634099379 | 0.048962877 | actived |
| C01530 | C07913 | 0.888440643 | 0.000591077 | actived |
| C08278 | C07913 | -0.712110918 | 0.02086443 | inhibted |
| C04299 | C07913 | 0.814180208 | 0.004140658 | actived |
| C06524 | C07913 | 0.709603226 | 0.021528487 | actived |
| C01107 | C07913 | -0.658027948 | 0.038607185 | inhibted |
| C12621 | C07913 | -0.822195314 | 0.00350709 | inhibted |
| C19670 | C07913 | 0.829045866 | 0.003023462 | actived |
| C15589 | C07913 | -0.804772277 | 0.004984249 | inhibted |
| C05552 | C07913 | -0.673211437 | 0.032867774 | inhibted |
| C00521 | C07913 | -0.660072443 | 0.037797743 | inhibted |
| C06834 | C07913 | -0.803891332 | 0.005069052 | inhibted |
| C00350 | C07913 | -0.81967282 | 0.00369839 | inhibted |
| C04230 | C07913 | 0.639963725 | 0.046271613 | actived |
| C02277 | C07913 | 0.732738364 | 0.015929488 | actived |
| C04692 | C07913 | -0.746178139 | 0.013191827 | inhibted |
| C03577 | C07913 | -0.67567098 | 0.031996202 | inhibted |
| C07310 | C07913 | -0.704750306 | 0.022854716 | inhibted |
| C07934 | C07913 | 0.812137827 | 0.004314365 | actived |
| C08052 | C07913 | -0.790095575 | 0.006535278 | inhibted |
| C07363 | C07913 | 0.757641944 | 0.011132251 | actived |
| C07372 | C07913 | 0.741472197 | 0.014109598 | actived |
| C12811 | C07913 | 0.639266659 | 0.046586205 | actived |
| C00141 | C01948 | -0.758464589 | 0.010993762 | inhibted |
| C00212 | C01948 | 0.921168073 | 0.000153497 | actived |
| C00137 | C01948 | 0.775312924 | 0.008416404 | actived |
| C01879 | C01948 | 0.713590104 | 0.020479435 | actived |
| C19654 | C01948 | 0.739464641 | 0.014514349 | actived |
| C01595 | C01948 | 0.690478364 | 0.02707899 | actived |
| C01530 | C01948 | 0.704376835 | 0.022959053 | actived |
| C04299 | C01948 | 0.845101695 | 0.002080021 | actived |
| C06524 | C01948 | 0.703363957 | 0.02324367 | actived |
| C12621 | C01948 | -0.666349641 | 0.035384251 | inhibted |
| C19670 | C01948 | 0.745036115 | 0.013410593 | actived |
| C06425 | C01948 | -0.734214446 | 0.015611053 | inhibted |
| C05552 | C01948 | -0.69415428 | 0.025943773 | inhibted |
| C08842 | C01948 | 0.646514049 | 0.043384526 | actived |
| C00521 | C01948 | -0.711637804 | 0.020988617 | inhibted |
| C14833 | C01948 | 0.7471584 | 0.013006051 | actived |
| C06834 | C01948 | -0.632255304 | 0.049830231 | inhibted |
| C00350 | C01948 | -0.800139896 | 0.005441736 | inhibted |
| C04230 | C01948 | 0.674853805 | 0.032284015 | actived |
| C02277 | C01948 | 0.84604837 | 0.00203208 | actived |
| C04692 | C01948 | -0.716337034 | 0.019777549 | inhibted |
| C03577 | C01948 | -0.75038435 | 0.012407608 | inhibted |
| C07934 | C01948 | 0.897065346 | 0.000433056 | actived |
| C07468 | C01948 | 0.679981338 | 0.030506903 | actived |
| C08052 | C01948 | -0.635222816 | 0.048439436 | inhibted |
| C07363 | C01948 | 0.684038191 | 0.029148988 | actived |
| C07372 | C01948 | 0.637127983 | 0.047560316 | actived |
| C07913 | C01948 | 0.799871151 | 0.005469162 | actived |
| C00141 | C08497 | -0.632956266 | 0.049499341 | inhibted |
| C00212 | C08497 | 0.730428768 | 0.016436766 | actived |
| C00058 | C08497 | 0.851882527 | 0.001754068 | actived |
| C00137 | C08497 | 0.851639614 | 0.001765059 | actived |
| C01879 | C08497 | 0.917193482 | 0.000185958 | actived |
| C19654 | C08497 | 0.692269288 | 0.026521748 | actived |
| C01595 | C08497 | 0.885587907 | 0.000651564 | actived |
| C01530 | C08497 | 0.826052674 | 0.003228441 | actived |
| C08281 | C08497 | -0.706534229 | 0.022360842 | inhibted |
| C04299 | C08497 | 0.75485234 | 0.011610998 | actived |
| C06524 | C08497 | 0.77648455 | 0.008254847 | actived |
| C19670 | C08497 | 0.754570533 | 0.011660151 | actived |
| C06425 | C08497 | -0.653885928 | 0.040282716 | inhibted |
| C06866 | C08497 | 0.659907863 | 0.037862475 | actived |
| C05552 | C08497 | -0.824737511 | 0.003321588 | inhibted |
| C08842 | C08497 | 0.792007192 | 0.006316099 | actived |
| C00521 | C08497 | -0.704694263 | 0.022870352 | inhibted |
| C14833 | C08497 | 0.958625362 | 1.22E-05 | actived |
| C01290 | C08497 | -0.769799465 | 0.009206782 | inhibted |
| C00350 | C08497 | -0.758931636 | 0.010915678 | inhibted |
| C04230 | C08497 | 0.874836862 | 0.000920696 | actived |
| C02277 | C08497 | 0.904126299 | 0.000328787 | actived |
| C04692 | C08497 | -0.78477026 | 0.007174299 | inhibted |
| C07934 | C08497 | 0.939847496 | 5.32E-05 | actived |
| C07468 | C08497 | 0.871563413 | 0.001016663 | actived |
| C08052 | C08497 | -0.705334505 | 0.022692164 | inhibted |
| C07372 | C08497 | 0.685798063 | 0.028572968 | actived |
| C12811 | C08497 | 0.813626799 | 0.004187222 | actived |
| C07913 | C08497 | 0.755832742 | 0.011441128 | actived |
| C01948 | C08497 | 0.831791374 | 0.002843833 | actived |
| C00141 | C14467 | 0.868233717 | 0.001121547 | actived |
| C00164 | C14467 | -0.722473158 | 0.018270095 | inhibted |
| C17714 | C14467 | -0.754065979 | 0.011748519 | inhibted |
| C08278 | C14467 | 0.802536709 | 0.005201454 | actived |
| C08281 | C14467 | 0.842439284 | 0.002219226 | actived |
| C06524 | C14467 | -0.778473688 | 0.007985604 | inhibted |
| C00612 | C14467 | 0.788233778 | 0.006753886 | actived |
| C00184 | C14467 | -0.651767017 | 0.041158473 | inhibted |
| C05552 | C14467 | 0.807384564 | 0.004738738 | actived |
| C00521 | C14467 | 0.706177298 | 0.022459063 | actived |
| C06126 | C14467 | 0.654691782 | 0.03995297 | actived |
| C06834 | C14467 | 0.775383334 | 0.008406633 | actived |
| C00350 | C14467 | 0.788122448 | 0.006767121 | actived |
| C04598 | C14467 | -0.653967274 | 0.040249347 | inhibted |
| C04317 | C14467 | -0.723974466 | 0.017913782 | inhibted |
| C04692 | C14467 | 0.882153344 | 0.000730247 | actived |
| C07310 | C14467 | 0.806599543 | 0.004811587 | actived |
| C07598 | C14467 | -0.73327947 | 0.015812235 | inhibted |
| C08052 | C14467 | 0.670376961 | 0.033892038 | actived |
| C07372 | C14467 | -0.838868685 | 0.002416319 | inhibted |
| C00164 | C11882 | 0.720378114 | 0.018775492 | actived |
| C00058 | C11882 | 0.776176088 | 0.008297167 | actived |
| C00137 | C11882 | 0.843416348 | 0.002167382 | actived |
| C19654 | C11882 | 0.690444849 | 0.027089494 | actived |
| C17714 | C11882 | 0.859993844 | 0.001414722 | actived |
| C01530 | C11882 | 0.81696933 | 0.003911604 | actived |
| C08278 | C11882 | -0.684899144 | 0.028866211 | inhibted |
| C08281 | C11882 | -0.741305561 | 0.01414289 | inhibted |
| C06524 | C11882 | 0.826822484 | 0.003174797 | actived |
| C00612 | C11882 | -0.699237114 | 0.02442841 | inhibted |
| C01107 | C11882 | -0.63689514 | 0.047667183 | inhibted |
| C12621 | C11882 | -0.678891391 | 0.030878941 | inhibted |
| C00184 | C11882 | 0.699129614 | 0.024459813 | actived |
| C19670 | C11882 | 0.701530066 | 0.02376515 | actived |
| C06866 | C11882 | 0.641029207 | 0.045793501 | actived |
| C15589 | C11882 | -0.697413473 | 0.024964908 | inhibted |
| C05552 | C11882 | -0.891190119 | 0.000536768 | inhibted |
| C00521 | C11882 | -0.684221204 | 0.02908872 | inhibted |
| C14833 | C11882 | 0.724036938 | 0.017899061 | actived |
| C01290 | C11882 | -0.81010629 | 0.004492262 | inhibted |
| C06834 | C11882 | -0.682905732 | 0.02952381 | inhibted |
| C06575 | C11882 | -0.640773956 | 0.045907737 | inhibted |
| C05579 | C11882 | 0.860020827 | 0.00141368 | actived |
| C00157 | C11882 | 0.822591132 | 0.003477731 | actived |
| C00350 | C11882 | -0.77436633 | 0.008548554 | inhibted |
| C04230 | C11882 | 0.674585421 | 0.032378923 | actived |
| C04598 | C11882 | 0.755244652 | 0.011542813 | actived |
| C04317 | C11882 | 0.666266962 | 0.035415343 | actived |
| C02277 | C11882 | 0.718863603 | 0.01914682 | actived |
| C04692 | C11882 | -0.860131314 | 0.001409418 | inhibted |
| C03577 | C11882 | -0.645256196 | 0.043929291 | inhibted |
| C07310 | C11882 | -0.685071484 | 0.028809832 | inhibted |
| C07598 | C11882 | 0.920056314 | 0.000162118 | actived |
| C07468 | C11882 | 0.644326618 | 0.044334815 | actived |
| C08052 | C11882 | -0.957495401 | 1.36E-05 | inhibted |
| C07363 | C11882 | 0.674692551 | 0.032341016 | actived |
| C07372 | C11882 | 0.653570208 | 0.040412402 | actived |
| C07913 | C11882 | 0.691479046 | 0.026766653 | actived |
| C08497 | C11882 | 0.691274783 | 0.026830207 | actived |
| C14467 | C11882 | -0.702618686 | 0.023454634 | inhibted |
| C00058 | C10080 | 0.960934437 | 9.72E-06 | actived |
| C00137 | C10080 | 0.643850764 | 0.044543372 | actived |
| C01879 | C10080 | 0.734031829 | 0.015650206 | actived |
| C19654 | C10080 | 0.863471643 | 0.001284939 | actived |
| C01595 | C10080 | 0.935556847 | 6.98E-05 | actived |
| C01530 | C10080 | 0.869530646 | 0.001079802 | actived |
| C04299 | C10080 | 0.843957948 | 0.002139024 | actived |
| C06524 | C10080 | 0.866739996 | 0.001171067 | actived |
| C01107 | C10080 | -0.815739932 | 0.004011417 | inhibted |
| C12621 | C10080 | -0.684546604 | 0.028981774 | inhibted |
| C00184 | C10080 | 0.661830039 | 0.037111109 | actived |
| C06425 | C10080 | -0.661242004 | 0.037339889 | inhibted |
| C05552 | C10080 | -0.661345954 | 0.037299377 | inhibted |
| C14833 | C10080 | 0.682845645 | 0.029543789 | actived |
| C01290 | C10080 | -0.696944894 | 0.025104055 | inhibted |
| C00350 | C10080 | -0.710032 | 0.021413925 | inhibted |
| C04230 | C10080 | 0.813257296 | 0.00421852 | actived |
| C02277 | C10080 | 0.805266321 | 0.004937137 | actived |
| C03577 | C10080 | -0.640221973 | 0.046155425 | inhibted |
| C07934 | C10080 | 0.751233816 | 0.012253293 | actived |
| C07468 | C10080 | 0.828234553 | 0.003078069 | actived |
| C08052 | C10080 | -0.724445331 | 0.017803031 | inhibted |
| C07363 | C10080 | 0.838538232 | 0.002435175 | actived |
| C07372 | C10080 | 0.723430288 | 0.018042373 | actived |
| C12811 | C10080 | 0.952074359 | 2.18E-05 | actived |
| C07913 | C10080 | 0.670154528 | 0.03397332 | actived |
| C08497 | C10080 | 0.782482881 | 0.007461918 | actived |
| C11882 | C10080 | 0.670147492 | 0.033975893 | actived |
| C00212 | C10478 | 0.836349236 | 0.002562778 | actived |
| C00137 | C10478 | 0.832035211 | 0.00282826 | actived |
| C01530 | C10478 | 0.680543396 | 0.03031625 | actived |
| C19670 | C10478 | 0.817371138 | 0.003879372 | actived |
| C06866 | C10478 | 0.8657077 | 0.001206206 | actived |
| C05552 | C10478 | -0.663302509 | 0.03654239 | inhibted |
| C14833 | C10478 | 0.675509281 | 0.032053014 | actived |
| C01290 | C10478 | -0.672517975 | 0.033116394 | inhibted |
| C04692 | C10478 | -0.653964411 | 0.040250521 | inhibted |
| C07598 | C10478 | 0.632082965 | 0.049911808 | actived |
| C07934 | C10478 | 0.702829968 | 0.023394693 | actived |
| C07913 | C10478 | 0.717075155 | 0.019591822 | actived |
| C01948 | C10478 | 0.821397804 | 0.003566785 | actived |
| C08497 | C10478 | 0.713231595 | 0.020572291 | actived |
| C00141 | C07092 | 0.739165163 | 0.014575415 | actived |
| C00212 | C07092 | -0.808177423 | 0.004665965 | inhibted |
| C00164 | C07092 | -0.633858009 | 0.049075828 | inhibted |
| C00137 | C07092 | -0.72215427 | 0.018346406 | inhibted |
| C19654 | C07092 | -0.710259849 | 0.021353219 | inhibted |
| C17714 | C07092 | -0.711905839 | 0.020918198 | inhibted |
| C01595 | C07092 | -0.655294788 | 0.039707419 | inhibted |
| C04299 | C07092 | -0.725624842 | 0.017527686 | inhibted |
| C06524 | C07092 | -0.8476196 | 0.001954283 | inhibted |
| C01107 | C07092 | 0.643775467 | 0.044576432 | actived |
| C12621 | C07092 | 0.771175297 | 0.009004846 | actived |
| C00184 | C07092 | -0.915064184 | 0.000205293 | inhibted |
| C06425 | C07092 | 0.950345335 | 2.50E-05 | actived |
| C05552 | C07092 | 0.775643623 | 0.008370579 | actived |
| C00521 | C07092 | 0.688411449 | 0.027732002 | actived |
| C06126 | C07092 | 0.701663969 | 0.023726804 | actived |
| C06834 | C07092 | 0.655905088 | 0.039459935 | actived |
| C06575 | C07092 | 0.803137517 | 0.005142429 | actived |
| C00157 | C07092 | -0.664126511 | 0.036226717 | inhibted |
| C00350 | C07092 | 0.868544674 | 0.001111433 | actived |
| C04230 | C07092 | -0.648915717 | 0.042357001 | inhibted |
| C02277 | C07092 | -0.836260031 | 0.002568078 | inhibted |
| C04692 | C07092 | 0.743113346 | 0.013784629 | actived |
| C03577 | C07092 | 0.944716196 | 3.82E-05 | actived |
| C07598 | C07092 | -0.698758893 | 0.024568324 | inhibted |
| C07468 | C07092 | -0.650057712 | 0.041874195 | inhibted |
| C08052 | C07092 | 0.736640953 | 0.01509726 | actived |
| C07363 | C07092 | -0.805100371 | 0.004952926 | inhibted |
| C01948 | C07092 | -0.776431467 | 0.008262119 | inhibted |
| C14467 | C07092 | 0.67553141 | 0.032045235 | actived |
| C11882 | C07092 | -0.700348125 | 0.024105472 | inhibted |
| C00141 | C13482 | 0.682414429 | 0.029687439 | actived |
| C00164 | C13482 | -0.663776383 | 0.036360624 | inhibted |
| C00058 | C13482 | -0.880380663 | 0.000773465 | inhibted |
| C00137 | C13482 | -0.646042665 | 0.043588144 | inhibted |
| C01879 | C13482 | -0.792577808 | 0.006251701 | inhibted |
| C19654 | C13482 | -0.825036459 | 0.003300248 | inhibted |
| C01595 | C13482 | -0.941954017 | 4.63E-05 | inhibted |
| C01530 | C13482 | -0.825398589 | 0.003274529 | inhibted |
| C04299 | C13482 | -0.893482732 | 0.000494353 | inhibted |
| C06524 | C13482 | -0.896300475 | 0.000445647 | inhibted |
| C01107 | C13482 | 0.805702044 | 0.004895851 | actived |
| C12621 | C13482 | 0.661743841 | 0.037144585 | actived |
| C19670 | C13482 | -0.740643612 | 0.014275684 | inhibted |
| C06425 | C13482 | 0.729025729 | 0.016750354 | actived |
| C05552 | C13482 | 0.724317538 | 0.017833042 | actived |
| C08842 | C13482 | -0.678756914 | 0.030925056 | inhibted |
| C14833 | C13482 | -0.741340724 | 0.014135861 | inhibted |
| C01290 | C13482 | 0.665719338 | 0.035621746 | actived |
| C00350 | C13482 | 0.83611095 | 0.002576954 | actived |
| C04230 | C13482 | -0.865988101 | 0.001196586 | inhibted |
| C02277 | C13482 | -0.899697571 | 0.000391713 | inhibted |
| C04692 | C13482 | 0.753610772 | 0.011828648 | actived |
| C03577 | C13482 | 0.675422208 | 0.032083635 | actived |
| C07934 | C13482 | -0.822570299 | 0.003479272 | inhibted |
| C07468 | C13482 | -0.860755011 | 0.001385539 | inhibted |
| C08052 | C13482 | 0.705698571 | 0.022591267 | actived |
| C07363 | C13482 | -0.826759499 | 0.003179162 | inhibted |
| C07372 | C13482 | -0.828816615 | 0.003038821 | inhibted |
| C12811 | C13482 | -0.943543874 | 4.15E-05 | inhibted |
| C07913 | C13482 | -0.774319972 | 0.008555063 | inhibted |
| C01948 | C13482 | -0.789352035 | 0.00662197 | inhibted |
| C08497 | C13482 | -0.853687174 | 0.001673958 | inhibted |
| C10080 | C13482 | -0.900691258 | 0.000376886 | inhibted |
| C07092 | C13482 | 0.692565967 | 0.026430202 | actived |
| C00141 | C06205 | -0.772721222 | 0.008781698 | inhibted |
| C00212 | C06205 | 0.883920031 | 0.000688956 | actived |
| C00058 | C06205 | 0.690719086 | 0.027003628 | actived |
| C00137 | C06205 | 0.786464083 | 0.006966446 | actived |
| C01879 | C06205 | 0.794088662 | 0.006083445 | actived |
| C19654 | C06205 | 0.679781425 | 0.03057491 | actived |
| C01595 | C06205 | 0.810475494 | 0.004459548 | actived |
| C01530 | C06205 | 0.659027896 | 0.038209847 | actived |
| C08281 | C06205 | -0.667141537 | 0.035087391 | inhibted |
| C04299 | C06205 | 0.787819021 | 0.006803284 | actived |
| C06524 | C06205 | 0.806178933 | 0.004850947 | actived |
| C12621 | C06205 | -0.657184267 | 0.038944588 | inhibted |
| C00184 | C06205 | 0.720000681 | 0.01886756 | actived |
| C06425 | C06205 | -0.894887484 | 0.000469609 | inhibted |
| C05552 | C06205 | -0.794583715 | 0.006029022 | inhibted |
| C00521 | C06205 | -0.77160545 | 0.008942358 | inhibted |
| C14833 | C06205 | 0.801139253 | 0.005340608 | actived |
| C06834 | C06205 | -0.671198853 | 0.033592843 | inhibted |
| C06575 | C06205 | -0.751130397 | 0.012272008 | inhibted |
| C00350 | C06205 | -0.864186143 | 0.001259387 | inhibted |
| C04230 | C06205 | 0.806125787 | 0.004855937 | actived |
| C02277 | C06205 | 0.94830081 | 2.94E-05 | actived |
| C04692 | C06205 | -0.764707383 | 0.009981997 | inhibted |
| C03577 | C06205 | -0.804017446 | 0.005056849 | inhibted |
| C07934 | C06205 | 0.858369687 | 0.001478479 | actived |
| C07468 | C06205 | 0.818980493 | 0.003752177 | actived |
| C08052 | C06205 | -0.645269101 | 0.043923679 | inhibted |
| C07363 | C06205 | 0.697166397 | 0.025038212 | actived |
| C07372 | C06205 | 0.637925833 | 0.047195342 | actived |
| C12811 | C06205 | 0.677089952 | 0.031500587 | actived |
| C07913 | C06205 | 0.692986457 | 0.026300822 | actived |
| C01948 | C06205 | 0.928692153 | 0.000103722 | actived |
| C08497 | C06205 | 0.860077757 | 0.001411483 | actived |
| C10080 | C06205 | 0.671897609 | 0.033339886 | actived |
| C10478 | C06205 | 0.664274983 | 0.036170034 | actived |
| C07092 | C06205 | -0.877365256 | 0.000851226 | inhibted |
| C13482 | C06205 | -0.814111703 | 0.004146401 | inhibted |
| C00141 | C14487 | 0.676469972 | 0.031716485 | actived |
| C00058 | C14487 | -0.78510287 | 0.007133141 | inhibted |
| C00137 | C14487 | -0.682053166 | 0.029808152 | inhibted |
| C01879 | C14487 | -0.656642153 | 0.039162434 | inhibted |
| C19654 | C14487 | -0.714980512 | 0.020122048 | inhibted |
| C17714 | C14487 | -0.837731636 | 0.002481648 | inhibted |
| C01595 | C14487 | -0.701077224 | 0.023895146 | inhibted |
| C01530 | C14487 | -0.85538925 | 0.001600862 | inhibted |
| C08278 | C14487 | 0.639172787 | 0.046628679 | actived |
| C08281 | C14487 | 0.666666841 | 0.035265138 | actived |
| C04299 | C14487 | -0.651555352 | 0.041246652 | inhibted |
| C06524 | C14487 | -0.850532376 | 0.00181579 | inhibted |
| C00612 | C14487 | 0.650521649 | 0.041679115 | actived |
| C01107 | C14487 | 0.758291582 | 0.011022786 | actived |
| C12621 | C14487 | 0.815183423 | 0.004057193 | actived |
| C00184 | C14487 | -0.706189332 | 0.022455747 | inhibted |
| C19670 | C14487 | -0.717221724 | 0.019555086 | inhibted |
| C06425 | C14487 | 0.694743166 | 0.025764993 | actived |
| C15589 | C14487 | 0.82328687 | 0.003426553 | actived |
| C05552 | C14487 | 0.833620927 | 0.002728482 | actived |
| C14833 | C14487 | -0.676471716 | 0.031715876 | inhibted |
| C06126 | C14487 | 0.787863127 | 0.006798019 | actived |
| C01290 | C14487 | 0.670886472 | 0.033706347 | actived |
| C06834 | C14487 | 0.752788845 | 0.011974299 | actived |
| C06575 | C14487 | 0.67829835 | 0.031082659 | actived |
| C05579 | C14487 | -0.754584037 | 0.011657792 | inhibted |
| C00350 | C14487 | 0.851015702 | 0.001793517 | actived |
| C04230 | C14487 | -0.772160799 | 0.008862136 | inhibted |
| C04598 | C14487 | -0.716475309 | 0.019742663 | inhibted |
| C02277 | C14487 | -0.780031235 | 0.007779164 | inhibted |
| C04692 | C14487 | 0.846461315 | 0.002011421 | actived |
| C03577 | C14487 | 0.692704595 | 0.026387499 | actived |
| C07598 | C14487 | -0.788545306 | 0.006716951 | inhibted |
| C07468 | C14487 | -0.713970302 | 0.020381278 | inhibted |
| C08052 | C14487 | 0.929084277 | 0.000101508 | actived |
| C07363 | C14487 | -0.726344238 | 0.017361209 | inhibted |
| C07372 | C14487 | -0.706010947 | 0.022504942 | inhibted |
| C12811 | C14487 | -0.725243566 | 0.017616365 | inhibted |
| C07913 | C14487 | -0.825385422 | 0.003275461 | inhibted |
| C08497 | C14487 | -0.709619306 | 0.021524183 | inhibted |
| C14467 | C14487 | 0.743927234 | 0.013625425 | actived |
| C11882 | C14487 | -0.863352449 | 0.001289238 | inhibted |
| C10080 | C14487 | -0.735896515 | 0.015253616 | inhibted |
| C07092 | C14487 | 0.691298367 | 0.026822864 | actived |
| C13482 | C14487 | 0.748700303 | 0.012717551 | actived |
| C00141 | C11129 | 0.918762293 | 0.000172596 | actived |
| C00212 | C11129 | -0.709039864 | 0.02167965 | inhibted |
| C08278 | C11129 | 0.884175985 | 0.000683118 | actived |
| C08281 | C11129 | 0.780756597 | 0.007684326 | actived |
| C06524 | C11129 | -0.680905349 | 0.030193905 | inhibted |
| C00612 | C11129 | 0.66619049 | 0.035444117 | actived |
| C01107 | C11129 | 0.65385853 | 0.040293959 | actived |
| C06425 | C11129 | 0.700180677 | 0.024153955 | actived |
| C05552 | C11129 | 0.765403429 | 0.009873415 | actived |
| C00521 | C11129 | 0.877404292 | 0.000850184 | actived |
| C06834 | C11129 | 0.95331275 | 1.96E-05 | actived |
| C06575 | C11129 | 0.713340542 | 0.020544043 | actived |
| C00350 | C11129 | 0.892146327 | 0.000518766 | actived |
| C04230 | C11129 | -0.674041979 | 0.03257168 | inhibted |
| C02277 | C11129 | -0.756698704 | 0.011292546 | inhibted |
| C04692 | C11129 | 0.837457018 | 0.002497615 | actived |
| C07310 | C11129 | 0.853660036 | 0.001675142 | actived |
| C07468 | C11129 | -0.639625317 | 0.046424162 | inhibted |
| C07372 | C11129 | -0.701732651 | 0.023707152 | inhibted |
| C07913 | C11129 | -0.74648537 | 0.013133403 | inhibted |
| C01948 | C11129 | -0.66741878 | 0.03498386 | inhibted |
| C08497 | C11129 | -0.640821383 | 0.045886497 | inhibted |
| C14467 | C11129 | 0.755288209 | 0.01153526 | actived |
| C07092 | C11129 | 0.685660428 | 0.028617734 | actived |
| C06205 | C11129 | -0.737898842 | 0.014835609 | inhibted |
| C14487 | C11129 | 0.735678623 | 0.015299592 | actived |
| C00141 | C00137 | -0.757575758 | 0.011143447 | inhibted |
| C00141 | C01879 | -0.637811507 | 0.047247525 | inhibted |
| C00141 | C19654 | -0.745454545 | 0.013330146 | inhibted |
| C00212 | C19654 | 0.696969697 | 0.025096676 | actived |
| C00141 | C01595 | -0.684848485 | 0.028882798 | inhibted |
| C17714 | C01595 | 0.684848485 | 0.028882798 | actived |
| C00141 | C01530 | -0.672727273 | 0.033041223 | inhibted |
| C00137 | C08278 | -0.793939394 | 0.006099923 | inhibted |
| C19654 | C08278 | -0.757575758 | 0.011143447 | inhibted |
| C01530 | C08278 | -0.757575758 | 0.011143447 | inhibted |
| C19654 | C08281 | -0.705574587 | 0.022625593 | inhibted |
| C01595 | C08281 | -0.644220275 | 0.044381366 | inhibted |
| C01530 | C08281 | -0.791470624 | 0.006377084 | inhibted |
| C00141 | C04299 | -0.733333333 | 0.015800596 | inhibted |
| C00058 | C00612 | -0.721212121 | 0.018573155 | inhibted |
| C00137 | C00612 | -0.648484848 | 0.042540128 | inhibted |
| C01595 | C00612 | -0.660606061 | 0.037588378 | inhibted |
| C08278 | C00612 | 0.745454545 | 0.013330146 | actived |
| C00141 | C12621 | 0.790277207 | 0.006514224 | actived |
| C00058 | C12621 | -0.656537987 | 0.039204386 | inhibted |
| C08278 | C12621 | 0.753802874 | 0.011794786 | actived |
| C08281 | C12621 | 0.683106026 | 0.029457279 | actived |
| C01107 | C12621 | 0.668696098 | 0.034509542 | actived |
| C00141 | C00184 | -0.733333333 | 0.015800596 | inhibted |
| C00164 | C00184 | 0.721212121 | 0.018573155 | actived |
| C01595 | C00184 | 0.672727273 | 0.033041223 | actived |
| C08281 | C00184 | -0.668762 | 0.034485189 | inhibted |
| C00612 | C00184 | -0.672727273 | 0.033041223 | inhibted |
| C01107 | C00184 | -0.709090909 | 0.021665923 | inhibted |
| C00141 | C19670 | -0.709090909 | 0.021665923 | inhibted |
| C08278 | C19670 | -0.76969697 | 0.009221953 | inhibted |
| C08281 | C19670 | -0.693303725 | 0.02620349 | inhibted |
| C00141 | C15589 | 0.721212121 | 0.018573155 | actived |
| C00212 | C15589 | -0.636363636 | 0.047911726 | inhibted |
| C08278 | C15589 | 0.745454545 | 0.013330146 | actived |
| C08281 | C15589 | 0.668762 | 0.034485189 | actived |
| C19654 | C05552 | -0.672727273 | 0.033041223 | inhibted |
| C12621 | C05552 | 0.753802874 | 0.011794786 | actived |
| C15589 | C05552 | 0.721212121 | 0.018573155 | actived |
| C00137 | C08842 | 0.696969697 | 0.025096676 | actived |
| C00058 | C00521 | -0.648484848 | 0.042540128 | inhibted |
| C19654 | C00521 | -0.709090909 | 0.021665923 | inhibted |
| C01595 | C00521 | -0.648484848 | 0.042540128 | inhibted |
| C01530 | C00521 | -0.696969697 | 0.025096676 | inhibted |
| C12621 | C00521 | 0.632221765 | 0.0498461 | actived |
| C00184 | C00521 | -0.648484848 | 0.042540128 | inhibted |
| C00141 | C14833 | -0.660606061 | 0.037588378 | inhibted |
| C17714 | C14833 | 0.733333333 | 0.015800596 | actived |
| C08278 | C14833 | -0.696969697 | 0.025096676 | inhibted |
| C15589 | C14833 | -0.636363636 | 0.047911726 | inhibted |
| C00141 | C01290 | 0.684848485 | 0.028882798 | actived |
| C19654 | C01290 | -0.684848485 | 0.028882798 | inhibted |
| C08278 | C01290 | 0.757575758 | 0.011143447 | actived |
| C12621 | C01290 | 0.644379876 | 0.044311514 | actived |
| C15589 | C01290 | 0.672727273 | 0.033041223 | actived |
| C00521 | C01290 | 0.721212121 | 0.018573155 | actived |
| C00058 | C06834 | -0.709090909 | 0.021665923 | inhibted |
| C00137 | C06834 | -0.733333333 | 0.015800596 | inhibted |
| C19654 | C06834 | -0.866666667 | 0.001173538 | inhibted |
| C17714 | C06834 | -0.696969697 | 0.025096676 | inhibted |
| C01595 | C06834 | -0.636363636 | 0.047911726 | inhibted |
| C04299 | C06834 | -0.793939394 | 0.006099923 | inhibted |
| C00612 | C06834 | 0.684848485 | 0.028882798 | actived |
| C12621 | C06834 | 0.948332648 | 2.93E-05 | actived |
| C00184 | C06834 | -0.721212121 | 0.018573155 | inhibted |
| C19670 | C06834 | -0.733333333 | 0.015800596 | inhibted |
| C06425 | C06834 | 0.696969697 | 0.025096676 | actived |
| C15589 | C06834 | 0.903030303 | 0.000343612 | actived |
| C14833 | C06834 | -0.684848485 | 0.028882798 | inhibted |
| C01290 | C06834 | 0.672727273 | 0.033041223 | actived |
| C00058 | C06575 | -0.660606061 | 0.037588378 | inhibted |
| C00164 | C05579 | 0.672727273 | 0.033041223 | actived |
| C00058 | C05579 | 0.660606061 | 0.037588378 | actived |
| C01107 | C05579 | -0.733333333 | 0.015800596 | inhibted |
| C00141 | C00157 | -0.636363636 | 0.047911726 | inhibted |
| C00212 | C00157 | 0.696969697 | 0.025096676 | actived |
| C01595 | C00157 | 0.636363636 | 0.047911726 | actived |
| C08278 | C00157 | -0.696969697 | 0.025096676 | inhibted |
| C00612 | C00157 | -0.660606061 | 0.037588378 | inhibted |
| C06575 | C00157 | -0.648484848 | 0.042540128 | inhibted |
| C15589 | C00350 | 0.806060606 | 0.004862061 | actived |
| C00141 | C04230 | -0.719101209 | 0.01908823 | inhibted |
| C17714 | C04230 | 0.694088993 | 0.025963645 | actived |
| C08278 | C04230 | -0.650317615 | 0.041764833 | inhibted |
| C08281 | C04230 | -0.639355767 | 0.04654591 | inhibted |
| C12621 | C04230 | -0.6397531 | 0.04636652 | inhibted |
| C00184 | C04230 | 0.706595101 | 0.022344121 | actived |
| C00521 | C04230 | -0.694088993 | 0.025963645 | inhibted |
| C06834 | C04230 | -0.725354263 | 0.017590587 | inhibted |
| C00157 | C04230 | 0.731607317 | 0.016176528 | actived |
| C00058 | C04598 | 0.709090909 | 0.021665923 | actived |
| C00212 | C04317 | 0.709090909 | 0.021665923 | actived |
| C00137 | C04317 | 0.721212121 | 0.018573155 | actived |
| C17714 | C04317 | 0.660606061 | 0.037588378 | actived |
| C06524 | C04317 | 0.684848485 | 0.028882798 | actived |
| C19670 | C04317 | 0.684848485 | 0.028882798 | actived |
| C00521 | C04317 | -0.745454545 | 0.013330146 | inhibted |
| C14833 | C04317 | 0.684848485 | 0.028882798 | actived |
| C06126 | C04317 | -0.636363636 | 0.047911726 | inhibted |
| C01290 | C04317 | -0.696969697 | 0.025096676 | inhibted |
| C06834 | C04317 | -0.648484848 | 0.042540128 | inhibted |
| C00157 | C04317 | 0.696969697 | 0.025096676 | actived |
| C00350 | C04317 | -0.684848485 | 0.028882798 | inhibted |
| C17714 | C02277 | 0.636363636 | 0.047911726 | actived |
| C08278 | C02277 | -0.660606061 | 0.037588378 | inhibted |
| C15589 | C02277 | -0.636363636 | 0.047911726 | inhibted |
| C00157 | C02277 | 0.648484848 | 0.042540128 | actived |
| C19654 | C04692 | -0.660606061 | 0.037588378 | inhibted |
| C04299 | C04692 | -0.636363636 | 0.047911726 | inhibted |
| C12621 | C04692 | 0.765960985 | 0.00978704 | actived |
| C15589 | C04692 | 0.709090909 | 0.021665923 | actived |
| C00141 | C03577 | 0.793939394 | 0.006099923 | actived |
| C05552 | C03577 | 0.660606061 | 0.037588378 | actived |
| C06834 | C03577 | 0.757575758 | 0.011143447 | actived |
| C04230 | C03577 | -0.694088993 | 0.025963645 | inhibted |
| C04692 | C03577 | 0.672727273 | 0.033041223 | actived |
| C00212 | C07310 | -0.745454545 | 0.013330146 | inhibted |
| C00137 | C07310 | -0.709090909 | 0.021665923 | inhibted |
| C19654 | C07310 | -0.709090909 | 0.021665923 | inhibted |
| C01530 | C07310 | -0.684848485 | 0.028882798 | inhibted |
| C00612 | C07310 | 0.709090909 | 0.021665923 | actived |
| C12621 | C07310 | 0.674775153 | 0.032311809 | actived |
| C19670 | C07310 | -0.721212121 | 0.018573155 | inhibted |
| C15589 | C07310 | 0.672727273 | 0.033041223 | actived |
| C01290 | C07310 | 0.733333333 | 0.015800596 | actived |
| C00157 | C07310 | -0.709090909 | 0.021665923 | inhibted |
| C00141 | C07598 | -0.660606061 | 0.037588378 | inhibted |
| C00164 | C07598 | 0.660606061 | 0.037588378 | actived |
| C01595 | C07598 | 0.660606061 | 0.037588378 | actived |
| C08278 | C07598 | -0.672727273 | 0.033041223 | inhibted |
| C19670 | C07598 | 0.636363636 | 0.047911726 | actived |
| C15589 | C07598 | -0.636363636 | 0.047911726 | inhibted |
| C00521 | C07598 | -0.672727273 | 0.033041223 | inhibted |
| C06834 | C07598 | -0.660606061 | 0.037588378 | inhibted |
| C04230 | C07598 | 0.644064561 | 0.044449588 | actived |
| C07310 | C07598 | -0.660606061 | 0.037588378 | inhibted |
| C00141 | C07934 | -0.793939394 | 0.006099923 | inhibted |
| C17714 | C07934 | 0.660606061 | 0.037588378 | actived |
| C08278 | C07934 | -0.781818182 | 0.007547008 | inhibted |
| C08281 | C07934 | -0.730116312 | 0.016506245 | inhibted |
| C01107 | C07934 | -0.648484848 | 0.042540128 | inhibted |
| C00184 | C07934 | 0.648484848 | 0.042540128 | actived |
| C15589 | C07934 | -0.818181818 | 0.00381492 | inhibted |
| C01290 | C07934 | -0.709090909 | 0.021665923 | inhibted |
| C06834 | C07934 | -0.890909091 | 0.000542144 | inhibted |
| C06575 | C07934 | -0.660606061 | 0.037588378 | inhibted |
| C03577 | C07934 | -0.745454545 | 0.013330146 | inhibted |
| C07310 | C07934 | -0.648484848 | 0.042540128 | inhibted |
| C00141 | C07468 | -0.731607317 | 0.016176528 | inhibted |
| C00212 | C07468 | 0.681582885 | 0.029965793 | actived |
| C17714 | C07468 | 0.644064561 | 0.044449588 | actived |
| C08278 | C07468 | -0.687835939 | 0.027915724 | inhibted |
| C08281 | C07468 | -0.664676787 | 0.036016937 | inhibted |
| C00612 | C07468 | -0.694088993 | 0.025963645 | inhibted |
| C12621 | C07468 | -0.6397531 | 0.04636652 | inhibted |
| C00184 | C07468 | 0.644064561 | 0.044449588 | actived |
| C00521 | C07468 | -0.756619533 | 0.011306073 | inhibted |
| C06834 | C07468 | -0.737860371 | 0.014843564 | inhibted |
| C00157 | C07468 | 0.719101209 | 0.01908823 | actived |
| C03577 | C07468 | -0.669076777 | 0.034369028 | inhibted |
| C08278 | C08052 | 0.721212121 | 0.018573155 | actived |
| C08281 | C08052 | 0.681032862 | 0.030150884 | actived |
| C00521 | C08052 | 0.636363636 | 0.047911726 | actived |
| C07310 | C08052 | 0.696969697 | 0.025096676 | actived |
| C00141 | C07363 | -0.733333333 | 0.015800596 | inhibted |
| C08278 | C07363 | -0.660606061 | 0.037588378 | inhibted |
| C07310 | C07363 | -0.660606061 | 0.037588378 | inhibted |
| C00137 | C07372 | 0.672727273 | 0.033041223 | actived |
| C00612 | C07372 | -0.76969697 | 0.009221953 | inhibted |
| C12621 | C07372 | -0.656537987 | 0.039204386 | inhibted |
| C14833 | C07372 | 0.660606061 | 0.037588378 | actived |
| C04230 | C07372 | 0.637811507 | 0.047247525 | actived |
| C04317 | C07372 | 0.696969697 | 0.025096676 | actived |
| C07598 | C07372 | 0.709090909 | 0.021665923 | actived |
| C07934 | C07372 | 0.636363636 | 0.047911726 | actived |
| C17714 | C12811 | 0.652973266 | 0.040658371 | actived |
| C08281 | C12811 | -0.634855367 | 0.048610228 | inhibted |
| C00612 | C12811 | -0.640043102 | 0.04623588 | inhibted |
| C06834 | C12811 | -0.665903429 | 0.03555227 | inhibted |
| C08281 | C07913 | -0.705574587 | 0.022625593 | inhibted |
| C07468 | C07913 | 0.637811507 | 0.047247525 | actived |
| C08278 | C01948 | -0.757575758 | 0.011143447 | inhibted |
| C15589 | C01948 | -0.660606061 | 0.037588378 | inhibted |
| C07310 | C01948 | -0.660606061 | 0.037588378 | inhibted |
| C17714 | C08497 | 0.757575758 | 0.011143447 | actived |
| C08278 | C08497 | -0.721212121 | 0.018573155 | inhibted |
| C00612 | C08497 | -0.721212121 | 0.018573155 | inhibted |
| C12621 | C08497 | -0.668696098 | 0.034509542 | inhibted |
| C15589 | C08497 | -0.660606061 | 0.037588378 | inhibted |
| C06834 | C08497 | -0.757575758 | 0.011143447 | inhibted |
| C06575 | C08497 | -0.636363636 | 0.047911726 | inhibted |
| C07598 | C08497 | 0.76969697 | 0.009221953 | actived |
| C12621 | C14467 | 0.674775153 | 0.032311809 | actived |
| C19670 | C14467 | -0.672727273 | 0.033041223 | inhibted |
| C15589 | C14467 | 0.648484848 | 0.042540128 | actived |
| C01290 | C14467 | 0.696969697 | 0.025096676 | actived |
| C00157 | C14467 | -0.648484848 | 0.042540128 | inhibted |
| C07363 | C14467 | -0.648484848 | 0.042540128 | inhibted |
| C07913 | C14467 | -0.660606061 | 0.037588378 | inhibted |
| C07934 | C11882 | 0.660606061 | 0.037588378 | actived |
| C00141 | C10080 | -0.696969697 | 0.025096676 | inhibted |
| C00164 | C10080 | 0.721212121 | 0.018573155 | actived |
| C17714 | C10080 | 0.757575758 | 0.011143447 | actived |
| C08278 | C10080 | -0.672727273 | 0.033041223 | inhibted |
| C08281 | C10080 | -0.668762 | 0.034485189 | inhibted |
| C00612 | C10080 | -0.636363636 | 0.047911726 | inhibted |
| C19670 | C10080 | 0.709090909 | 0.021665923 | actived |
| C15589 | C10080 | -0.793939394 | 0.006099923 | inhibted |
| C06834 | C10080 | -0.854545455 | 0.001636803 | inhibted |
| C05579 | C10080 | 0.793939394 | 0.006099923 | actived |
| C04692 | C10080 | -0.733333333 | 0.015800596 | inhibted |
| C07598 | C10080 | 0.696969697 | 0.025096676 | actived |
| C01948 | C10080 | 0.745454545 | 0.013330146 | actived |
| C00141 | C10478 | -0.757575758 | 0.011143447 | inhibted |
| C00058 | C10478 | 0.648484848 | 0.042540128 | actived |
| C19654 | C10478 | 0.721212121 | 0.018573155 | actived |
| C17714 | C10478 | 0.672727273 | 0.033041223 | actived |
| C08278 | C10478 | -0.793939394 | 0.006099923 | inhibted |
| C08281 | C10478 | -0.754658037 | 0.011644873 | inhibted |
| C06524 | C10478 | 0.660606061 | 0.037588378 | actived |
| C12621 | C10478 | -0.705170431 | 0.022737737 | inhibted |
| C15589 | C10478 | -0.757575758 | 0.011143447 | inhibted |
| C00521 | C10478 | -0.745454545 | 0.013330146 | inhibted |
| C06834 | C10478 | -0.684848485 | 0.028882798 | inhibted |
| C00157 | C10478 | 0.660606061 | 0.037588378 | actived |
| C00350 | C10478 | -0.648484848 | 0.042540128 | inhibted |
| C04317 | C10478 | 0.793939394 | 0.006099923 | actived |
| C07310 | C10478 | -0.757575758 | 0.011143447 | inhibted |
| C08052 | C10478 | -0.696969697 | 0.025096676 | inhibted |
| C07372 | C10478 | 0.660606061 | 0.037588378 | actived |
| C14467 | C10478 | -0.696969697 | 0.025096676 | inhibted |
| C11882 | C10478 | 0.721212121 | 0.018573155 | actived |
| C15589 | C07092 | 0.709090909 | 0.021665923 | actived |
| C07934 | C07092 | -0.709090909 | 0.021665923 | inhibted |
| C07913 | C07092 | -0.636363636 | 0.047911726 | inhibted |
| C10080 | C07092 | -0.672727273 | 0.033041223 | inhibted |
| C00212 | C13482 | -0.660606061 | 0.037588378 | inhibted |
| C08278 | C13482 | 0.757575758 | 0.011143447 | actived |
| C08281 | C13482 | 0.668762 | 0.034485189 | actived |
| C00184 | C13482 | -0.721212121 | 0.018573155 | inhibted |
| C15589 | C13482 | 0.709090909 | 0.021665923 | actived |
| C00521 | C13482 | 0.672727273 | 0.033041223 | actived |
| C06834 | C13482 | 0.854545455 | 0.001636803 | actived |
| C06575 | C13482 | 0.636363636 | 0.047911726 | actived |
| C00157 | C13482 | -0.648484848 | 0.042540128 | inhibted |
| C07310 | C13482 | 0.672727273 | 0.033041223 | actived |
| C14467 | C13482 | 0.684848485 | 0.028882798 | actived |
| C08278 | C06205 | -0.660606061 | 0.037588378 | inhibted |
| C01290 | C06205 | -0.636363636 | 0.047911726 | inhibted |
| C07934 | C14487 | -0.745454545 | 0.013330146 | inhibted |
| C00058 | C11129 | -0.696969697 | 0.025096676 | inhibted |
| C00137 | C11129 | -0.721212121 | 0.018573155 | inhibted |
| C01879 | C11129 | -0.650317615 | 0.041764833 | inhibted |
| C19654 | C11129 | -0.709090909 | 0.021665923 | inhibted |
| C17714 | C11129 | -0.733333333 | 0.015800596 | inhibted |
| C01595 | C11129 | -0.672727273 | 0.033041223 | inhibted |
| C01530 | C11129 | -0.721212121 | 0.018573155 | inhibted |
| C04299 | C11129 | -0.684848485 | 0.028882798 | inhibted |
| C12621 | C11129 | 0.851067761 | 0.00179113 | actived |
| C00184 | C11129 | -0.721212121 | 0.018573155 | inhibted |
| C15589 | C11129 | 0.818181818 | 0.00381492 | actived |
| C14833 | C11129 | -0.721212121 | 0.018573155 | inhibted |
| C06126 | C11129 | 0.709090909 | 0.021665923 | actived |
| C04317 | C11129 | -0.733333333 | 0.015800596 | inhibted |
| C03577 | C11129 | 0.76969697 | 0.009221953 | actived |
| C07598 | C11129 | -0.660606061 | 0.037588378 | inhibted |
| C07934 | C11129 | -0.878787879 | 0.000813862 | inhibted |
| C08052 | C11129 | 0.660606061 | 0.037588378 | actived |
| C10080 | C11129 | -0.793939394 | 0.006099923 | inhibted |
| C10478 | C11129 | -0.648484848 | 0.042540128 | inhibted |
| C13482 | C11129 | 0.806060606 | 0.004862061 | actived |
|  |  |  |  |  |
| C00109 | C00141 | 0.884550578 | 0.00067464 | actived |
| C00109 | C00149 | -0.739112821 | 0.014586106 | inhibted |
| C00082 | C00081 | -0.651716115 | 0.041179668 | inhibted |
| C00149 | C02571 | -0.696894204 | 0.025119139 | inhibted |
| C00109 | C00299 | -0.783178721 | 0.007373573 | inhibted |
| C00141 | C00299 | -0.819772427 | 0.003690698 | inhibted |
| C05476 | C00785 | 0.691005855 | 0.026914038 | actived |
| C00149 | C01571 | -0.68303993 | 0.029479223 | inhibted |
| C00081 | C01571 | 0.637398743 | 0.047436247 | actived |
| C02571 | C01571 | 0.824114713 | 0.003366365 | actived |
| C00141 | C05607 | 0.708436429 | 0.021842376 | actived |
| C05476 | C05607 | 0.890658694 | 0.000546968 | actived |
| C00109 | C08262 | 0.648642591 | 0.042473023 | actived |
| C00149 | C08262 | -0.733037227 | 0.015864652 | inhibted |
| C00082 | C08262 | -0.64099597 | 0.045808365 | inhibted |
| C00081 | C08262 | 0.685456662 | 0.028684098 | actived |
| C05476 | C08262 | 0.695370941 | 0.02557534 | actived |
| C00299 | C08262 | -0.690097943 | 0.02719838 | inhibted |
| C00785 | C08262 | 0.749433529 | 0.012581944 | actived |
| C05607 | C08262 | 0.744317945 | 0.013549457 | actived |
| C00109 | C00182 | -0.649825636 | 0.04197201 | inhibted |
| C02571 | C00182 | -0.703675804 | 0.023155784 | inhibted |
| C01571 | C00182 | -0.636582556 | 0.047810901 | inhibted |
| C00082 | C01530 | -0.709739876 | 0.02149193 | inhibted |
| C05476 | C01530 | 0.835253793 | 0.002628412 | actived |
| C05607 | C01530 | 0.833260219 | 0.002750952 | actived |
| C08262 | C01530 | 0.8072693 | 0.004749385 | actived |
| C00149 | C08278 | -0.700767859 | 0.023984235 | inhibted |
| C02571 | C08278 | 0.845416698 | 0.002063979 | actived |
| C00182 | C08278 | -0.717011595 | 0.019607767 | inhibted |
| C01530 | C08278 | 0.75011757 | 0.012456351 | actived |
| C00141 | C05463 | -0.670791264 | 0.033740993 | inhibted |
| C00149 | C05463 | 0.655314901 | 0.039699247 | actived |
| C00785 | C05463 | -0.765351346 | 0.009881511 | inhibted |
| C01571 | C05463 | -0.661235433 | 0.037342451 | inhibted |
| C05607 | C05463 | -0.762746933 | 0.010292342 | inhibted |
| C08262 | C05463 | -0.685051574 | 0.028816341 | inhibted |
| C00109 | C00360 | 0.867789665 | 0.001136106 | actived |
| C00149 | C00360 | -0.76612303 | 0.009762036 | inhibted |
| C01571 | C00360 | 0.658023756 | 0.038608857 | actived |
| C00182 | C00360 | -0.70667788 | 0.022321395 | inhibted |
| C00109 | C00170 | 0.823083964 | 0.003441422 | actived |
| C00141 | C00170 | 0.752960523 | 0.011943773 | actived |
| C00149 | C00170 | -0.738575501 | 0.014696175 | inhibted |
| C00299 | C00170 | -0.672742972 | 0.033035589 | inhibted |
| C00182 | C00170 | -0.732906033 | 0.015893091 | inhibted |
| C08278 | C00170 | 0.692007866 | 0.026602595 | actived |
| C00360 | C00170 | 0.691359759 | 0.026803755 | actived |
| C00109 | C00016 | 0.703748413 | 0.023135354 | actived |
| C00141 | C00016 | 0.675389183 | 0.032095255 | actived |
| C00149 | C00016 | -0.647855375 | 0.042808619 | inhibted |
| C02571 | C00016 | 0.811240094 | 0.004392343 | actived |
| C01571 | C00016 | 0.661448181 | 0.037259566 | actived |
| C00182 | C00016 | -0.675489705 | 0.032059897 | inhibted |
| C08278 | C00016 | 0.721802606 | 0.018430816 | actived |
| C00360 | C00016 | 0.756246337 | 0.011369993 | actived |
| C00170 | C00016 | 0.799624069 | 0.005494464 | actived |
| C00109 | C00612 | 0.759431607 | 0.010832521 | actived |
| C00149 | C00612 | -0.736929754 | 0.015036905 | inhibted |
| C00081 | C00612 | 0.638331595 | 0.047010447 | actived |
| C02571 | C00612 | 0.638812291 | 0.046792032 | actived |
| C01571 | C00612 | 0.710450213 | 0.021302591 | actived |
| C00182 | C00612 | -0.87617405 | 0.000883461 | inhibted |
| C00360 | C00612 | 0.828119358 | 0.00308588 | actived |
| C00170 | C00612 | 0.76445315 | 0.010021866 | actived |
| C00016 | C00612 | 0.711446278 | 0.021039035 | actived |
| C00109 | C00390 | 0.878907339 | 0.00081078 | actived |
| C00141 | C00390 | 0.680149407 | 0.030449807 | actived |
| C00149 | C00390 | -0.841809231 | 0.002253129 | inhibted |
| C00082 | C00390 | -0.65102089 | 0.041469873 | inhibted |
| C00081 | C00390 | 0.827854801 | 0.003103871 | actived |
| C01571 | C00390 | 0.713462292 | 0.020512506 | actived |
| C08262 | C00390 | 0.72505462 | 0.017660426 | actived |
| C00182 | C00390 | -0.763393946 | 0.010189178 | inhibted |
| C05463 | C00390 | -0.673682329 | 0.032699675 | inhibted |
| C00360 | C00390 | 0.8030003 | 0.005155867 | actived |
| C00170 | C00390 | 0.741362188 | 0.014131571 | actived |
| C00612 | C00390 | 0.876783962 | 0.000866847 | actived |
| C00109 | C01107 | 0.909200028 | 0.000266193 | actived |
| C00141 | C01107 | 0.663310067 | 0.036539486 | actived |
| C00149 | C01107 | -0.709356811 | 0.021594516 | inhibted |
| C00082 | C01107 | -0.684217758 | 0.029089854 | inhibted |
| C00081 | C01107 | 0.676735029 | 0.03162406 | actived |
| C00182 | C01107 | -0.748193941 | 0.012811795 | inhibted |
| C00360 | C01107 | 0.904229826 | 0.000327411 | actived |
| C00170 | C01107 | 0.666548611 | 0.035309503 | actived |
| C00612 | C01107 | 0.86538106 | 0.001217482 | actived |
| C00390 | C01107 | 0.905386656 | 0.000312325 | actived |
| C00109 | C00666 | 0.728282973 | 0.016918036 | actived |
| C00149 | C00666 | -0.634430184 | 0.048808356 | inhibted |
| C00081 | C00666 | 0.678892665 | 0.030878504 | actived |
| C02571 | C00666 | 0.660194136 | 0.037749927 | actived |
| C01571 | C00666 | 0.71969899 | 0.018941377 | actived |
| C00182 | C00666 | -0.892124727 | 0.000519168 | inhibted |
| C00360 | C00666 | 0.78387033 | 0.007286502 | actived |
| C00170 | C00666 | 0.697676965 | 0.024886896 | actived |
| C00016 | C00666 | 0.739653887 | 0.014475853 | actived |
| C00612 | C00666 | 0.935169697 | 7.14E-05 | actived |
| C00390 | C00666 | 0.83217132 | 0.002819594 | actived |
| C01107 | C00666 | 0.854318693 | 0.001646561 | actived |
| C00109 | C02934 | 0.953146187 | 1.99E-05 | actived |
| C00141 | C02934 | 0.794819035 | 0.006003274 | actived |
| C00149 | C02934 | -0.774723806 | 0.008498477 | inhibted |
| C00182 | C02934 | -0.664728961 | 0.03599709 | inhibted |
| C00360 | C02934 | 0.9080109 | 0.00028 | actived |
| C00170 | C02934 | 0.799134518 | 0.005544842 | actived |
| C00016 | C02934 | 0.725866433 | 0.017471656 | actived |
| C00612 | C02934 | 0.86105374 | 0.001374206 | actived |
| C00390 | C02934 | 0.878411674 | 0.000823624 | actived |
| C01107 | C02934 | 0.940715521 | 5.03E-05 | actived |
| C00666 | C02934 | 0.803464731 | 0.005110485 | actived |
| C00149 | C02632 | -0.690438152 | 0.027091593 | inhibted |
| C02571 | C02632 | 0.664219027 | 0.036191389 | actived |
| C05476 | C02632 | 0.851132087 | 0.001788184 | actived |
| C01571 | C02632 | 0.653941734 | 0.040259822 | actived |
| C05607 | C02632 | 0.755655733 | 0.011471668 | actived |
| C01530 | C02632 | 0.742178736 | 0.013969045 | actived |
| C08278 | C02632 | 0.687583417 | 0.0279966 | actived |
| C05463 | C02632 | -0.637035222 | 0.047602871 | inhibted |
| C00182 | C02378 | -0.822884344 | 0.003456096 | inhibted |
| C00170 | C02378 | 0.785888881 | 0.007036545 | actived |
| C00612 | C02378 | 0.652726634 | 0.040760289 | actived |
| C00390 | C02378 | 0.645043243 | 0.04402197 | actived |
| C00666 | C02378 | 0.726028669 | 0.0174341 | actived |
| C00141 | C14811 | -0.650536539 | 0.041672864 | inhibted |
| C00082 | C14811 | 0.742017174 | 0.014001098 | actived |
| C05476 | C14811 | -0.725345171 | 0.017592703 | inhibted |
| C05607 | C14811 | -0.720564598 | 0.018730118 | inhibted |
| C08262 | C14811 | -0.722593049 | 0.018241462 | inhibted |
| C01530 | C14811 | -0.807865653 | 0.004694485 | inhibted |
| C00109 | C03345 | 0.639950529 | 0.046277556 | actived |
| C00149 | C03345 | -0.641334608 | 0.04565707 | inhibted |
| C00081 | C03345 | 0.802228599 | 0.005231909 | actived |
| C01571 | C03345 | 0.805555347 | 0.004909723 | actived |
| C00182 | C03345 | -0.759178512 | 0.010874561 | inhibted |
| C00360 | C03345 | 0.737510939 | 0.014915956 | actived |
| C00612 | C03345 | 0.89650881 | 0.000442191 | actived |
| C00390 | C03345 | 0.856828559 | 0.00154088 | actived |
| C01107 | C03345 | 0.849508423 | 0.001863638 | actived |
| C00666 | C03345 | 0.884170847 | 0.000683235 | actived |
| C02934 | C03345 | 0.752240801 | 0.012072112 | actived |
| C00109 | C01802 | -0.675194764 | 0.032163715 | inhibted |
| C00141 | C01802 | -0.761502088 | 0.010492893 | inhibted |
| C00299 | C01802 | 0.845242276 | 0.00207285 | actived |
| C00785 | C01802 | -0.795990188 | 0.005876293 | inhibted |
| C08262 | C01802 | -0.890780809 | 0.000544611 | inhibted |
| C05463 | C01802 | 0.676851959 | 0.031583345 | actived |
| C00170 | C01802 | -0.673544606 | 0.032748779 | inhibted |
| C00390 | C01802 | -0.647036764 | 0.043159481 | inhibted |
| C14811 | C01802 | 0.66989147 | 0.034069618 | actived |
| C00109 | C02165 | 0.687340413 | 0.028074578 | actived |
| C00149 | C02165 | -0.742789668 | 0.013848303 | inhibted |
| C00081 | C02165 | 0.662263217 | 0.036943184 | actived |
| C02571 | C02165 | 0.794784349 | 0.006007064 | actived |
| C01571 | C02165 | 0.850072473 | 0.001837169 | actived |
| C00182 | C02165 | -0.82851998 | 0.003058778 | inhibted |
| C08278 | C02165 | 0.68071965 | 0.030256632 | actived |
| C00360 | C02165 | 0.799214524 | 0.005536586 | actived |
| C00170 | C02165 | 0.645716784 | 0.043729285 | actived |
| C00016 | C02165 | 0.780877736 | 0.007668568 | actived |
| C00612 | C02165 | 0.924125925 | 0.000132206 | actived |
| C00390 | C02165 | 0.82878287 | 0.003041086 | actived |
| C01107 | C02165 | 0.806725258 | 0.004799868 | actived |
| C00666 | C02165 | 0.920504347 | 0.000158602 | actived |
| C02934 | C02165 | 0.799262763 | 0.005531613 | actived |
| C02632 | C02165 | 0.72273786 | 0.018206918 | actived |
| C03345 | C02165 | 0.919701018 | 0.000164947 | actived |
| C00082 | C15589 | 0.633388881 | 0.049295856 | actived |
| C05476 | C15589 | -0.675100443 | 0.032196963 | inhibted |
| C00785 | C15589 | -0.791387509 | 0.006386568 | inhibted |
| C08262 | C15589 | -0.781346945 | 0.007607747 | inhibted |
| C01530 | C15589 | -0.680510364 | 0.030327432 | inhibted |
| C02632 | C15589 | -0.687410146 | 0.028052186 | inhibted |
| C14811 | C15589 | 0.641079365 | 0.045771075 | actived |
| C01802 | C15589 | 0.709742443 | 0.021491244 | actived |
| C00109 | C08842 | 0.695422196 | 0.025559898 | actived |
| C00141 | C08842 | 0.828482776 | 0.003061287 | actived |
| C00299 | C08842 | -0.700512341 | 0.024057989 | inhibted |
| C00785 | C08842 | 0.680331396 | 0.030388066 | actived |
| C00170 | C08842 | 0.848057359 | 0.001932997 | actived |
| C00016 | C08842 | 0.67570551 | 0.031984079 | actived |
| C02934 | C08842 | 0.633745441 | 0.049128565 | actived |
| C02378 | C08842 | 0.713844697 | 0.02041367 | actived |
| C14811 | C08842 | -0.702574023 | 0.023467318 | inhibted |
| C01802 | C08842 | -0.762228635 | 0.010375511 | inhibted |
| C15589 | C08842 | -0.679440101 | 0.030691262 | inhibted |
| C00149 | C00521 | -0.769329847 | 0.009276435 | inhibted |
| C00081 | C00521 | 0.677470039 | 0.031368723 | actived |
| C02571 | C00521 | 0.778852613 | 0.007935028 | actived |
| C00182 | C00521 | -0.825533352 | 0.003264995 | inhibted |
| C08278 | C00521 | 0.808907207 | 0.00459969 | actived |
| C00170 | C00521 | 0.797099168 | 0.005757827 | actived |
| C00612 | C00521 | 0.720402451 | 0.018769566 | actived |
| C00390 | C00521 | 0.774822626 | 0.008484671 | actived |
| C00666 | C00521 | 0.694994231 | 0.02568903 | actived |
| C02378 | C00521 | 0.804542573 | 0.005006263 | actived |
| C02165 | C00521 | 0.685443434 | 0.02868841 | actived |
| C15589 | C00521 | -0.657426998 | 0.038847312 | inhibted |
| C08842 | C00521 | 0.671721108 | 0.033403659 | actived |
| C00299 | C00823 | -0.6539672 | 0.040249377 | inhibted |
| C05607 | C00823 | 0.680892207 | 0.030198341 | actived |
| C08262 | C00823 | 0.801733941 | 0.005281071 | actived |
| C01530 | C00823 | 0.80358535 | 0.005098746 | actived |
| C08278 | C00823 | 0.799384512 | 0.005519075 | actived |
| C14811 | C00823 | -0.704355627 | 0.022964988 | inhibted |
| C01802 | C00823 | -0.735238774 | 0.015392698 | inhibted |
| C00521 | C00823 | 0.665248271 | 0.035799944 | actived |
| C00141 | C02576 | -0.7657127 | 0.009825438 | inhibted |
| C00299 | C02576 | 0.684826099 | 0.028890129 | actived |
| C14811 | C02576 | 0.75681877 | 0.011272052 | actived |
| C00149 | C04056 | -0.784941144 | 0.007153133 | inhibted |
| C02571 | C04056 | 0.729549619 | 0.016632779 | actived |
| C00182 | C04056 | -0.770854248 | 0.009051685 | inhibted |
| C08278 | C04056 | 0.683188653 | 0.029429863 | actived |
| C00170 | C04056 | 0.855961325 | 0.001576822 | actived |
| C00016 | C04056 | 0.664820505 | 0.035962284 | actived |
| C00612 | C04056 | 0.773449587 | 0.008677928 | actived |
| C00390 | C04056 | 0.729281166 | 0.016692955 | actived |
| C00666 | C04056 | 0.687138452 | 0.028139498 | actived |
| C02378 | C04056 | 0.729353189 | 0.016676796 | actived |
| C02165 | C04056 | 0.656510046 | 0.039215645 | actived |
| C00521 | C04056 | 0.926879295 | 0.000114423 | actived |
| C00109 | C16794 | 0.934147175 | 7.60E-05 | actived |
| C00141 | C16794 | 0.850614786 | 0.001811979 | actived |
| C00149 | C16794 | -0.781674279 | 0.007565519 | inhibted |
| C00299 | C16794 | -0.866433938 | 0.001181406 | inhibted |
| C08262 | C16794 | 0.705876777 | 0.022541992 | actived |
| C05463 | C16794 | -0.713397036 | 0.020529405 | inhibted |
| C00360 | C16794 | 0.736676062 | 0.015089914 | actived |
| C00170 | C16794 | 0.839857227 | 0.002360538 | actived |
| C00612 | C16794 | 0.656785495 | 0.039104753 | actived |
| C00390 | C16794 | 0.85538789 | 0.001600919 | actived |
| C01107 | C16794 | 0.766213097 | 0.009748159 | actived |
| C02934 | C16794 | 0.849191448 | 0.001878633 | actived |
| C01802 | C16794 | -0.794355904 | 0.006054023 | inhibted |
| C08842 | C16794 | 0.686056862 | 0.028488922 | actived |
| C00521 | C16794 | 0.65943957 | 0.03804707 | actived |
| C04056 | C16794 | 0.682361057 | 0.029705252 | actived |
| C00109 | C13856 | 0.714878519 | 0.020148117 | actived |
| C00149 | C13856 | -0.650511501 | 0.041683375 | inhibted |
| C00081 | C13856 | 0.828167074 | 0.003082643 | actived |
| C01571 | C13856 | 0.715562093 | 0.019973847 | actived |
| C00182 | C13856 | -0.779702687 | 0.007822393 | inhibted |
| C00360 | C13856 | 0.777281161 | 0.008146262 | actived |
| C00612 | C13856 | 0.838620724 | 0.002430458 | actived |
| C00390 | C13856 | 0.891681964 | 0.000527452 | actived |
| C01107 | C13856 | 0.890885114 | 0.000542605 | actived |
| C00666 | C13856 | 0.860666394 | 0.001388914 | actived |
| C02934 | C13856 | 0.739162897 | 0.014575878 | actived |
| C03345 | C13856 | 0.903186041 | 0.000341476 | actived |
| C02165 | C13856 | 0.777293261 | 0.00814462 | actived |
| C16794 | C13856 | 0.632598996 | 0.049667807 | actived |
| C00149 | C14717 | -0.742647411 | 0.013876353 | inhibted |
| C00081 | C14717 | 0.863718669 | 0.001276063 | actived |
| C02571 | C14717 | 0.691731591 | 0.026688219 | actived |
| C01571 | C14717 | 0.828884831 | 0.003034245 | actived |
| C00182 | C14717 | -0.844874274 | 0.002091658 | inhibted |
| C08278 | C14717 | 0.632751465 | 0.049595866 | actived |
| C00360 | C14717 | 0.673212417 | 0.032867424 | actived |
| C00612 | C14717 | 0.88024651 | 0.000776809 | actived |
| C00390 | C14717 | 0.876114115 | 0.000885106 | actived |
| C01107 | C14717 | 0.768627835 | 0.009381249 | actived |
| C00666 | C14717 | 0.846850207 | 0.001992104 | actived |
| C02934 | C14717 | 0.663892341 | 0.036316238 | actived |
| C03345 | C14717 | 0.935792571 | 6.88E-05 | actived |
| C02165 | C14717 | 0.89410411 | 0.000483293 | actived |
| C00521 | C14717 | 0.782781028 | 0.007423973 | actived |
| C04056 | C14717 | 0.706075206 | 0.022487212 | actived |
| C13856 | C14717 | 0.884333528 | 0.000679543 | actived |
| C05476 | C05938 | -0.676237725 | 0.031797619 | inhibted |
| C00785 | C05938 | -0.794557499 | 0.006031895 | inhibted |
| C08262 | C05938 | -0.679111292 | 0.030803633 | inhibted |
| C02632 | C05938 | -0.750986738 | 0.012298038 | inhibted |
| C15589 | C05938 | 0.883481256 | 0.000699047 | actived |
| C00521 | C05938 | -0.636938398 | 0.047647317 | inhibted |
| C00149 | C00157 | 0.739598945 | 0.014487022 | actived |
| C00081 | C00157 | -0.751135658 | 0.012271055 | inhibted |
| C02571 | C00157 | -0.735480015 | 0.015341584 | inhibted |
| C00785 | C00157 | -0.726124859 | 0.01741186 | inhibted |
| C01571 | C00157 | -0.663410734 | 0.036500823 | inhibted |
| C08262 | C00157 | -0.79823192 | 0.005638587 | inhibted |
| C01530 | C00157 | -0.712780753 | 0.020689475 | inhibted |
| C08278 | C00157 | -0.705800561 | 0.022563057 | inhibted |
| C00390 | C00157 | -0.675668255 | 0.031997159 | inhibted |
| C02632 | C00157 | -0.681394963 | 0.030028945 | inhibted |
| C01802 | C00157 | 0.678483592 | 0.031018927 | actived |
| C02165 | C00157 | -0.655788122 | 0.039507286 | inhibted |
| C15589 | C00157 | 0.832196964 | 0.002817963 | actived |
| C00521 | C00157 | -0.799676716 | 0.005489066 | inhibted |
| C00823 | C00157 | -0.718927932 | 0.019130945 | inhibted |
| C04056 | C00157 | -0.660145625 | 0.037768983 | inhibted |
| C14717 | C00157 | -0.737416757 | 0.01493551 | inhibted |
| C05938 | C00157 | 0.715211621 | 0.020063065 | actived |
| C00109 | C04230 | -0.712635374 | 0.02072736 | inhibted |
| C00141 | C04230 | -0.709707358 | 0.021500626 | inhibted |
| C00182 | C04230 | 0.66060542 | 0.037588629 | actived |
| C00360 | C04230 | -0.723980151 | 0.017912442 | inhibted |
| C00016 | C04230 | -0.711929085 | 0.020912099 | inhibted |
| C02934 | C04230 | -0.639314598 | 0.046564524 | inhibted |
| C02576 | C04230 | 0.696613145 | 0.02520289 | actived |
| C00109 | C04692 | 0.798793874 | 0.00558009 | actived |
| C00149 | C04692 | -0.883904375 | 0.000689314 | inhibted |
| C00081 | C04692 | 0.712598627 | 0.020736944 | actived |
| C02571 | C04692 | 0.786662371 | 0.006942397 | actived |
| C01571 | C04692 | 0.796370528 | 0.00583547 | actived |
| C08262 | C04692 | 0.643053696 | 0.044894179 | actived |
| C00182 | C04692 | -0.870243757 | 0.001057337 | inhibted |
| C01530 | C04692 | 0.658907594 | 0.038257503 | actived |
| C08278 | C04692 | 0.813405572 | 0.00420594 | actived |
| C00360 | C04692 | 0.83979203 | 0.002364188 | actived |
| C00170 | C04692 | 0.776978371 | 0.008187415 | actived |
| C00016 | C04692 | 0.754467183 | 0.011678213 | actived |
| C00612 | C04692 | 0.910950639 | 0.000246786 | actived |
| C00390 | C04692 | 0.921180893 | 0.000153399 | actived |
| C01107 | C04692 | 0.842476325 | 0.002217245 | actived |
| C00666 | C04692 | 0.855770157 | 0.001584826 | actived |
| C02934 | C04692 | 0.835829694 | 0.002593758 | actived |
| C02632 | C04692 | 0.704391237 | 0.022955023 | actived |
| C02378 | C04692 | 0.655648883 | 0.039563703 | actived |
| C03345 | C04692 | 0.845959886 | 0.002036527 | actived |
| C02165 | C04692 | 0.928917279 | 0.000102447 | actived |
| C00521 | C04692 | 0.840036654 | 0.002350514 | actived |
| C04056 | C04692 | 0.791838725 | 0.006335202 | actived |
| C16794 | C04692 | 0.723579154 | 0.018007132 | actived |
| C13856 | C04692 | 0.804669944 | 0.004994048 | actived |
| C14717 | C04692 | 0.911143308 | 0.000244716 | actived |
| C05938 | C04692 | -0.63707872 | 0.047582913 | inhibted |
| C00157 | C04692 | -0.724520266 | 0.017785449 | inhibted |
| C00109 | C09821 | 0.706705665 | 0.022313771 | actived |
| C00141 | C09821 | 0.866308941 | 0.001185648 | actived |
| C05607 | C09821 | 0.760326934 | 0.010684724 | actived |
| C00170 | C09821 | 0.688906409 | 0.027574657 | actived |
| C00016 | C09821 | 0.674993454 | 0.032234706 | actived |
| C02934 | C09821 | 0.653789624 | 0.040322244 | actived |
| C08842 | C09821 | 0.767352755 | 0.009573755 | actived |
| C02576 | C09821 | -0.676054021 | 0.031861896 | inhibted |
| C16794 | C09821 | 0.6405499 | 0.046008169 | actived |
| C04230 | C09821 | -0.81676866 | 0.003927773 | inhibted |
| C00109 | C04421 | -0.650034458 | 0.041883989 | inhibted |
| C00141 | C04421 | -0.706042412 | 0.022496259 | inhibted |
| C00081 | C04421 | -0.63736005 | 0.047453964 | inhibted |
| C00299 | C04421 | 0.716363957 | 0.019770753 | actived |
| C05607 | C04421 | -0.739060029 | 0.014596895 | inhibted |
| C08262 | C04421 | -0.77810323 | 0.00803527 | inhibted |
| C01530 | C04421 | -0.780777869 | 0.007681557 | inhibted |
| C08278 | C04421 | -0.727828096 | 0.017021302 | inhibted |
| C05463 | C04421 | 0.70022787 | 0.024140284 | actived |
| C00390 | C04421 | -0.721667142 | 0.018463403 | inhibted |
| C14811 | C04421 | 0.844589995 | 0.002106271 | actived |
| C01802 | C04421 | 0.739640465 | 0.014478581 | actived |
| C08842 | C04421 | -0.695693737 | 0.025478195 | inhibted |
| C00521 | C04421 | -0.681323049 | 0.030053135 | inhibted |
| C00823 | C04421 | -0.89408552 | 0.000483621 | inhibted |
| C02576 | C04421 | 0.782234999 | 0.00749357 | actived |
| C16794 | C04421 | -0.664053429 | 0.036254639 | inhibted |
| C00157 | C04421 | 0.684280918 | 0.029069075 | actived |
| C04692 | C04421 | -0.666667809 | 0.035264775 | inhibted |
| C00109 | C03577 | -0.881105462 | 0.000755575 | inhibted |
| C00141 | C03577 | -0.689941687 | 0.027247524 | inhibted |
| C00299 | C03577 | 0.679028925 | 0.030831826 | actived |
| C00182 | C03577 | 0.697982957 | 0.024796512 | actived |
| C00360 | C03577 | -0.82948582 | 0.002994143 | inhibted |
| C00612 | C03577 | -0.697929857 | 0.02481218 | inhibted |
| C00390 | C03577 | -0.832267325 | 0.002813493 | inhibted |
| C01107 | C03577 | -0.890105774 | 0.00055773 | inhibted |
| C00666 | C03577 | -0.720206594 | 0.018817292 | inhibted |
| C02934 | C03577 | -0.790098276 | 0.006534964 | inhibted |
| C03345 | C03577 | -0.673536164 | 0.03275179 | inhibted |
| C02576 | C03577 | 0.703864664 | 0.023102671 | actived |
| C16794 | C03577 | -0.794973603 | 0.005986404 | inhibted |
| C13856 | C03577 | -0.856082357 | 0.00157177 | inhibted |
| C14717 | C03577 | -0.633883993 | 0.04906366 | inhibted |
| C04230 | C03577 | 0.757050182 | 0.011232628 | actived |
| C04692 | C03577 | -0.717922313 | 0.019380149 | inhibted |
| C00149 | C20157 | -0.764127968 | 0.010073024 | inhibted |
| C00081 | C20157 | 0.645864719 | 0.043665176 | actived |
| C02571 | C20157 | 0.875320948 | 0.000907087 | actived |
| C01571 | C20157 | 0.869496604 | 0.001080883 | actived |
| C00182 | C20157 | -0.903132842 | 0.000342205 | inhibted |
| C08278 | C20157 | 0.754342102 | 0.011700101 | actived |
| C00360 | C20157 | 0.774135775 | 0.008580962 | actived |
| C00170 | C20157 | 0.64265435 | 0.045070633 | actived |
| C00016 | C20157 | 0.753432563 | 0.011860122 | actived |
| C00612 | C20157 | 0.876052477 | 0.0008868 | actived |
| C00390 | C20157 | 0.782316335 | 0.007483174 | actived |
| C01107 | C20157 | 0.722653017 | 0.018227151 | actived |
| C00666 | C20157 | 0.883085006 | 0.000708253 | actived |
| C02934 | C20157 | 0.670232968 | 0.033944641 | actived |
| C03345 | C20157 | 0.833662409 | 0.002725906 | actived |
| C02165 | C20157 | 0.933103198 | 8.08E-05 | actived |
| C00521 | C20157 | 0.800612773 | 0.005393715 | actived |
| C04056 | C20157 | 0.768228179 | 0.009441291 | actived |
| C13856 | C20157 | 0.787260814 | 0.006870171 | actived |
| C14717 | C20157 | 0.90829442 | 0.000276662 | actived |
| C00157 | C20157 | -0.646137835 | 0.043546983 | inhibted |
| C04692 | C20157 | 0.930440223 | 9.41E-05 | actived |
| C00141 | C07339 | -0.648058349 | 0.04272192 | inhibted |
| C00082 | C07339 | 0.634232749 | 0.04890054 | actived |
| C14811 | C07339 | 0.835634179 | 0.002605486 | actived |
| C00823 | C07339 | -0.651634143 | 0.041213814 | inhibted |
| C02576 | C07339 | 0.9437439 | 4.09E-05 | actived |
| C04230 | C07339 | 0.636747374 | 0.047735086 | actived |
| C04421 | C07339 | 0.891861547 | 0.000524081 | actived |
| C03577 | C07339 | 0.67026342 | 0.033933512 | actived |
| C00149 | C06971 | -0.633730234 | 0.049135692 | inhibted |
| C00081 | C06971 | 0.643080062 | 0.044882545 | actived |
| C00299 | C06971 | -0.762546944 | 0.010324378 | inhibted |
| C08262 | C06971 | 0.635539263 | 0.04829267 | actived |
| C00182 | C06971 | -0.64351771 | 0.044689732 | inhibted |
| C00170 | C06971 | 0.792180726 | 0.006296465 | actived |
| C00390 | C06971 | 0.731341145 | 0.016235049 | actived |
| C02378 | C06971 | 0.824390579 | 0.003346478 | actived |
| C01802 | C06971 | -0.780237927 | 0.007752056 | inhibted |
| C08842 | C06971 | 0.758482125 | 0.010990823 | actived |
| C00521 | C06971 | 0.899161024 | 0.000399895 | actived |
| C00823 | C06971 | 0.643556701 | 0.044672581 | actived |
| C04056 | C06971 | 0.828534606 | 0.003057791 | actived |
| C16794 | C06971 | 0.76688057 | 0.009645745 | actived |
| C00157 | C06971 | -0.67728799 | 0.031431834 | inhibted |
| C04692 | C06971 | 0.649027144 | 0.042309729 | actived |
| C04421 | C06971 | -0.701952291 | 0.023644381 | inhibted |
| C00109 | C01425 | 0.771237335 | 0.008995815 | actived |
| C00141 | C01425 | 0.645476146 | 0.043833704 | actived |
| C00149 | C01425 | -0.701561146 | 0.023756245 | inhibted |
| C02571 | C01425 | 0.738820728 | 0.014645869 | actived |
| C01571 | C01425 | 0.650689082 | 0.041608861 | actived |
| C00182 | C01425 | -0.703590829 | 0.02317971 | inhibted |
| C08278 | C01425 | 0.656018763 | 0.039413954 | actived |
| C00360 | C01425 | 0.881283556 | 0.000751225 | actived |
| C00170 | C01425 | 0.796494392 | 0.005822219 | actived |
| C00016 | C01425 | 0.964711264 | 6.50E-06 | actived |
| C00612 | C01425 | 0.760675113 | 0.010627632 | actived |
| C00390 | C01425 | 0.659658794 | 0.037960579 | actived |
| C01107 | C01425 | 0.706386091 | 0.022401571 | actived |
| C00666 | C01425 | 0.775529653 | 0.008386352 | actived |
| C02934 | C01425 | 0.803660368 | 0.005091454 | actived |
| C02165 | C01425 | 0.787682249 | 0.00681963 | actived |
| C04056 | C01425 | 0.660850331 | 0.0374928 | actived |
| C16794 | C01425 | 0.665852519 | 0.035571474 | actived |
| C04230 | C01425 | -0.729875395 | 0.016559956 | inhibted |
| C04692 | C01425 | 0.782965967 | 0.007400505 | actived |
| C03577 | C01425 | -0.638654682 | 0.04686357 | inhibted |
| C20157 | C01425 | 0.780653462 | 0.00769776 | actived |
| C00149 | C11271 | -0.676060827 | 0.031859513 | inhibted |
| C02571 | C11271 | 0.731084455 | 0.016291626 | actived |
| C00182 | C11271 | -0.824495629 | 0.003338928 | inhibted |
| C08278 | C11271 | 0.780763062 | 0.007683484 | actived |
| C00170 | C11271 | 0.843957258 | 0.00213906 | actived |
| C00016 | C11271 | 0.685340945 | 0.028721832 | actived |
| C00612 | C11271 | 0.805280873 | 0.004935754 | actived |
| C00390 | C11271 | 0.701497242 | 0.023774556 | actived |
| C00666 | C11271 | 0.679340208 | 0.030725371 | actived |
| C02378 | C11271 | 0.678500552 | 0.031013097 | actived |
| C02165 | C11271 | 0.701940231 | 0.023647825 | actived |
| C15589 | C11271 | -0.633687456 | 0.049155744 | inhibted |
| C08842 | C11271 | 0.714597346 | 0.020220102 | actived |
| C00521 | C11271 | 0.889139481 | 0.000576909 | actived |
| C04056 | C11271 | 0.929582124 | 9.87E-05 | actived |
| C14717 | C11271 | 0.719853776 | 0.018903479 | actived |
| C05938 | C11271 | -0.760120119 | 0.010718737 | inhibted |
| C00157 | C11271 | -0.636391109 | 0.047899066 | inhibted |
| C04692 | C11271 | 0.808621092 | 0.004625593 | actived |
| C20157 | C11271 | 0.766871855 | 0.009647077 | actived |
| C06971 | C11271 | 0.753690161 | 0.011814646 | actived |
| C01425 | C11271 | 0.643299237 | 0.044785914 | actived |
| C00149 | C14147 | -0.736219939 | 0.015185549 | inhibted |
| C00081 | C14147 | 0.801615123 | 0.005292928 | actived |
| C02571 | C14147 | 0.75212198 | 0.012093392 | actived |
| C01571 | C14147 | 0.82988982 | 0.002967401 | actived |
| C00182 | C14147 | -0.885658765 | 0.000650009 | inhibted |
| C08278 | C14147 | 0.683353428 | 0.029375241 | actived |
| C00360 | C14147 | 0.671487534 | 0.03348818 | actived |
| C00612 | C14147 | 0.87488744 | 0.000919268 | actived |
| C00390 | C14147 | 0.829323021 | 0.003004968 | actived |
| C01107 | C14147 | 0.725705933 | 0.017508865 | actived |
| C00666 | C14147 | 0.843717724 | 0.002151569 | actived |
| C03345 | C14147 | 0.895506401 | 0.000459 | actived |
| C02165 | C14147 | 0.891129483 | 0.000537925 | actived |
| C00521 | C14147 | 0.812953956 | 0.004244339 | actived |
| C04056 | C14147 | 0.745560794 | 0.013309773 | actived |
| C13856 | C14147 | 0.849773836 | 0.001851149 | actived |
| C14717 | C14147 | 0.988293378 | 8.10E-08 | actived |
| C00157 | C14147 | -0.717572429 | 0.019467379 | inhibted |
| C04692 | C14147 | 0.913708451 | 0.000218351 | actived |
| C20157 | C14147 | 0.945713898 | 3.56E-05 | actived |
| C11271 | C14147 | 0.761218379 | 0.010538981 | actived |
| C00109 | C17145 | -0.721899464 | 0.01840754 | inhibted |
| C00141 | C17145 | -0.702611017 | 0.023456811 | inhibted |
| C00149 | C17145 | 0.709677944 | 0.021508493 | actived |
| C01571 | C17145 | -0.752333508 | 0.012055526 | inhibted |
| C05463 | C17145 | 0.90908774 | 0.000267475 | actived |
| C00360 | C17145 | -0.699313947 | 0.024405982 | inhibted |
| C00170 | C17145 | -0.67584212 | 0.031936148 | inhibted |
| C00016 | C17145 | -0.723417426 | 0.01804542 | inhibted |
| C00612 | C17145 | -0.692233469 | 0.026532816 | inhibted |
| C00390 | C17145 | -0.778221314 | 0.008019415 | inhibted |
| C02934 | C17145 | -0.698256874 | 0.024715793 | inhibted |
| C02165 | C17145 | -0.660465342 | 0.037643513 | inhibted |
| C04056 | C17145 | -0.692241559 | 0.026530316 | inhibted |
| C16794 | C17145 | -0.786717409 | 0.006935732 | inhibted |
| C04230 | C17145 | 0.6467614 | 0.043277935 | actived |
| C04692 | C17145 | -0.72627785 | 0.017376526 | inhibted |
| C09821 | C17145 | -0.677631711 | 0.031312748 | inhibted |
| C04421 | C17145 | 0.655571684 | 0.039595005 | actived |
| C03577 | C17145 | 0.649039669 | 0.042304418 | actived |
| C01425 | C17145 | -0.707916179 | 0.021983344 | inhibted |
| C11271 | C17145 | -0.694793618 | 0.025749716 | inhibted |
| C02571 | C09981 | 0.761193512 | 0.010543028 | actived |
| C01530 | C09981 | 0.774670345 | 0.008505953 | actived |
| C08278 | C09981 | 0.905850072 | 0.000306427 | actived |
| C02632 | C09981 | 0.731694023 | 0.016157496 | actived |
| C15589 | C09981 | -0.789666109 | 0.006585252 | inhibted |
| C00521 | C09981 | 0.701981578 | 0.02363602 | actived |
| C00823 | C09981 | 0.683982303 | 0.029167409 | actived |
| C05938 | C09981 | -0.686006188 | 0.028505365 | inhibted |
| C00157 | C09981 | -0.740385422 | 0.014327715 | inhibted |
| C04692 | C09981 | 0.702493645 | 0.023490157 | actived |
| C20157 | C09981 | 0.633813886 | 0.049096494 | actived |
| C11271 | C09981 | 0.687829419 | 0.02791781 | actived |
| C00109 | C14467 | 0.907696216 | 0.00028374 | actived |
| C00141 | C14467 | 0.731374522 | 0.016227703 | actived |
| C00149 | C14467 | -0.713346447 | 0.020542512 | inhibted |
| C00082 | C14467 | -0.6959257 | 0.025408543 | inhibted |
| C00081 | C14467 | 0.738512081 | 0.014709204 | actived |
| C00299 | C14467 | -0.643327803 | 0.04477333 | inhibted |
| C00182 | C14467 | -0.832303249 | 0.002811213 | inhibted |
| C00360 | C14467 | 0.80806115 | 0.004676587 | actived |
| C00170 | C14467 | 0.796693004 | 0.005801016 | actived |
| C00016 | C14467 | 0.638057898 | 0.04713511 | actived |
| C00612 | C14467 | 0.910548637 | 0.000251147 | actived |
| C00390 | C14467 | 0.954038426 | 1.85E-05 | actived |
| C01107 | C14467 | 0.941343788 | 4.82E-05 | actived |
| C00666 | C14467 | 0.895330642 | 0.000461994 | actived |
| C02934 | C14467 | 0.908728144 | 0.000271611 | actived |
| C02378 | C14467 | 0.75747127 | 0.011161137 | actived |
| C03345 | C14467 | 0.834536902 | 0.002672015 | actived |
| C02165 | C14467 | 0.81346151 | 0.004201202 | actived |
| C08842 | C14467 | 0.70288634 | 0.023378718 | actived |
| C00521 | C14467 | 0.727830161 | 0.017020832 | actived |
| C04056 | C14467 | 0.700187774 | 0.024151899 | actived |
| C16794 | C14467 | 0.830777529 | 0.002909244 | actived |
| C13856 | C14467 | 0.880112841 | 0.000780153 | actived |
| C14717 | C14467 | 0.815176646 | 0.004057753 | actived |
| C04230 | C14467 | -0.646836222 | 0.043245727 | inhibted |
| C04692 | C14467 | 0.874980026 | 0.000916656 | actived |
| C04421 | C14467 | -0.648736781 | 0.042432987 | inhibted |
| C03577 | C14467 | -0.869265479 | 0.001088244 | inhibted |
| C20157 | C14467 | 0.748327184 | 0.012786949 | actived |
| C06971 | C14467 | 0.717834454 | 0.019402028 | actived |
| C01425 | C14467 | 0.691588275 | 0.02673271 | actived |
| C11271 | C14467 | 0.706846692 | 0.022275101 | actived |
| C14147 | C14467 | 0.776636348 | 0.008234077 | actived |
| C17145 | C14467 | -0.688758752 | 0.027621532 | inhibted |
| C02571 | C06559 | -0.761020338 | 0.010571237 | inhibted |
| C00182 | C06559 | 0.698371724 | 0.024682002 | actived |
| C01530 | C06559 | -0.647516438 | 0.042953656 | inhibted |
| C08278 | C06559 | -0.741682827 | 0.014067594 | inhibted |
| C02165 | C06559 | -0.682934585 | 0.02951422 | inhibted |
| C15589 | C06559 | 0.769760804 | 0.009212502 | actived |
| C00521 | C06559 | -0.774106664 | 0.00858506 | inhibted |
| C14717 | C06559 | -0.731489679 | 0.016202374 | inhibted |
| C00157 | C06559 | 0.838344223 | 0.002446296 | actived |
| C04692 | C06559 | -0.68347189 | 0.029336014 | inhibted |
| C20157 | C06559 | -0.713073654 | 0.020613291 | inhibted |
| C11271 | C06559 | -0.667077794 | 0.035111224 | inhibted |
| C14147 | C06559 | -0.761909053 | 0.010427029 | inhibted |
| C09981 | C06559 | -0.853995641 | 0.001660535 | inhibted |
| C00082 | C16856 | -0.664826467 | 0.035960018 | inhibted |
| C00081 | C16856 | 0.661337564 | 0.037302645 | actived |
| C01571 | C16856 | 0.637620938 | 0.047334594 | actived |
| C01530 | C16856 | 0.69391063 | 0.02601799 | actived |
| C14811 | C16856 | -0.658994037 | 0.038223256 | inhibted |
| C03345 | C16856 | 0.754388948 | 0.0116919 | actived |
| C02576 | C16856 | -0.681963392 | 0.029838201 | inhibted |
| C13856 | C16856 | 0.686069632 | 0.028484779 | actived |
| C14717 | C16856 | 0.701773885 | 0.023695359 | actived |
| C04421 | C16856 | -0.693760255 | 0.026063868 | inhibted |
| C03577 | C16856 | -0.6461617 | 0.043536666 | inhibted |
| C07339 | C16856 | -0.815334322 | 0.004044744 | inhibted |
| C14147 | C16856 | 0.652843492 | 0.040711977 | actived |
| C00109 | C08582 | 0.799513181 | 0.005505846 | actived |
| C00081 | C08582 | 0.642897437 | 0.044963168 | actived |
| C00182 | C08582 | -0.885914457 | 0.000644421 | inhibted |
| C00360 | C08582 | 0.774616473 | 0.008513491 | actived |
| C00170 | C08582 | 0.781762709 | 0.00755414 | actived |
| C00016 | C08582 | 0.679552756 | 0.030652827 | actived |
| C00612 | C08582 | 0.950073758 | 2.56E-05 | actived |
| C00390 | C08582 | 0.856841593 | 0.001540344 | actived |
| C01107 | C08582 | 0.883760706 | 0.000692608 | actived |
| C00666 | C08582 | 0.960351152 | 1.03E-05 | actived |
| C02934 | C08582 | 0.852304146 | 0.001735108 | actived |
| C02378 | C08582 | 0.781661347 | 0.007567184 | actived |
| C03345 | C08582 | 0.836343074 | 0.002563144 | actived |
| C02165 | C08582 | 0.84342028 | 0.002167175 | actived |
| C08842 | C08582 | 0.679916938 | 0.030528799 | actived |
| C00521 | C08582 | 0.694892056 | 0.025719926 | actived |
| C04056 | C08582 | 0.716210478 | 0.019809515 | actived |
| C16794 | C08582 | 0.686939242 | 0.028203635 | actived |
| C13856 | C08582 | 0.846373837 | 0.002015785 | actived |
| C14717 | C08582 | 0.798975141 | 0.005561314 | actived |
| C04230 | C08582 | -0.635045733 | 0.048521695 | inhibted |
| C04692 | C08582 | 0.82987631 | 0.002968292 | actived |
| C03577 | C08582 | -0.772210158 | 0.008855031 | inhibted |
| C20157 | C08582 | 0.78815762 | 0.006762937 | actived |
| C06971 | C08582 | 0.636439123 | 0.047876944 | actived |
| C01425 | C08582 | 0.726560176 | 0.017311454 | actived |
| C11271 | C08582 | 0.733301086 | 0.015807564 | actived |
| C14147 | C08582 | 0.784942071 | 0.007153018 | actived |
| C14467 | C08582 | 0.954187653 | 1.82E-05 | actived |
| C00109 | C08490 | 0.686455406 | 0.028359824 | actived |
| C00141 | C08490 | 0.755639332 | 0.011474501 | actived |
| C00149 | C08490 | -0.660186074 | 0.037753094 | inhibted |
| C02571 | C08490 | 0.680986977 | 0.03016636 | actived |
| C00299 | C08490 | -0.663977826 | 0.036283541 | inhibted |
| C00785 | C08490 | 0.650037463 | 0.041882724 | actived |
| C05607 | C08490 | 0.672174854 | 0.03323988 | actived |
| C08262 | C08490 | 0.658517295 | 0.038412393 | actived |
| C08278 | C08490 | 0.861217165 | 0.001368035 | actived |
| C05463 | C08490 | -0.71884677 | 0.019150975 | inhibted |
| C00170 | C08490 | 0.823457626 | 0.003414076 | actived |
| C00016 | C08490 | 0.740705294 | 0.014263273 | actived |
| C00390 | C08490 | 0.675110289 | 0.032193491 | actived |
| C01802 | C08490 | -0.735029259 | 0.015437186 | inhibted |
| C08842 | C08490 | 0.835559405 | 0.002609981 | actived |
| C00521 | C08490 | 0.794409411 | 0.006048144 | actived |
| C00823 | C08490 | 0.7917268 | 0.006347916 | actived |
| C04056 | C08490 | 0.734895792 | 0.015465572 | actived |
| C16794 | C08490 | 0.743030947 | 0.013800819 | actived |
| C05938 | C08490 | -0.71640413 | 0.019760616 | inhibted |
| C00157 | C08490 | -0.66455354 | 0.036063851 | inhibted |
| C04230 | C08490 | -0.651939854 | 0.041086564 | inhibted |
| C04692 | C08490 | 0.730436393 | 0.016435073 | actived |
| C09821 | C08490 | 0.802408236 | 0.005214137 | actived |
| C04421 | C08490 | -0.834876415 | 0.002651301 | inhibted |
| C06971 | C08490 | 0.760556379 | 0.010647077 | actived |
| C01425 | C08490 | 0.641473359 | 0.045595177 | actived |
| C11271 | C08490 | 0.822054788 | 0.003517556 | actived |
| C17145 | C08490 | -0.777820622 | 0.008073305 | inhibted |
| C09981 | C08490 | 0.717812597 | 0.019407473 | actived |
| C14467 | C08490 | 0.632506539 | 0.049711466 | actived |
| C00109 | C09183 | 0.679203602 | 0.030772058 | actived |
| C00141 | C09183 | 0.718445756 | 0.019250155 | actived |
| C05607 | C09183 | 0.687882148 | 0.027900942 | actived |
| C08278 | C09183 | 0.806959369 | 0.004778097 | actived |
| C00360 | C09183 | 0.636714754 | 0.047750085 | actived |
| C00170 | C09183 | 0.671820354 | 0.033367789 | actived |
| C00016 | C09183 | 0.78164848 | 0.007568841 | actived |
| C04230 | C09183 | -0.803121617 | 0.005143985 | inhibted |
| C09821 | C09183 | 0.83518455 | 0.002632601 | actived |
| C01425 | C09183 | 0.750550629 | 0.012377295 | actived |
| C09981 | C09183 | 0.662744154 | 0.036757347 | actived |
| C08490 | C09183 | 0.784223833 | 0.00724228 | actived |
| C00109 | C12127 | 0.825598645 | 0.003260383 | actived |
| C00141 | C12127 | 0.679408696 | 0.030701983 | actived |
| C00149 | C12127 | -0.675903575 | 0.031914601 | inhibted |
| C02571 | C12127 | 0.696851823 | 0.025131755 | actived |
| C01571 | C12127 | 0.699099964 | 0.02446848 | actived |
| C00182 | C12127 | -0.882364488 | 0.000725219 | inhibted |
| C00360 | C12127 | 0.819072339 | 0.00374501 | actived |
| C00170 | C12127 | 0.812396675 | 0.004292066 | actived |
| C00016 | C12127 | 0.84839655 | 0.001916619 | actived |
| C00612 | C12127 | 0.90512766 | 0.000315657 | actived |
| C00390 | C12127 | 0.851078733 | 0.001790627 | actived |
| C01107 | C12127 | 0.849315705 | 0.001872744 | actived |
| C00666 | C12127 | 0.967804934 | 4.52E-06 | actived |
| C02934 | C12127 | 0.847562729 | 0.00195706 | actived |
| C02378 | C12127 | 0.757602358 | 0.011138946 | actived |
| C03345 | C12127 | 0.796899414 | 0.005779039 | actived |
| C02165 | C12127 | 0.890825703 | 0.000543747 | actived |
| C08842 | C12127 | 0.710715575 | 0.021232156 | actived |
| C00521 | C12127 | 0.744020777 | 0.01360721 | actived |
| C04056 | C12127 | 0.732453162 | 0.015991531 | actived |
| C16794 | C12127 | 0.715904761 | 0.019886881 | actived |
| C13856 | C12127 | 0.801956776 | 0.005258884 | actived |
| C14717 | C12127 | 0.776193919 | 0.008294716 | actived |
| C04230 | C12127 | -0.696217018 | 0.025321254 | inhibted |
| C04692 | C12127 | 0.87295054 | 0.00097515 | actived |
| C03577 | C12127 | -0.774569873 | 0.008520015 | inhibted |
| C20157 | C12127 | 0.85768817 | 0.001505843 | actived |
| C06971 | C12127 | 0.644658186 | 0.044189884 | actived |
| C01425 | C12127 | 0.86434611 | 0.001253717 | actived |
| C11271 | C12127 | 0.728749325 | 0.016812618 | actived |
| C14147 | C12127 | 0.776578537 | 0.008241983 | actived |
| C17145 | C12127 | -0.696316674 | 0.02529144 | inhibted |
| C14467 | C12127 | 0.913345457 | 0.000221948 | actived |
| C08582 | C12127 | 0.946013629 | 3.48E-05 | actived |
| C08490 | C12127 | 0.648895308 | 0.042365663 | actived |
| C00109 | C07092 | -0.822323217 | 0.003497584 | inhibted |
| C00141 | C07092 | -0.716197432 | 0.019812812 | inhibted |
| C00299 | C07092 | 0.740690739 | 0.014266201 | actived |
| C00360 | C07092 | -0.678953065 | 0.030857807 | inhibted |
| C00390 | C07092 | -0.764314236 | 0.010043698 | inhibted |
| C01107 | C07092 | -0.797365755 | 0.005729604 | inhibted |
| C02934 | C07092 | -0.687974724 | 0.027871344 | inhibted |
| C02576 | C07092 | 0.825658597 | 0.003256152 | actived |
| C16794 | C07092 | -0.752226607 | 0.012074652 | inhibted |
| C13856 | C07092 | -0.756687562 | 0.011294449 | inhibted |
| C04230 | C07092 | 0.686544984 | 0.028330862 | actived |
| C04421 | C07092 | 0.706800496 | 0.022287763 | actived |
| C03577 | C07092 | 0.950584282 | 2.46E-05 | actived |
| C07339 | C07092 | 0.802092305 | 0.005245422 | actived |
| C14467 | C07092 | -0.775423691 | 0.008401036 | inhibted |
| C16856 | C07092 | -0.715478193 | 0.01999518 | inhibted |
| C00109 | C06222 | 0.656420338 | 0.039251806 | actived |
| C00141 | C06222 | 0.716267917 | 0.019795002 | actived |
| C02571 | C06222 | 0.632485152 | 0.049721569 | actived |
| C00785 | C06222 | 0.665164834 | 0.03583157 | actived |
| C00182 | C06222 | -0.694792827 | 0.025749956 | inhibted |
| C08278 | C06222 | 0.714838783 | 0.020158279 | actived |
| C00170 | C06222 | 0.893558786 | 0.000492989 | actived |
| C00016 | C06222 | 0.771101746 | 0.009015562 | actived |
| C00612 | C06222 | 0.726326513 | 0.017365298 | actived |
| C00666 | C06222 | 0.662256357 | 0.036945839 | actived |
| C02934 | C06222 | 0.673290478 | 0.032839517 | actived |
| C02378 | C06222 | 0.689664872 | 0.027334732 | actived |
| C01802 | C06222 | -0.63969442 | 0.046392984 | inhibted |
| C02165 | C06222 | 0.676431807 | 0.031729808 | actived |
| C15589 | C06222 | -0.746270423 | 0.013174259 | inhibted |
| C08842 | C06222 | 0.941206004 | 4.87E-05 | actived |
| C00521 | C06222 | 0.737636931 | 0.014889826 | actived |
| C04056 | C06222 | 0.744191196 | 0.013574069 | actived |
| C05938 | C06222 | -0.732119515 | 0.016064327 | inhibted |
| C04692 | C06222 | 0.71011222 | 0.021392539 | actived |
| C09821 | C06222 | 0.757842738 | 0.011098336 | actived |
| C06971 | C06222 | 0.699288214 | 0.024413491 | actived |
| C01425 | C06222 | 0.672358341 | 0.033173806 | actived |
| C11271 | C06222 | 0.853204597 | 0.001695115 | actived |
| C09981 | C06222 | 0.734504907 | 0.015548918 | actived |
| C14467 | C06222 | 0.703354561 | 0.023246322 | actived |
| C06559 | C06222 | -0.637883262 | 0.047214769 | inhibted |
| C08582 | C06222 | 0.730407948 | 0.016441389 | actived |
| C08490 | C06222 | 0.844334439 | 0.00211947 | actived |
| C09183 | C06222 | 0.646585263 | 0.043353819 | actived |
| C12127 | C06222 | 0.754149703 | 0.011733823 | actived |
| C00149 | C14487 | 0.712024556 | 0.020887061 | actived |
| C00082 | C14487 | 0.658899416 | 0.038260744 | actived |
| C05476 | C14487 | -0.814411315 | 0.004121322 | inhibted |
| C05607 | C14487 | -0.79823752 | 0.005638002 | inhibted |
| C08262 | C14487 | -0.913589924 | 0.000219521 | inhibted |
| C01530 | C14487 | -0.951313101 | 2.32E-05 | inhibted |
| C08278 | C14487 | -0.725062026 | 0.017658698 | inhibted |
| C00390 | C14487 | -0.637606159 | 0.04734135 | inhibted |
| C02632 | C14487 | -0.778443166 | 0.007989688 | inhibted |
| C14811 | C14487 | 0.724701445 | 0.01774299 | actived |
| C01802 | C14487 | 0.696231003 | 0.025317069 | actived |
| C15589 | C14487 | 0.788288972 | 0.006747332 | actived |
| C00823 | C14487 | -0.792613235 | 0.006247718 | inhibted |
| C05938 | C14487 | 0.666905593 | 0.035175663 | actived |
| C00157 | C14487 | 0.814243456 | 0.004135359 | actived |
| C04692 | C14487 | -0.697886224 | 0.024825061 | inhibted |
| C04421 | C14487 | 0.722650354 | 0.018227787 | actived |
| C09981 | C14487 | -0.782357614 | 0.007477902 | inhibted |
| C06559 | C14487 | 0.65559432 | 0.039585825 | actived |
| C00109 | C00081 | 0.672727273 | 0.033041223 | actived |
| C00109 | C00785 | 0.684848485 | 0.028882798 | actived |
| C00141 | C08262 | 0.684848485 | 0.028882798 | actived |
| C00082 | C00182 | 0.662823723 | 0.036726662 | actived |
| C00081 | C00182 | -0.696969697 | 0.025096676 | inhibted |
| C00081 | C01530 | 0.648484848 | 0.042540128 | actived |
| C00109 | C08278 | 0.691763757 | 0.02667824 | actived |
| C00141 | C08278 | 0.730554248 | 0.016408921 | actived |
| C01571 | C08278 | 0.821065393 | 0.00359188 | actived |
| C08262 | C08278 | 0.859855885 | 0.001420058 | actived |
| C00109 | C05463 | -0.684848485 | 0.028882798 | inhibted |
| C05476 | C05463 | -0.842424242 | 0.002220031 | inhibted |
| C01530 | C05463 | -0.672727273 | 0.033041223 | inhibted |
| C08278 | C05463 | -0.730554248 | 0.016408921 | inhibted |
| C00141 | C00360 | 0.672727273 | 0.033041223 | actived |
| C08262 | C00170 | 0.781818182 | 0.007547008 | actived |
| C00141 | C00612 | 0.745454545 | 0.013330146 | actived |
| C00785 | C00612 | 0.636363636 | 0.047911726 | actived |
| C08262 | C00612 | 0.696969697 | 0.025096676 | actived |
| C08278 | C00612 | 0.782274902 | 0.007488468 | actived |
| C05463 | C00612 | -0.721212121 | 0.018573155 | inhibted |
| C08278 | C00390 | 0.795205066 | 0.005961205 | actived |
| C00016 | C00390 | 0.636363636 | 0.047911726 | actived |
| C08278 | C01107 | 0.652973266 | 0.040658371 | actived |
| C00141 | C00666 | 0.730116312 | 0.016506245 | actived |
| C08278 | C00666 | 0.647945168 | 0.04277025 | actived |
| C08262 | C02934 | 0.709090909 | 0.021665923 | actived |
| C08278 | C02934 | 0.769344739 | 0.009274221 | actived |
| C05463 | C02934 | -0.672727273 | 0.033041223 | inhibted |
| C00785 | C02632 | 0.648484848 | 0.042540128 | actived |
| C08262 | C02632 | 0.721212121 | 0.018573155 | actived |
| C00612 | C02632 | 0.660606061 | 0.037588378 | actived |
| C00082 | C02378 | -0.712848155 | 0.020671927 | inhibted |
| C00081 | C02378 | 0.793939394 | 0.006099923 | actived |
| C08278 | C02378 | 0.665903429 | 0.03555227 | actived |
| C01107 | C02378 | 0.76969697 | 0.009221953 | actived |
| C02934 | C02378 | 0.696969697 | 0.025096676 | actived |
| C00141 | C03345 | 0.854545455 | 0.001636803 | actived |
| C08278 | C03345 | 0.717624084 | 0.019454484 | actived |
| C05463 | C03345 | -0.721212121 | 0.018573155 | inhibted |
| C00170 | C03345 | 0.696969697 | 0.025096676 | actived |
| C00016 | C03345 | 0.660606061 | 0.037588378 | actived |
| C08278 | C01802 | -0.640043102 | 0.04623588 | inhibted |
| C00141 | C02165 | 0.796356262 | 0.005836997 | actived |
| C08262 | C02165 | 0.680854209 | 0.030211171 | actived |
| C05463 | C02165 | -0.729486652 | 0.01664688 | inhibted |
| C02378 | C02165 | 0.674775153 | 0.032311809 | actived |
| C00149 | C15589 | 0.757575758 | 0.011143447 | actived |
| C08278 | C15589 | -0.743484411 | 0.013711885 | inhibted |
| C05463 | C15589 | 0.757575758 | 0.011143447 | actived |
| C00170 | C15589 | -0.684848485 | 0.028882798 | inhibted |
| C00149 | C08842 | -0.636363636 | 0.047911726 | inhibted |
| C08262 | C08842 | 0.733333333 | 0.015800596 | actived |
| C08278 | C08842 | 0.782274902 | 0.007488468 | actived |
| C05463 | C08842 | -0.660606061 | 0.037588378 | inhibted |
| C00612 | C08842 | 0.76969697 | 0.009221953 | actived |
| C00390 | C08842 | 0.672727273 | 0.033041223 | actived |
| C01107 | C08842 | 0.648484848 | 0.042540128 | actived |
| C00666 | C08842 | 0.730116312 | 0.016506245 | actived |
| C03345 | C08842 | 0.745454545 | 0.013330146 | actived |
| C02165 | C08842 | 0.826751539 | 0.003179714 | actived |
| C00109 | C00521 | 0.717845449 | 0.019399289 | actived |
| C00141 | C00521 | 0.668762 | 0.034485189 | actived |
| C00785 | C00521 | 0.631949413 | 0.049975087 | actived |
| C01571 | C00521 | 0.828283211 | 0.003074774 | actived |
| C08262 | C00521 | 0.828283211 | 0.003074774 | actived |
| C05463 | C00521 | -0.730116312 | 0.016506245 | inhibted |
| C00016 | C00521 | 0.779199761 | 0.007888893 | actived |
| C01107 | C00521 | 0.693303725 | 0.02620349 | actived |
| C02934 | C00521 | 0.766928899 | 0.009638359 | actived |
| C02632 | C00521 | 0.705574587 | 0.022625593 | actived |
| C03345 | C00521 | 0.754658037 | 0.011644873 | actived |
| C01802 | C00521 | -0.730116312 | 0.016506245 | inhibted |
| C00109 | C00823 | 0.660606061 | 0.037588378 | actived |
| C00141 | C00823 | 0.745454545 | 0.013330146 | actived |
| C00082 | C00823 | -0.644064561 | 0.044449588 | inhibted |
| C00081 | C00823 | 0.733333333 | 0.015800596 | actived |
| C01571 | C00823 | 0.696969697 | 0.025096676 | actived |
| C05463 | C00823 | -0.672727273 | 0.033041223 | inhibted |
| C00390 | C00823 | 0.636363636 | 0.047911726 | actived |
| C02378 | C00823 | 0.648484848 | 0.042540128 | actived |
| C03345 | C00823 | 0.684848485 | 0.028882798 | actived |
| C15589 | C00823 | -0.733333333 | 0.015800596 | inhibted |
| C08842 | C00823 | 0.733333333 | 0.015800596 | actived |
| C00109 | C04056 | 0.709090909 | 0.021665923 | actived |
| C01571 | C04056 | 0.696969697 | 0.025096676 | actived |
| C08262 | C04056 | 0.709090909 | 0.021665923 | actived |
| C05463 | C04056 | -0.636363636 | 0.047911726 | inhibted |
| C00360 | C04056 | 0.709090909 | 0.021665923 | actived |
| C01107 | C04056 | 0.709090909 | 0.021665923 | actived |
| C02934 | C04056 | 0.842424242 | 0.002220031 | actived |
| C02632 | C04056 | 0.660606061 | 0.037588378 | actived |
| C03345 | C04056 | 0.684848485 | 0.028882798 | actived |
| C15589 | C04056 | -0.648484848 | 0.042540128 | inhibted |
| C08842 | C04056 | 0.733333333 | 0.015800596 | actived |
| C03345 | C16794 | 0.660606061 | 0.037588378 | actived |
| C02165 | C16794 | 0.632221765 | 0.0498461 | actived |
| C00141 | C13856 | 0.636363636 | 0.047911726 | actived |
| C00109 | C14717 | 0.672727273 | 0.033041223 | actived |
| C00082 | C14717 | -0.662823723 | 0.036726662 | inhibted |
| C00785 | C14717 | 0.733333333 | 0.015800596 | actived |
| C08262 | C14717 | 0.806060606 | 0.004862061 | actived |
| C01530 | C14717 | 0.660606061 | 0.037588378 | actived |
| C05463 | C14717 | -0.721212121 | 0.018573155 | inhibted |
| C00170 | C14717 | 0.757575758 | 0.011143447 | actived |
| C02632 | C14717 | 0.721212121 | 0.018573155 | actived |
| C02378 | C14717 | 0.709090909 | 0.021665923 | actived |
| C01802 | C14717 | -0.648484848 | 0.042540128 | inhibted |
| C15589 | C14717 | -0.793939394 | 0.006099923 | inhibted |
| C08842 | C14717 | 0.745454545 | 0.013330146 | actived |
| C00823 | C14717 | 0.721212121 | 0.018573155 | actived |
| C00149 | C05938 | 0.744113425 | 0.013589186 | actived |
| C08278 | C05938 | -0.69371884 | 0.026076513 | inhibted |
| C05463 | C05938 | 0.731607317 | 0.016176528 | actived |
| C00170 | C05938 | -0.662823723 | 0.036726662 | inhibted |
| C01802 | C05938 | 0.719101209 | 0.01908823 | actived |
| C04056 | C05938 | -0.675329831 | 0.032116143 | inhibted |
| C14717 | C05938 | -0.744113425 | 0.013589186 | inhibted |
| C05463 | C00157 | 0.709090909 | 0.021665923 | actived |
| C00141 | C04692 | 0.684848485 | 0.028882798 | actived |
| C05463 | C04692 | -0.636363636 | 0.047911726 | inhibted |
| C08842 | C04692 | 0.709090909 | 0.021665923 | actived |
| C00823 | C04692 | 0.636363636 | 0.047911726 | actived |
| C00785 | C09821 | 0.709090909 | 0.021665923 | actived |
| C08262 | C09821 | 0.636363636 | 0.047911726 | actived |
| C08278 | C09821 | 0.70469392 | 0.022870448 | actived |
| C05463 | C09821 | -0.781818182 | 0.007547008 | inhibted |
| C00612 | C09821 | 0.672727273 | 0.033041223 | actived |
| C14811 | C09821 | -0.636363636 | 0.047911726 | inhibted |
| C03345 | C09821 | 0.684848485 | 0.028882798 | actived |
| C01802 | C09821 | -0.636363636 | 0.047911726 | inhibted |
| C02165 | C09821 | 0.632221765 | 0.0498461 | actived |
| C01571 | C04421 | -0.721212121 | 0.018573155 | inhibted |
| C03345 | C04421 | -0.745454545 | 0.013330146 | inhibted |
| C15589 | C04421 | 0.672727273 | 0.033041223 | actived |
| C14717 | C04421 | -0.660606061 | 0.037588378 | inhibted |
| C09821 | C04421 | -0.793939394 | 0.006099923 | inhibted |
| C00170 | C03577 | -0.684848485 | 0.028882798 | inhibted |
| C02378 | C03577 | -0.636363636 | 0.047911726 | inhibted |
| C02165 | C03577 | -0.711249486 | 0.021090926 | inhibted |
| C00109 | C20157 | 0.733333333 | 0.015800596 | actived |
| C00141 | C20157 | 0.709090909 | 0.021665923 | actived |
| C02378 | C20157 | 0.660606061 | 0.037588378 | actived |
| C08842 | C20157 | 0.733333333 | 0.015800596 | actived |
| C03577 | C20157 | -0.709090909 | 0.021665923 | inhibted |
| C03345 | C07339 | -0.636363636 | 0.047911726 | inhibted |
| C09821 | C07339 | -0.76969697 | 0.009221953 | inhibted |
| C00109 | C06971 | 0.865095798 | 0.001227392 | actived |
| C00141 | C06971 | 0.791470624 | 0.006377084 | actived |
| C00785 | C06971 | 0.828283211 | 0.003074774 | actived |
| C01571 | C06971 | 0.631949413 | 0.049975087 | actived |
| C08278 | C06971 | 0.778843182 | 0.007936284 | actived |
| C05463 | C06971 | -0.730116312 | 0.016506245 | inhibted |
| C00016 | C06971 | 0.681032862 | 0.030150884 | actived |
| C00612 | C06971 | 0.816012348 | 0.003989144 | actived |
| C01107 | C06971 | 0.717845449 | 0.019399289 | actived |
| C00666 | C06971 | 0.677018634 | 0.031525371 | actived |
| C02934 | C06971 | 0.742387174 | 0.013927768 | actived |
| C03345 | C06971 | 0.779199761 | 0.007888893 | actived |
| C02165 | C06971 | 0.78157176 | 0.007578727 | actived |
| C15589 | C06971 | -0.840554073 | 0.002321779 | inhibted |
| C13856 | C06971 | 0.631949413 | 0.049975087 | actived |
| C14717 | C06971 | 0.865095798 | 0.001227392 | actived |
| C05938 | C06971 | -0.715318828 | 0.020035745 | inhibted |
| C09821 | C06971 | 0.693303725 | 0.02620349 | actived |
| C20157 | C06971 | 0.730116312 | 0.016506245 | actived |
| C03345 | C01425 | 0.648484848 | 0.042540128 | actived |
| C08842 | C01425 | 0.696969697 | 0.025096676 | actived |
| C00521 | C01425 | 0.668762 | 0.034485189 | actived |
| C01571 | C11271 | 0.636363636 | 0.047911726 | actived |
| C02934 | C11271 | 0.696969697 | 0.025096676 | actived |
| C02632 | C11271 | 0.696969697 | 0.025096676 | actived |
| C09821 | C11271 | 0.648484848 | 0.042540128 | actived |
| C00109 | C14147 | 0.717624084 | 0.019454484 | actived |
| C00141 | C14147 | 0.678833593 | 0.030898755 | actived |
| C08262 | C14147 | 0.80813523 | 0.004669818 | actived |
| C01530 | C14147 | 0.678833593 | 0.030898755 | actived |
| C05463 | C14147 | -0.70469392 | 0.022870448 | inhibted |
| C00170 | C14147 | 0.80813523 | 0.004669818 | actived |
| C00016 | C14147 | 0.678833593 | 0.030898755 | actived |
| C02934 | C14147 | 0.795205066 | 0.005961205 | actived |
| C02632 | C14147 | 0.665903429 | 0.03555227 | actived |
| C02378 | C14147 | 0.717624084 | 0.019454484 | actived |
| C15589 | C14147 | -0.691763757 | 0.02667824 | inhibted |
| C08842 | C14147 | 0.730554248 | 0.016408921 | actived |
| C00823 | C14147 | 0.80813523 | 0.004669818 | actived |
| C04421 | C14147 | -0.756414575 | 0.011341147 | inhibted |
| C03577 | C14147 | -0.678833593 | 0.030898755 | inhibted |
| C06971 | C14147 | 0.805022784 | 0.004960321 | actived |
| C01425 | C14147 | 0.640043102 | 0.04623588 | actived |
| C00081 | C17145 | -0.636363636 | 0.047911726 | inhibted |
| C00785 | C17145 | -0.745454545 | 0.013330146 | inhibted |
| C05607 | C17145 | -0.660606061 | 0.037588378 | inhibted |
| C08262 | C17145 | -0.696969697 | 0.025096676 | inhibted |
| C08278 | C17145 | -0.730554248 | 0.016408921 | inhibted |
| C01107 | C17145 | -0.733333333 | 0.015800596 | inhibted |
| C00666 | C17145 | -0.644220275 | 0.044381366 | inhibted |
| C02632 | C17145 | -0.672727273 | 0.033041223 | inhibted |
| C03345 | C17145 | -0.903030303 | 0.000343612 | inhibted |
| C08842 | C17145 | -0.648484848 | 0.042540128 | inhibted |
| C00521 | C17145 | -0.766928899 | 0.009638359 | inhibted |
| C00823 | C17145 | -0.648484848 | 0.042540128 | inhibted |
| C02576 | C17145 | 0.660606061 | 0.037588378 | actived |
| C13856 | C17145 | -0.721212121 | 0.018573155 | inhibted |
| C14717 | C17145 | -0.854545455 | 0.001636803 | inhibted |
| C20157 | C17145 | -0.684848485 | 0.028882798 | inhibted |
| C07339 | C17145 | 0.660606061 | 0.037588378 | actived |
| C06971 | C17145 | -0.791470624 | 0.006377084 | inhibted |
| C14147 | C17145 | -0.833995557 | 0.002705284 | inhibted |
| C00149 | C09981 | -0.709090909 | 0.021665923 | inhibted |
| C08262 | C09981 | 0.781818182 | 0.007547008 | actived |
| C00182 | C09981 | -0.696969697 | 0.025096676 | inhibted |
| C00170 | C09981 | 0.648484848 | 0.042540128 | actived |
| C02378 | C09981 | 0.733333333 | 0.015800596 | actived |
| C08842 | C09981 | 0.672727273 | 0.033041223 | actived |
| C04056 | C09981 | 0.636363636 | 0.047911726 | actived |
| C14717 | C09981 | 0.757575758 | 0.011143447 | actived |
| C14147 | C09981 | 0.795205066 | 0.005961205 | actived |
| C08262 | C14467 | 0.631949413 | 0.049975087 | actived |
| C08278 | C14467 | 0.726483976 | 0.017329 | actived |
| C00823 | C14467 | 0.644220275 | 0.044381366 | actived |
| C09821 | C14467 | 0.656491137 | 0.039223265 | actived |
| C00149 | C06559 | 0.684848485 | 0.028882798 | actived |
| C00081 | C06559 | -0.721212121 | 0.018573155 | inhibted |
| C01571 | C06559 | -0.672727273 | 0.033041223 | inhibted |
| C08262 | C06559 | -0.660606061 | 0.037588378 | inhibted |
| C02378 | C06559 | -0.76969697 | 0.009221953 | inhibted |
| C00823 | C06559 | -0.757575758 | 0.011143447 | inhibted |
| C05476 | C16856 | 0.696969697 | 0.025096676 | actived |
| C05463 | C16856 | -0.636363636 | 0.047911726 | inhibted |
| C00823 | C16856 | 0.709090909 | 0.021665923 | actived |
| C17145 | C16856 | -0.721212121 | 0.018573155 | inhibted |
| C00141 | C08582 | 0.866666667 | 0.001173538 | actived |
| C00149 | C08582 | -0.733333333 | 0.015800596 | inhibted |
| C01571 | C08582 | 0.660606061 | 0.037588378 | actived |
| C08278 | C08582 | 0.756414575 | 0.011341147 | actived |
| C05463 | C08582 | -0.636363636 | 0.047911726 | inhibted |
| C09821 | C08582 | 0.684848485 | 0.028882798 | actived |
| C04421 | C08582 | -0.636363636 | 0.047911726 | inhibted |
| C17145 | C08582 | -0.781818182 | 0.007547008 | inhibted |
| C01571 | C08490 | 0.76969697 | 0.009221953 | actived |
| C00612 | C08490 | 0.709090909 | 0.021665923 | actived |
| C02934 | C08490 | 0.660606061 | 0.037588378 | actived |
| C02632 | C08490 | 0.696969697 | 0.025096676 | actived |
| C03345 | C08490 | 0.696969697 | 0.025096676 | actived |
| C02165 | C08490 | 0.699091375 | 0.024470991 | actived |
| C15589 | C08490 | -0.757575758 | 0.011143447 | inhibted |
| C02576 | C08490 | -0.636363636 | 0.047911726 | inhibted |
| C14717 | C08490 | 0.709090909 | 0.021665923 | actived |
| C20157 | C08490 | 0.684848485 | 0.028882798 | actived |
| C07339 | C08490 | -0.660606061 | 0.037588378 | inhibted |
| C14147 | C08490 | 0.795205066 | 0.005961205 | actived |
| C08582 | C08490 | 0.696969697 | 0.025096676 | actived |
| C00299 | C09183 | -0.741644763 | 0.014075178 | inhibted |
| C08262 | C09183 | 0.644379876 | 0.044311514 | actived |
| C00612 | C09183 | 0.674775153 | 0.032311809 | actived |
| C00390 | C09183 | 0.656537987 | 0.039204386 | actived |
| C01107 | C09183 | 0.656537987 | 0.039204386 | actived |
| C00666 | C09183 | 0.63387316 | 0.049068733 | actived |
| C02934 | C09183 | 0.784198151 | 0.007245486 | actived |
| C03345 | C09183 | 0.668696098 | 0.034509542 | actived |
| C02165 | C09183 | 0.698170732 | 0.024741158 | actived |
| C08842 | C09183 | 0.741644763 | 0.014075178 | actived |
| C00823 | C09183 | 0.686933264 | 0.028205561 | actived |
| C04056 | C09183 | 0.644379876 | 0.044311514 | actived |
| C16794 | C09183 | 0.814593429 | 0.004106132 | actived |
| C04692 | C09183 | 0.711249486 | 0.021090926 | actived |
| C04421 | C09183 | -0.77204004 | 0.008879537 | inhibted |
| C03577 | C09183 | -0.717328542 | 0.019528343 | inhibted |
| C20157 | C09183 | 0.699091375 | 0.024470991 | actived |
| C06971 | C09183 | 0.640027268 | 0.046243006 | actived |
| C14147 | C09183 | 0.654961012 | 0.039843211 | actived |
| C14467 | C09183 | 0.78157176 | 0.007578727 | actived |
| C08582 | C09183 | 0.784198151 | 0.007245486 | actived |
| C08278 | C12127 | 0.70469392 | 0.022870448 | actived |
| C09821 | C12127 | 0.636363636 | 0.047911726 | actived |
| C09183 | C12127 | 0.765960985 | 0.00978704 | actived |
| C00081 | C07092 | -0.636363636 | 0.047911726 | inhibted |
| C00612 | C07092 | -0.648484848 | 0.042540128 | inhibted |
| C03345 | C07092 | -0.806060606 | 0.004862061 | inhibted |
| C17145 | C07092 | 0.781818182 | 0.007547008 | actived |
| C08582 | C07092 | -0.709090909 | 0.021665923 | inhibted |
| C09183 | C07092 | -0.69301232 | 0.026292878 | inhibted |
| C12127 | C07092 | -0.660606061 | 0.037588378 | inhibted |
| C00149 | C06222 | -0.696969697 | 0.025096676 | inhibted |
| C08262 | C06222 | 0.672727273 | 0.033041223 | actived |
| C00390 | C06222 | 0.660606061 | 0.037588378 | actived |
| C02632 | C06222 | 0.660606061 | 0.037588378 | actived |
| C03345 | C06222 | 0.660606061 | 0.037588378 | actived |
| C14717 | C06222 | 0.76969697 | 0.009221953 | actived |
| C20157 | C06222 | 0.733333333 | 0.015800596 | actived |
| C14147 | C06222 | 0.717624084 | 0.019454484 | actived |
| C00109 | C14487 | -0.660606061 | 0.037588378 | inhibted |
| C00141 | C14487 | -0.672727273 | 0.033041223 | inhibted |
| C00081 | C14487 | -0.745454545 | 0.013330146 | inhibted |
| C01571 | C14487 | -0.636363636 | 0.047911726 | inhibted |
| C05463 | C14487 | 0.793939394 | 0.006099923 | actived |
| C00170 | C14487 | -0.696969697 | 0.025096676 | inhibted |
| C00612 | C14487 | -0.660606061 | 0.037588378 | inhibted |
| C02934 | C14487 | -0.684848485 | 0.028882798 | inhibted |
| C03345 | C14487 | -0.636363636 | 0.047911726 | inhibted |
| C02165 | C14487 | -0.711249486 | 0.021090926 | inhibted |
| C08842 | C14487 | -0.733333333 | 0.015800596 | inhibted |
| C00521 | C14487 | -0.803741486 | 0.005083578 | inhibted |
| C14717 | C14487 | -0.818181818 | 0.00381492 | inhibted |
| C06971 | C14487 | -0.779199761 | 0.007888893 | inhibted |
| C14147 | C14487 | -0.821065393 | 0.00359188 | inhibted |
| C17145 | C14487 | 0.684848485 | 0.028882798 | actived |
| C14467 | C14487 | -0.631949413 | 0.049975087 | inhibted |
| C08490 | C14487 | -0.721212121 | 0.018573155 | inhibted |
| C06222 | C14487 | -0.648484848 | 0.042540128 | inhibted |

Table S16 Positive co-expressed links in grafts for liver transplantation

| row | column | cor | p | interaction |
| --- | --- | --- | --- | --- |
| C00944 | C04242 | 0.45085474 | 3.05E-05 | activated |
| C00944 | C19848 | 0.256486528 | 0.022508345 | activated |
| C04242 | C19848 | 0.232232824 | 0.039448484 | activated |
| C14827 | C01041 | 0.313446008 | 0.004910923 | activated |
| C00944 | C01041 | 0.258961719 | 0.021195921 | activated |
| C19848 | C01041 | 0.354692927 | 0.001339912 | activated |
| C00944 | C03626 | 0.339245286 | 0.002224752 | activated |
| C04242 | C03626 | 0.454987556 | 2.52E-05 | activated |
| C19848 | C03626 | 0.224721171 | 0.046472758 | activated |
| C04242 | C06916 | 0.38645188 | 0.000435091 | activated |
| C19848 | C06916 | 0.33843387 | 0.002283212 | activated |
| C01041 | C06916 | 0.285906433 | 0.010641853 | activated |
| C03626 | C06916 | 0.331980112 | 0.002799541 | activated |
| C00944 | C00300 | 0.265262902 | 0.018146193 | activated |
| C04242 | C00300 | 0.499577042 | 2.77E-06 | activated |
| C19848 | C00300 | 0.266548909 | 0.017572376 | activated |
| C03626 | C00300 | 0.241533781 | 0.031997625 | activated |
| C06916 | C00300 | 0.388159616 | 0.000408215 | activated |
| C14827 | C02824 | -0.58947535 | 1.09E-08 | inhibited |
| C01041 | C02824 | -0.383543303 | 0.000484616 | inhibited |
| C14827 | C15643 | 0.385483967 | 0.000451032 | activated |
| C01041 | C15643 | 0.36590045 | 0.000912666 | activated |
| C06916 | C15643 | 0.232366332 | 0.039332116 | activated |
| C02824 | C15643 | -0.459318502 | 2.06E-05 | inhibited |
| C00944 | C05570 | 0.421348588 | 0.000110179 | activated |
| C04242 | C05570 | 0.378653716 | 0.000579614 | activated |
| C19848 | C05570 | 0.314838531 | 0.00471347 | activated |
| C01041 | C05570 | 0.251658451 | 0.025268342 | activated |
| C03626 | C05570 | 0.222276325 | 0.04897031 | activated |
| C00300 | C05570 | 0.429552093 | 7.80E-05 | activated |
| C19848 | C00668 | 0.329938678 | 0.002983368 | activated |
| C01041 | C00668 | 0.272696648 | 0.015039732 | activated |
| C06916 | C00668 | 0.317047512 | 0.004414677 | activated |
| C14827 | C10689 | 0.509070992 | 1.66E-06 | activated |
| C00944 | C10689 | 0.408067382 | 0.000189169 | activated |
| C04242 | C10689 | 0.265636126 | 0.017978027 | activated |
| C19848 | C10689 | 0.446775674 | 3.67E-05 | activated |
| C01041 | C10689 | 0.592272955 | 8.95E-09 | activated |
| C03626 | C10689 | 0.268089304 | 0.016905638 | activated |
| C02824 | C10689 | -0.406241632 | 0.000203406 | inhibited |
| C15643 | C10689 | 0.323866053 | 0.003595681 | activated |
| C05570 | C10689 | 0.443827747 | 4.19E-05 | activated |
| C00668 | C10689 | 0.232264726 | 0.039420652 | activated |
| C06916 | C00149 | -0.484317911 | 6.11E-06 | inhibited |
| C00300 | C00149 | -0.294341864 | 0.008461605 | inhibited |
| C10689 | C00149 | 0.247637993 | 0.027780905 | activated |
| C14827 | C04025 | 0.253071956 | 0.02443206 | activated |
| C00944 | C04025 | 0.313809264 | 0.004858721 | activated |
| C04242 | C04025 | 0.232683542 | 0.039056774 | activated |
| C05570 | C04025 | 0.284559674 | 0.011031856 | activated |
| C00149 | C04025 | -0.318380688 | 0.004242602 | inhibited |
| C00944 | C02728 | 0.473417722 | 1.05E-05 | activated |
| C04242 | C02728 | 0.405929929 | 0.000205933 | activated |
| C19848 | C02728 | 0.289504864 | 0.009658183 | activated |
| C03626 | C02728 | 0.382629339 | 0.000501212 | activated |
| C00300 | C02728 | 0.299123661 | 0.00740848 | activated |
| C05570 | C02728 | 0.264191821 | 0.018636315 | activated |
| C14827 | C16576 | 0.311085415 | 0.00526244 | activated |
| C00944 | C16576 | 0.302855474 | 0.006668482 | activated |
| C04242 | C16576 | 0.221866537 | 0.049399547 | activated |
| C19848 | C16576 | 0.242483538 | 0.031308219 | activated |
| C01041 | C16576 | 0.558725792 | 8.74E-08 | activated |
| C06916 | C16576 | 0.240805769 | 0.032534669 | activated |
| C15643 | C16576 | 0.276945619 | 0.013479351 | activated |
| C05570 | C16576 | 0.270211057 | 0.016022975 | activated |
| C10689 | C16576 | 0.568333414 | 4.67E-08 | activated |
| C14827 | C11806 | 0.297964906 | 0.007652496 | activated |
| C06916 | C11806 | -0.253264935 | 0.024319731 | inhibited |
| C02824 | C11806 | -0.230457802 | 0.041023051 | inhibited |
| C10689 | C11806 | 0.276223518 | 0.013734197 | activated |
| C00149 | C11806 | 0.272873765 | 0.014971701 | activated |
| C16576 | C11806 | 0.222099722 | 0.049154917 | activated |
| C14827 | C00864 | 0.330771244 | 0.002907132 | activated |
| C00944 | C00864 | 0.380598832 | 0.000539955 | activated |
| C19848 | C00864 | 0.298170032 | 0.007608792 | activated |
| C01041 | C00864 | 0.384529244 | 0.000467279 | activated |
| C06916 | C00864 | 0.282000256 | 0.011807441 | activated |
| C15643 | C00864 | 0.259086536 | 0.021131508 | activated |
| C05570 | C00864 | 0.358787731 | 0.001166382 | activated |
| C10689 | C00864 | 0.515762312 | 1.15E-06 | activated |
| C16576 | C00864 | 0.389639475 | 0.000386165 | activated |
| C14827 | C00157 | -0.504558235 | 2.12E-06 | inhibited |
| C19848 | C00157 | -0.286840875 | 0.010378365 | inhibited |
| C01041 | C00157 | -0.37802555 | 0.000592979 | inhibited |
| C02824 | C00157 | 0.478070095 | 8.36E-06 | activated |
| C15643 | C00157 | -0.387405761 | 0.000419886 | inhibited |
| C05570 | C00157 | -0.261978225 | 0.019685324 | inhibited |
| C00668 | C00157 | -0.258299559 | 0.021540451 | inhibited |
| C10689 | C00157 | -0.435504975 | 6.04E-05 | inhibited |
| C04025 | C00157 | -0.371945542 | 0.000737648 | inhibited |
| C16576 | C00157 | -0.291075069 | 0.009254479 | inhibited |
| C00864 | C00157 | -0.229151833 | 0.042214575 | inhibited |
| C00944 | C05983 | 0.239635439 | 0.033413859 | activated |
| C04025 | C05983 | 0.248932504 | 0.026949813 | activated |
| C00864 | C05983 | 0.284505245 | 0.011047876 | activated |
| C00157 | C05983 | -0.240069767 | 0.03308528 | inhibited |
| C00944 | C00449 | 0.274044636 | 0.01452862 | activated |
| C04242 | C00449 | 0.479903597 | 7.63E-06 | activated |
| C19848 | C00449 | 0.245778764 | 0.029012386 | activated |
| C01041 | C00449 | 0.343057474 | 0.0019677 | activated |
| C03626 | C00449 | 0.344618326 | 0.001870404 | activated |
| C06916 | C00449 | 0.441962656 | 4.55E-05 | activated |
| C00300 | C00449 | 0.393514659 | 0.000333491 | activated |
| C15643 | C00449 | 0.358341477 | 0.001184251 | activated |
| C05570 | C00449 | 0.473558966 | 1.04E-05 | activated |
| C00668 | C00449 | 0.230250389 | 0.041210401 | activated |
| C10689 | C00449 | 0.502687076 | 2.35E-06 | activated |
| C00149 | C00449 | -0.236509846 | 0.03586007 | inhibited |
| C02728 | C00449 | 0.243236829 | 0.030770347 | activated |
| C16576 | C00449 | 0.466400901 | 1.48E-05 | activated |
| C00864 | C00449 | 0.368963356 | 0.000819758 | activated |
| C00157 | C00449 | -0.266335176 | 0.017666649 | inhibited |
| C14827 | C01829 | 0.469413262 | 1.28E-05 | activated |
| C19848 | C01829 | 0.33604438 | 0.002463462 | activated |
| C01041 | C01829 | 0.599396309 | 5.33E-09 | activated |
| C06916 | C01829 | 0.255524796 | 0.023036646 | activated |
| C02824 | C01829 | -0.551943019 | 1.34E-07 | inhibited |
| C15643 | C01829 | 0.459430505 | 2.05E-05 | activated |
| C05570 | C01829 | 0.261451883 | 0.019942065 | activated |
| C10689 | C01829 | 0.511527309 | 1.45E-06 | activated |
| C16576 | C01829 | 0.438976713 | 5.19E-05 | activated |
| C11806 | C01829 | 0.223088488 | 0.048128669 | activated |
| C00864 | C01829 | 0.343565425 | 0.001935548 | activated |
| C00157 | C01829 | -0.433323512 | 6.64E-05 | inhibited |
| C00449 | C01829 | 0.34042052 | 0.002142464 | activated |
| C14827 | C04230 | 0.538698792 | 3.04E-07 | activated |
| C19848 | C04230 | 0.268469931 | 0.016744283 | activated |
| C01041 | C04230 | 0.451864594 | 2.91E-05 | activated |
| C06916 | C04230 | 0.267747452 | 0.017051694 | activated |
| C02824 | C04230 | -0.602669153 | 4.19E-09 | inhibited |
| C15643 | C04230 | 0.63128572 | 4.46E-10 | activated |
| C05570 | C04230 | 0.259287607 | 0.021028093 | activated |
| C10689 | C04230 | 0.504124306 | 2.17E-06 | activated |
| C04025 | C04230 | 0.276898278 | 0.013495933 | activated |
| C16576 | C04230 | 0.341351584 | 0.002079226 | activated |
| C00864 | C04230 | 0.437447144 | 5.55E-05 | activated |
| C00157 | C04230 | -0.491507037 | 4.23E-06 | inhibited |
| C00449 | C04230 | 0.354920927 | 0.001329668 | activated |
| C01829 | C04230 | 0.572598533 | 3.51E-08 | activated |

Table S17 Co-expressed links centered by C00157 and C04230 in hepatocytes categorized by PKLR perturbation

| row | column | cor | p | interaction |
| --- | --- | --- | --- | --- |
| C00164 | C00157 | 0.766714994 | 0.009671079 | actived |
| C00058 | C00157 | 0.706020176 | 0.022502395 | actived |
| C00137 | C00157 | 0.7598736 | 0.01075938 | actived |
| C19654 | C00157 | 0.644927112 | 0.044072567 | actived |
| C17714 | C00157 | 0.677987012 | 0.031189973 | actived |
| C08281 | C00157 | -0.696238841 | 0.025314723 | inhibted |
| C06524 | C00157 | 0.756769412 | 0.011280474 | actived |
| C00184 | C00157 | 0.697214998 | 0.025023781 | actived |
| C05552 | C00157 | -0.750221612 | 0.012437326 | inhibted |
| C00521 | C00157 | -0.646262449 | 0.043493127 | inhibted |
| C01290 | C00157 | -0.780864606 | 0.007670274 | inhibted |
| C05579 | C00157 | 0.721119866 | 0.018595462 | actived |
| C00164 | C04230 | 0.644652038 | 0.044192568 | actived |
| C00058 | C04230 | 0.870299897 | 0.001055583 | actived |
| C00137 | C04230 | 0.661232938 | 0.037343423 | actived |
| C01879 | C04230 | 0.956198236 | 1.53E-05 | actived |
| C01595 | C04230 | 0.929462178 | 9.94E-05 | actived |
| C01530 | C04230 | 0.712217108 | 0.020836626 | actived |
| C04299 | C04230 | 0.694486352 | 0.025842855 | actived |
| C06524 | C04230 | 0.833426876 | 0.002740554 | actived |
| C00612 | C04230 | -0.638881179 | 0.046760787 | inhibted |
| C01107 | C04230 | -0.770348873 | 0.009125765 | inhibted |
| C06425 | C04230 | -0.768514634 | 0.009398228 | inhibted |
| C05552 | C04230 | -0.782366053 | 0.007476824 | inhibted |
| C08842 | C04230 | 0.75684869 | 0.01126695 | actived |
| C14833 | C04230 | 0.871522073 | 0.00101792 | actived |
| C06575 | C04230 | -0.769019283 | 0.009322701 | inhibted |
| C00350 | C04230 | -0.828352535 | 0.003070084 | inhibted |
| C04230 | C02277 | 0.944836538 | 3.79E-05 | actived |
| C00157 | C04692 | -0.67856909 | 0.030989542 | inhibted |
| C04230 | C04692 | -0.767946997 | 0.009483696 | inhibted |
| C00157 | C07598 | 0.802391765 | 0.005215765 | actived |
| C04230 | C07934 | 0.790031784 | 0.006542683 | actived |
| C04230 | C07468 | 0.992523668 | 1.35E-08 | actived |
| C00157 | C08052 | -0.693142356 | 0.026252964 | inhibted |
| C04230 | C08052 | -0.731732143 | 0.016149134 | inhibted |
| C00157 | C07363 | 0.640740736 | 0.045922618 | actived |
| C04230 | C07363 | 0.650767482 | 0.041575993 | actived |
| C04230 | C12811 | 0.887860582 | 0.000603029 | actived |
| C04230 | C07913 | 0.639963725 | 0.046271613 | actived |
| C04230 | C01948 | 0.674853805 | 0.032284015 | actived |
| C04230 | C08497 | 0.874836862 | 0.000920696 | actived |
| C00157 | C11882 | 0.822591132 | 0.003477731 | actived |
| C04230 | C11882 | 0.674585421 | 0.032378923 | actived |
| C04230 | C10080 | 0.813257296 | 0.00421852 | actived |
| C00157 | C07092 | -0.664126511 | 0.036226717 | inhibted |
| C04230 | C07092 | -0.648915717 | 0.042357001 | inhibted |
| C04230 | C13482 | -0.865988101 | 0.001196586 | inhibted |
| C04230 | C06205 | 0.806125787 | 0.004855937 | actived |
| C04230 | C14487 | -0.772160799 | 0.008862136 | inhibted |
| C04230 | C11129 | -0.674041979 | 0.03257168 | inhibted |
| C00141 | C00157 | -0.636363636 | 0.047911726 | inhibted |
| C00212 | C00157 | 0.696969697 | 0.025096676 | actived |
| C01595 | C00157 | 0.636363636 | 0.047911726 | actived |
| C08278 | C00157 | -0.696969697 | 0.025096676 | inhibted |
| C00612 | C00157 | -0.660606061 | 0.037588378 | inhibted |
| C06575 | C00157 | -0.648484848 | 0.042540128 | inhibted |
| C00141 | C04230 | -0.719101209 | 0.01908823 | inhibted |
| C17714 | C04230 | 0.694088993 | 0.025963645 | actived |
| C08278 | C04230 | -0.650317615 | 0.041764833 | inhibted |
| C08281 | C04230 | -0.639355767 | 0.04654591 | inhibted |
| C12621 | C04230 | -0.6397531 | 0.04636652 | inhibted |
| C00184 | C04230 | 0.706595101 | 0.022344121 | actived |
| C00521 | C04230 | -0.694088993 | 0.025963645 | inhibted |
| C06834 | C04230 | -0.725354263 | 0.017590587 | inhibted |
| C00157 | C04230 | 0.731607317 | 0.016176528 | actived |
| C00157 | C04317 | 0.696969697 | 0.025096676 | actived |
| C00157 | C02277 | 0.648484848 | 0.042540128 | actived |
| C04230 | C03577 | -0.694088993 | 0.025963645 | inhibted |
| C00157 | C07310 | -0.709090909 | 0.021665923 | inhibted |
| C04230 | C07598 | 0.644064561 | 0.044449588 | actived |
| C00157 | C07468 | 0.719101209 | 0.01908823 | actived |
| C04230 | C07372 | 0.637811507 | 0.047247525 | actived |
| C00157 | C14467 | -0.648484848 | 0.042540128 | inhibted |
| C00157 | C10478 | 0.660606061 | 0.037588378 | actived |
| C00157 | C13482 | -0.648484848 | 0.042540128 | inhibted |
| C00149 | C00157 | 0.739598945 | 0.014487022 | actived |
| C00081 | C00157 | -0.751135658 | 0.012271055 | inhibted |
| C02571 | C00157 | -0.735480015 | 0.015341584 | inhibted |
| C00785 | C00157 | -0.726124859 | 0.01741186 | inhibted |
| C01571 | C00157 | -0.663410734 | 0.036500823 | inhibted |
| C08262 | C00157 | -0.79823192 | 0.005638587 | inhibted |
| C01530 | C00157 | -0.712780753 | 0.020689475 | inhibted |
| C08278 | C00157 | -0.705800561 | 0.022563057 | inhibted |
| C00390 | C00157 | -0.675668255 | 0.031997159 | inhibted |
| C02632 | C00157 | -0.681394963 | 0.030028945 | inhibted |
| C01802 | C00157 | 0.678483592 | 0.031018927 | actived |
| C02165 | C00157 | -0.655788122 | 0.039507286 | inhibted |
| C15589 | C00157 | 0.832196964 | 0.002817963 | actived |
| C00521 | C00157 | -0.799676716 | 0.005489066 | inhibted |
| C00823 | C00157 | -0.718927932 | 0.019130945 | inhibted |
| C04056 | C00157 | -0.660145625 | 0.037768983 | inhibted |
| C14717 | C00157 | -0.737416757 | 0.01493551 | inhibted |
| C05938 | C00157 | 0.715211621 | 0.020063065 | actived |
| C00109 | C04230 | -0.712635374 | 0.02072736 | inhibted |
| C00141 | C04230 | -0.709707358 | 0.021500626 | inhibted |
| C00182 | C04230 | 0.66060542 | 0.037588629 | actived |
| C00360 | C04230 | -0.723980151 | 0.017912442 | inhibted |
| C00016 | C04230 | -0.711929085 | 0.020912099 | inhibted |
| C02934 | C04230 | -0.639314598 | 0.046564524 | inhibted |
| C02576 | C04230 | 0.696613145 | 0.02520289 | actived |
| C00157 | C04692 | -0.724520266 | 0.017785449 | inhibted |
| C04230 | C09821 | -0.81676866 | 0.003927773 | inhibted |
| C00157 | C04421 | 0.684280918 | 0.029069075 | actived |
| C04230 | C03577 | 0.757050182 | 0.011232628 | actived |
| C00157 | C20157 | -0.646137835 | 0.043546983 | inhibted |
| C04230 | C07339 | 0.636747374 | 0.047735086 | actived |
| C00157 | C06971 | -0.67728799 | 0.031431834 | inhibted |
| C04230 | C01425 | -0.729875395 | 0.016559956 | inhibted |
| C00157 | C11271 | -0.636391109 | 0.047899066 | inhibted |
| C00157 | C14147 | -0.717572429 | 0.019467379 | inhibted |
| C04230 | C17145 | 0.6467614 | 0.043277935 | actived |
| C00157 | C09981 | -0.740385422 | 0.014327715 | inhibted |
| C04230 | C14467 | -0.646836222 | 0.043245727 | inhibted |
| C00157 | C06559 | 0.838344223 | 0.002446296 | actived |
| C04230 | C08582 | -0.635045733 | 0.048521695 | inhibted |
| C00157 | C08490 | -0.66455354 | 0.036063851 | inhibted |
| C04230 | C08490 | -0.651939854 | 0.041086564 | inhibted |
| C04230 | C09183 | -0.803121617 | 0.005143985 | inhibted |
| C04230 | C12127 | -0.696217018 | 0.025321254 | inhibted |
| C04230 | C07092 | 0.686544984 | 0.028330862 | actived |
| C00157 | C14487 | 0.814243456 | 0.004135359 | actived |
| C05463 | C00157 | 0.709090909 | 0.021665923 | actived |

Table S18 Co-expressed links centered by C00157 and C04230 in grafts categorized by PKLR expression

| row | column | cor | p | interaction |
| --- | --- | --- | --- | --- |
| C14827 | C00157 | -0.504558235 | 2.12E-06 | inhibited |
| C19848 | C00157 | -0.286840875 | 0.010378365 | inhibited |
| C01041 | C00157 | -0.37802555 | 0.000592979 | inhibited |
| C02824 | C00157 | 0.478070095 | 8.36E-06 | activated |
| C15643 | C00157 | -0.387405761 | 0.000419886 | inhibited |
| C05570 | C00157 | -0.261978225 | 0.019685324 | inhibited |
| C00668 | C00157 | -0.258299559 | 0.021540451 | inhibited |
| C10689 | C00157 | -0.435504975 | 6.04E-05 | inhibited |
| C04025 | C00157 | -0.371945542 | 0.000737648 | inhibited |
| C16576 | C00157 | -0.291075069 | 0.009254479 | inhibited |
| C00864 | C00157 | -0.229151833 | 0.042214575 | inhibited |
| C00157 | C05983 | -0.240069767 | 0.03308528 | inhibited |
| C00157 | C00449 | -0.266335176 | 0.017666649 | inhibited |
| C00157 | C01829 | -0.433323512 | 6.64E-05 | inhibited |
| C14827 | C04230 | 0.538698792 | 3.04E-07 | activated |
| C19848 | C04230 | 0.268469931 | 0.016744283 | activated |
| C01041 | C04230 | 0.451864594 | 2.91E-05 | activated |
| C06916 | C04230 | 0.267747452 | 0.017051694 | activated |
| C02824 | C04230 | -0.602669153 | 4.19E-09 | inhibited |
| C15643 | C04230 | 0.63128572 | 4.46E-10 | activated |
| C05570 | C04230 | 0.259287607 | 0.021028093 | activated |
| C10689 | C04230 | 0.504124306 | 2.17E-06 | activated |
| C04025 | C04230 | 0.276898278 | 0.013495933 | activated |
| C16576 | C04230 | 0.341351584 | 0.002079226 | activated |
| C00864 | C04230 | 0.437447144 | 5.55E-05 | activated |
| C00157 | C04230 | -0.491507037 | 4.23E-06 | inhibited |
| C00449 | C04230 | 0.354920927 | 0.001329668 | activated |
| C01829 | C04230 | 0.572598533 | 3.51E-08 | activated |


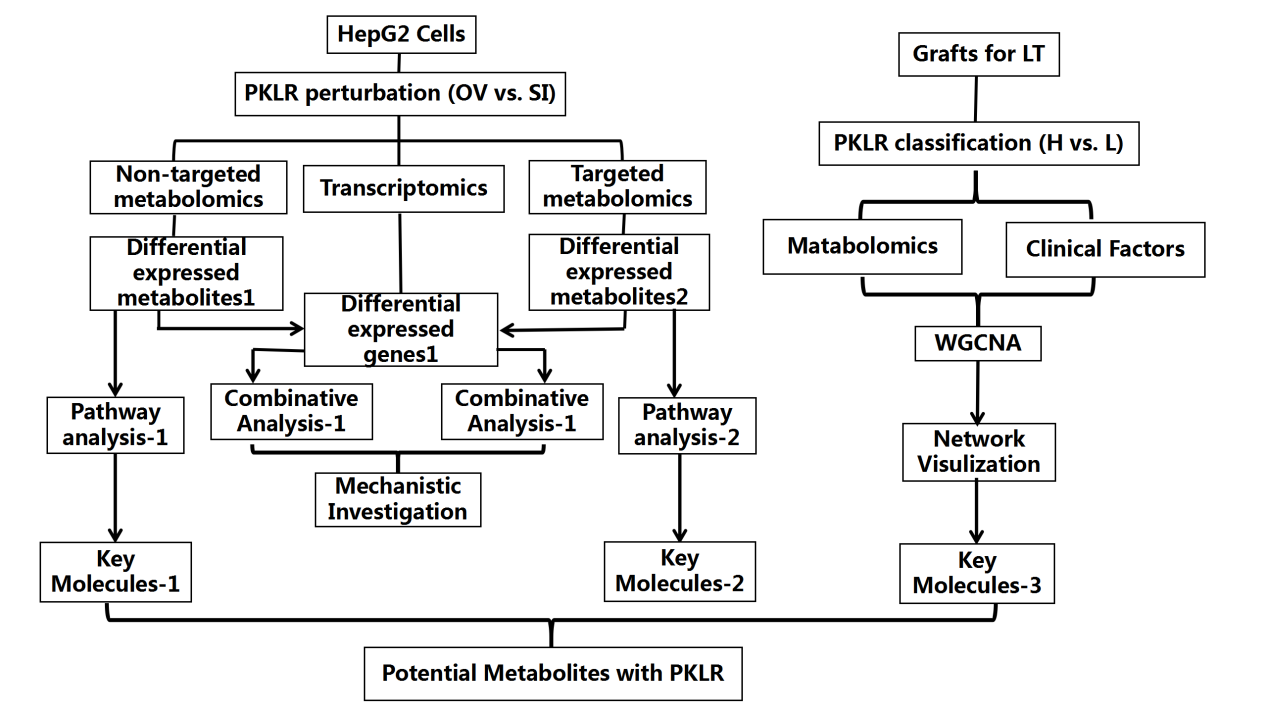


Figure S1 Flow diagram of study design

Abbreviations: OV, overexpression; SI, silence.


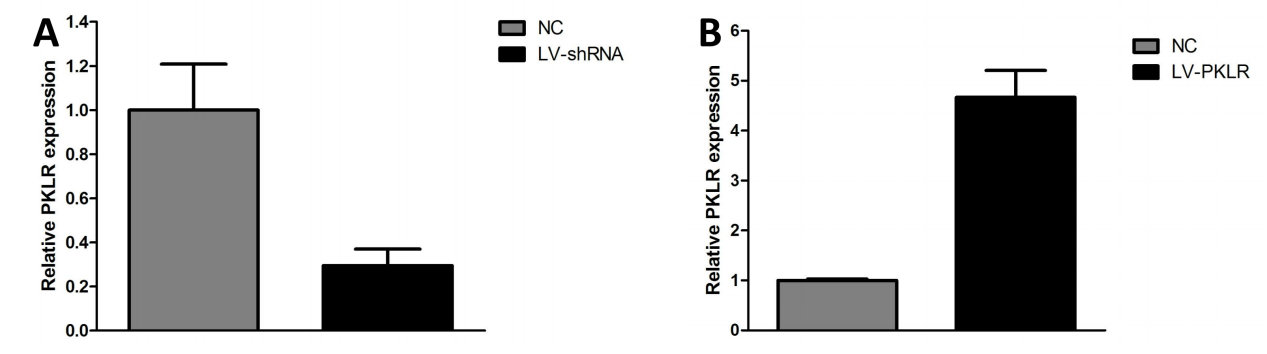


Figure S2 Construction of hepatocytes with PKLR perturbation

1. Impact of LV packed shRNA on down-regulation of PKLR;
2. Impact of LV packed plasmid on overexpression of PKLR.

Abbreviations: LV, lenti-virus.


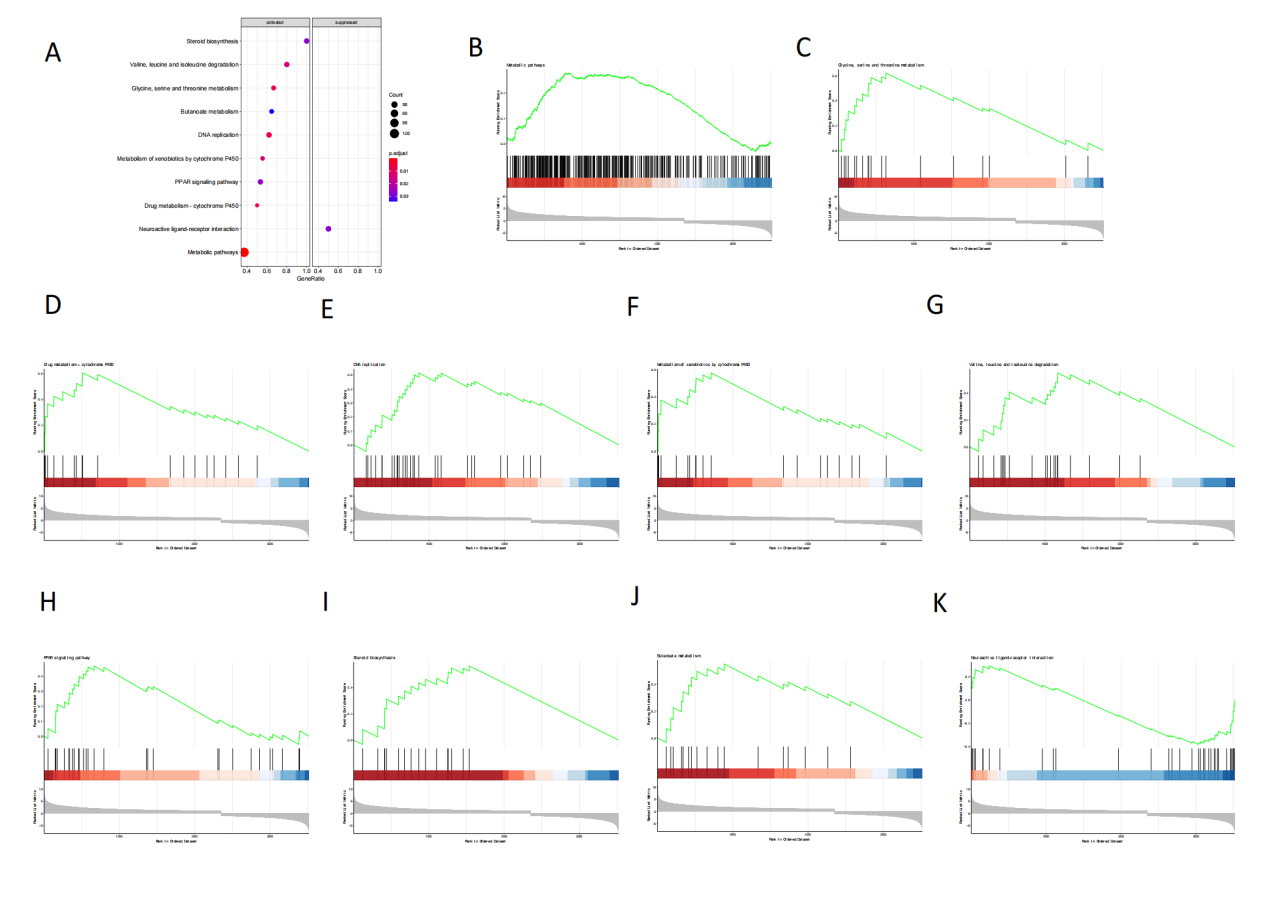


Figure S3 KEGG pathway enrichment on differential genes from HepG2 cells with overexpressed PKLR by GSEA

1. Bubble plots showed nine positively and one negatively correlated KEGG pathways significantly associated with PKLR overexpression;

B-K. Details on individualized results in each significant KEGG pathways by GSEA.

Abbreviations: GSEA, Gene Set Enrichment Analysis.


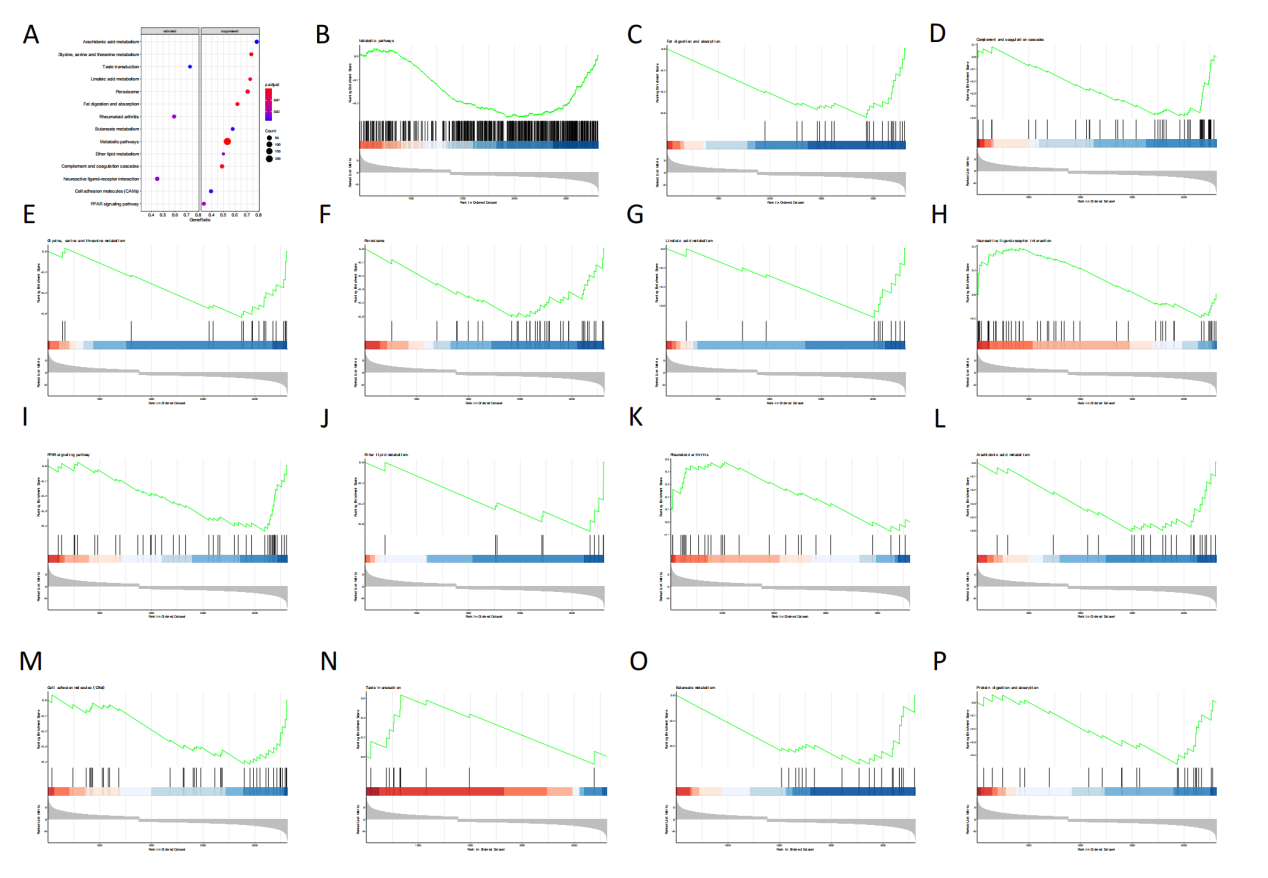


Figure S4 KEGG pathway enrichment on differential genes from HepG2 cells with suppressed PKLR by GSEA

1. Bubble plots showed three positively and eleven negatively correlated KEGG pathways significantly associated with PKLR overexpression;

B-P. Details on individualized results in each significant KEGG pathways by GSEA.

Abbreviations: GSEA, Gene Set Enrichment Analysis.


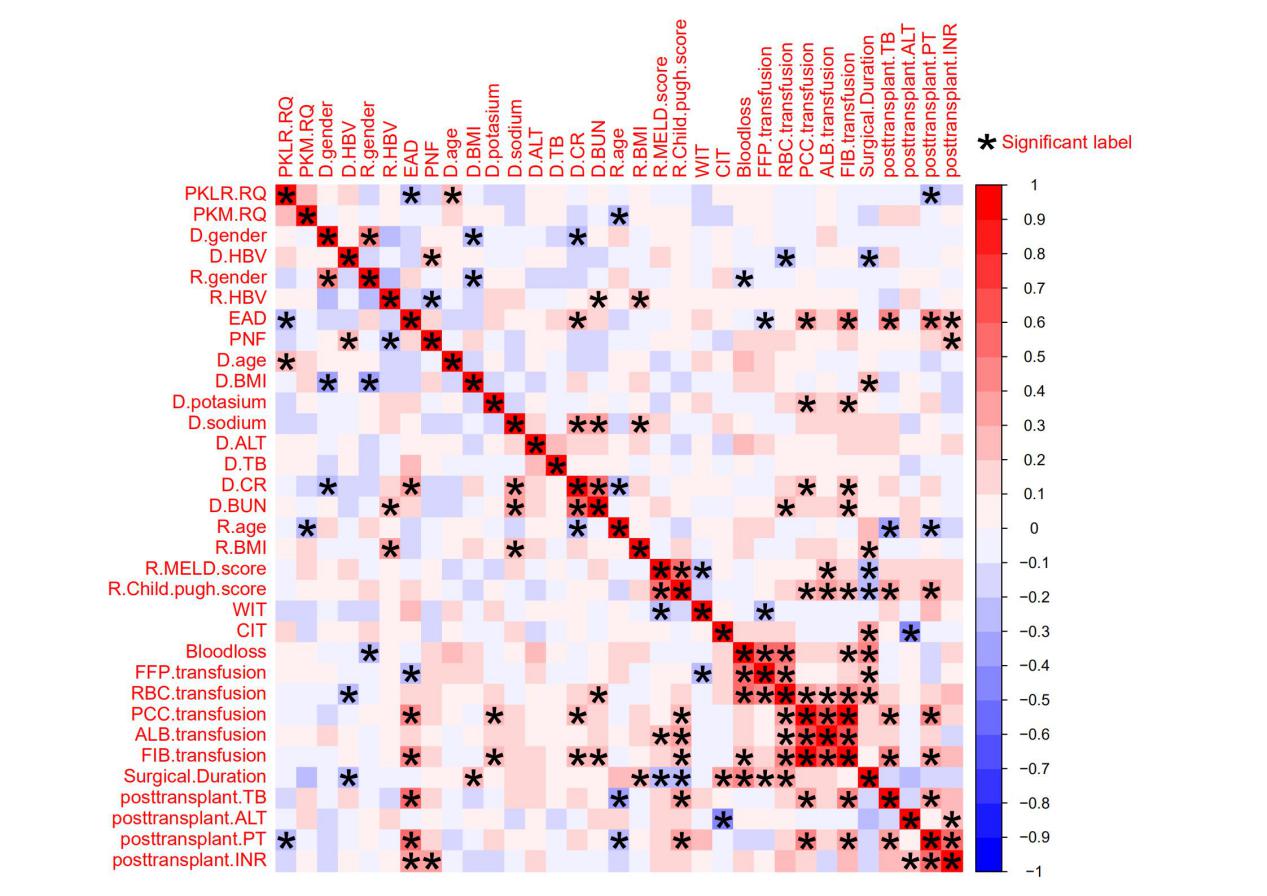


Figure S5 Correlation analysis for interconnection in matrix with inclusion of PKLR/PKM gene and all enrolled clinical indicators. * represented the positive connection between two indicators at statistical significance; the table is color coded by correlation according to the color legend; legend on intensity and direction of correlations are indicated on the right side of the heatmap.

Abbreviations: ALB, albumin; ALT, alanine aminotransferase; BMI, body mass index; BUN, blood urea nitrogen; CIT, cold ischemia time; CR, creatinine; D, donor; EAD, early allograft dysfunction; FFP, fresh frozen plasma; FIB, fibrinogen; HBV, hepatitis B virus; INR,International Normalized Ratio; MELD, Model for End-stage Liver Disease; PCC, prothrombin complex; PNF, primary liver graft nonfunction; PT, prothrombin Time; R, recipient; RBC, red blood cell; RQ, relative quantity; TB, total bilirubin; WIT, warm ischemia time.
